# MicroRNA‐181a restricts human γδ T cell differentiation by targeting Map3k2 and Notch2

**DOI:** 10.15252/embr.202052234

**Published:** 2021-11-24

**Authors:** Gisela Gordino, Sara Costa‐Pereira, Patrícia Corredeira, Patrícia Alves, Luís Costa, Anita Q Gomes, Bruno Silva‐Santos, Julie C Ribot

**Affiliations:** ^1^ Instituto de Medicina Molecular João Lobo Antunes Faculdade de Medicina Universidade de Lisboa Lisbon Portugal; ^2^ Medical Oncology Division Hospital de Santa Maria Centro Hospitalar Universitário Lisboa Norte Lisbon Portugal; ^3^ Escola Superior de Tecnologia da Saúde de Lisboa Lisbon Portugal

**Keywords:** cancer, effector T lymphocytes, microRNAs, miR‐181a, γδ T cells, Cancer, Immunology, RNA Biology

## Abstract

γδ T cells are a conserved population of lymphocytes that contributes to anti‐tumor responses through its overt type 1 inflammatory and cytotoxic properties. We have previously shown that human γδ T cells acquire this profile upon stimulation with IL‐2 or IL‐15, in a differentiation process dependent on MAPK/ERK signaling. Here, we identify microRNA‐181a as a key modulator of human γδ T cell differentiation. We observe that miR‐181a is highly expressed in patients with prostate cancer and that this pattern associates with lower expression of NKG2D, a critical mediator of cancer surveillance. Interestingly, miR‐181a expression negatively correlates with an activated type 1 effector profile obtained from *in vitro* differentiated γδ T cells and miR‐181a overexpression restricts their levels of NKG2D and TNF‐α. Upon *in silico* analysis, we identify two miR‐181a candidate targets, Map3k2 and Notch2, which we validate *via* overexpression coupled with luciferase assays. These results reveal a novel role for miR‐181a as critical regulator of human γδ T cell differentiation and highlight its potential for manipulation of γδ T cells in next‐generation immunotherapies.

## Graphical abstract

The miR‐181a targets Map3k2 and Notch2 and thereby regulates human γδ T cell differentiation. This limits the expression of NKG2D and TNF‐α, both involved in γδ T cell‐mediated cancer surveillance.

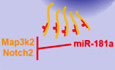

## Introduction

Despite the recent advances in therapeutic strategies against cancer, early tumor recurrence and novel metastasis formation indicate resistance to current treatments. This urges the development of alternative treatments for advanced stages of the disease. γδ T cells possess multiple anti‐tumor characteristics, making them promising candidates to be used in cellular and combination therapies (Gentles *et al*, 2015; Silva‐Santos *et al*, 2015, 2019). They provide IFN‐γ‐associated type 1 responses against cancer and express critical determinants of tumor cell recognition, including their signature γδ TCR but also a variety of NK cell receptors (NKRs), among which the natural killer group 2 member D (NKG2D) is of utmost importance (Lança *et al*, 2010; Correia *et al*, 2013; Silva‐Santos *et al*, 2015; Wu *et al*, 2019). This notwithstanding, the γδ T cell‐based clinical trials completed to date have shown objective responses of only 10–33% (Gomes *et al*, 2010; Lo Presti *et al*, 2017). This modest outcome could be explained by their deficient expansion and/or dysregulated effector functions *in vivo* (Argentati *et al*, 2003; Bryant *et al*, 2009; Gaafar *et al*, 2009; Kuroda *et al*, 2012).

Parallel investigations in both mice and humans have suggested that the tumor microenvironment can subvert the anti‐tumor type 1 effector γδ T cell phenotype, either inactivating it (Marten *et al*, 2006; Gonnermann *et al*, 2015) or diverting into immunosuppressive phenotypes (Peng *et al*, 2007; Hao *et al*, 2011; Ma *et al*, 2012; Ye *et al*, 2013a, 2013b) or promoting the expansion of distinct pro‐tumor type 17 effector γδ T cells (Rei *et al*, 2014; Wu *et al*, 2014; Silva‐Santos *et al*, 2019). Collectively, these limitations stress the need to restore or enhance γδ T cell type 1 inflammatory and cytotoxic properties in future immunotherapeutic approaches.

Based on this background, we have previously demonstrated that the anti‐tumor type 1 effector properties of γδ T cells are selectively acquired upon stimulation with IL‐2 or IL‐15, but not IL‐4 or IL‐7 (Ribot *et al*, 2014). The effects of IL‐2/IL‐15 depended on MAPK/ERK signaling and induced *de novo* expression of the type 1 transcription factors T‐bet and eomesodermin. We followed those studies by exploring an additional layer of regulation of γδ T cell differentiation, namely, post‐transcriptional mechanisms mediated by microRNAs (miRNAs or miRs), which are still poorly characterized in γδ T cells (Fiala *et al*, 2020), especially in the human setting (Zhu *et al*, 2017).

MiRNAs are naturally occurring and evolutionarily conserved endogenous small non‐coding RNAs (18–25 nucleotides) that typically downregulate post‐transcriptional gene expression by binding to the 3ʹ untranslated region (UTR) of their target mRNAs and promoting their degradation or inhibiting their translation (Ambros, 2004). Each precursor miRNA consists of two mature RNA sequences—the 5p and 3p strands—whose designation is attributed according to the directionality of the miRNA strand (Kozomara & Griffiths‐Jones, 2014). Although it has long been proposed that the 5p strand is the one being loaded into the RNA‐induced silencing complex (RISC), recent evidence has disproven the idea that the 3p strand is mainly degraded during miR biogenesis (Kozomara *et al*, 2019). In fact, both the 5p and 3p strands can be loaded onto the Argonaute (AGO) family of proteins in an ATP‐dependent manner (Yoda *et al*, 2010) and can show differential expression levels according to the pathophysiological context under study, namely, in cancer (Mitra *et al*, 2015, 2020). Importantly, miRNAs exert their regulatory functions in a highly combinatorial way: One miRNA can regulate several mRNAs in parallel, and different miRNAs can target one mRNA simultaneously, thus repressing its expression more efficiently (Pons‐Espinal *et al*, 2017). Since this post‐transcriptional process controls the expression of most mammalian genes, it is particularly relevant to analyze its role in human γδ T cell differentiation. To date, only one study has highlighted a role for miR‐125b‐5p and miR‐99a‐5p in human γδ T cell activation and cytotoxicity (Zhu *et al*, 2017), while the involvement of other miRs and their potential impact on γδ T cell responses to cancer remains unclear.

Here, we identify miR‐181a as a novel molecular regulator of human γδ T cell functional differentiation. We demonstrate that both its ‐5p and ‐2‐3‐p strands control γδ T cell type 1 effector differentiation and responsiveness by targeting Map3k2 and Notch2 mRNAs, and suggest a potential implication of this process in metastatic cancer patients.

## Results and Discussion

### miR‐181a is upregulated in peripheral γδ T cells from metastatic cancer patients

**Figure 1:**
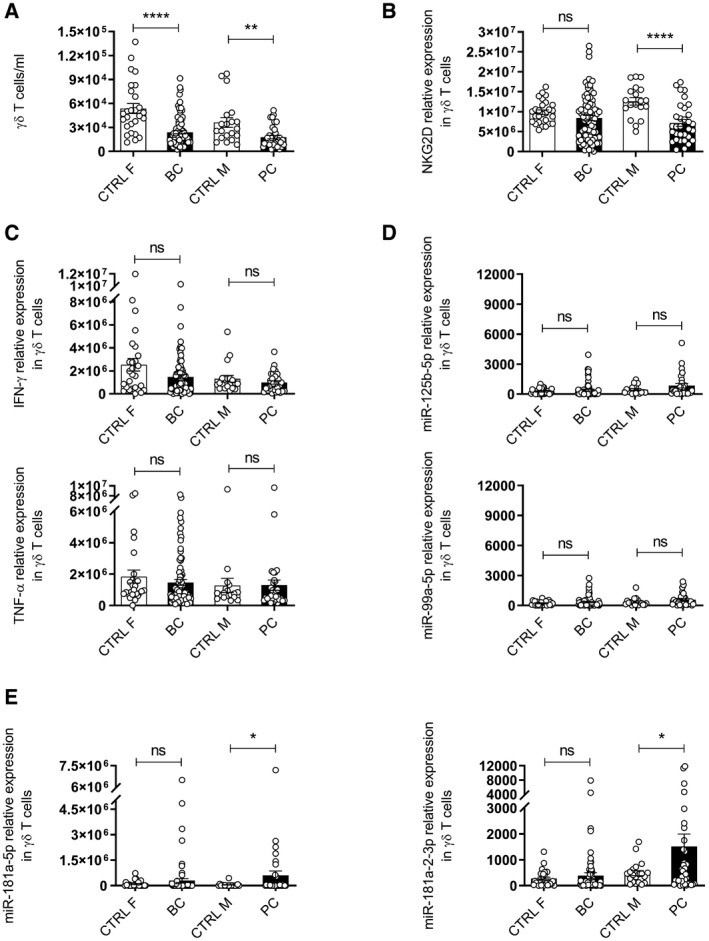
Peripheral γδ T cells from metastatic cancer patients display impaired effector functions and increased expression of miR‐181a γδ T cell concentration in the peripheral blood from healthy donors and indicated metastatic cancer patients.RT–PCR analysis of the expression of NKG2D in γδ T cells isolated from indicated samples.RT–PCR analysis of the expression of IFN‐γ and TNF‐α in γδ T cells isolated from the peripheral blood of healthy controls and metastatic cancer patients.RT–PCR analysis of the expression of miR‐125b‐5p and miR‐99a‐5p in γδ T cells isolated from indicated samples.RT–PCR analysis of the expression of miR‐181a(‐5p and ‐2‐3p) in γδ T cells isolated from indicated samples. Data information: (A–E) Error bars represent the mean ± SEM. CTRL F = Control Females (*n* = 25–27 independent biological samples); BC=Breast Cancer (*n* = 79–83 independent biological samples); CTRL M = Control Males (*n* = 19–20 independent biological samples); PC = Prostate Cancer (*n* = 29–36 independent biological samples). Statistical analysis was performed using the unpaired Student's *t*‐test with Welch's correction. ns, not significant. *P < 0.05, **P < 0.01, and ****P < 0.0001. All experiments were performed with two technical replicates. Source data are available online for this figure.

We investigated potential associations between miRNA expression patterns and γδ T cell dysfunction in metastatic cancer patients compared to healthy controls. Reduced number and impaired pro‐inflammatory cytokine production have been previously reported in circulating γδ T cells from patients with melanoma (Argentati *et al*, 2003), glioblastoma (Bryant *et al*, 2009), breast (Gaafar *et al*, 2009), and gastric (Kuroda *et al*, 2011) carcinomas. Here, we analyzed patients of the cancer types with highest incidence in women (breast) and men (prostate), both in the metastatic (stage IV) setting. We found lower numbers of γδ T cells in the peripheral blood of both cohorts, and a decrease in their expression of NKG2D (significant in the prostate cohort), when compared to the respective (female or male) healthy controls (Fig 1A and B). We also observed a tendency for reduced IFN‐γ production in the peripheral γδ T cells from the breast cancer patient cohorts, but this did not reach statistical significance (Fig 1C). Whereas the expression of miR‐125b‐5p and miR‐99a‐5p, which have previously been reported to modulate γδ T cell activation and cytotoxicity (Zhu *et al*, 2017), was very low and comparable to healthy controls (Fig 1D), we found—among other candidates under study—miR‐181a‐5p and miR‐181a‐2‐3p to be upregulated in metastatic cancer patients, especially in the prostate cancer cohort (Fig 1E), thus providing an interesting association with γδ T cell dysfunction (Fig 1A and B). Of note, the effects observed were independent of patient age and treatment history.

miR‐181a is known for its pleiotropic functions on αβ T cell differentiation, including controlling the Th1 response of human CD4^+^ T cells (Sang *et al*, 2015); however, the role of miR‐181a has never been addressed in human γδ T cells.

### miR‐181a is downregulated upon type 1 effector γδ T cell differentiation

**Figure 2:**
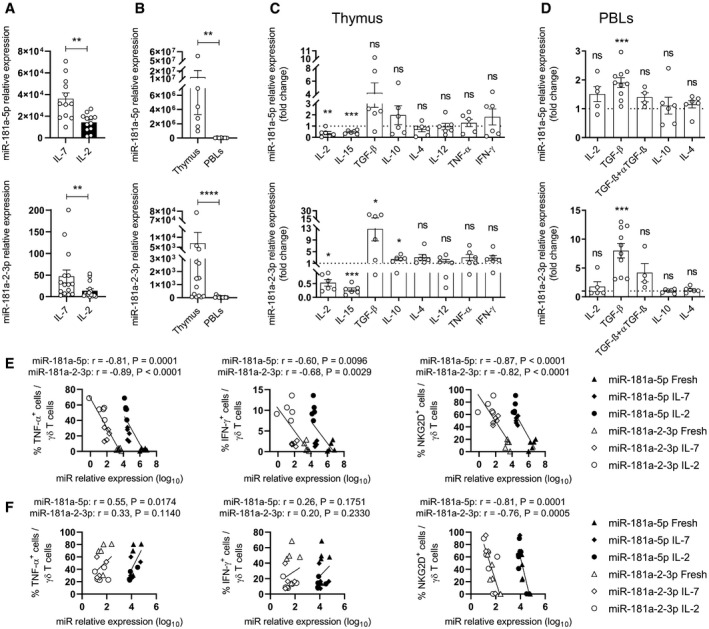
miR‐181a is downregulated upon type 1 effector γδ T cell differentiation ART–PCR analysis of the expression of miR‐181a(‐5p and ‐2‐3p) in γδ T cells isolated from thymic biopsies, cultured for 11 days with IL‐7 or IL‐2 (*n* = 12–15 independent biological samples).BRT–PCR analysis of the expression of miR‐181a(‐5p and ‐2‐3p) in γδ T cells freshly isolated from thymic biopsies (Thymus) and PBLs (*n* = 6–14 independent biological samples).C, DRT–PCR analysis of the expression of miR‐181a(‐5p and ‐2‐3p) in γδ T cells isolated from thymus (C) or from PBLs (D) and cultured with the indicated cytokines, respectively for 11 days (C) or for 4–6 days (D). When indicated, γδ PBLs were also co‐cultured with an anti‐TGF‐β blocking antibody. Data are normalized to the value obtained in IL‐7 cultures (*n* = 4–10 independent biological samples).E, FRT–PCR analysis of the expression of miR‐181a(‐5p and ‐2‐3p) versus FACS analysis of the expression of TNF‐α (left panel), IFN‐γ (middle panel), and NKGD2 (right panel) in γδ T cells isolated from thymus (E) and PBLs (F). Samples were either freshly isolated or cultured for 11 days (γδ thymocytes) or for 6 days (γδ PBLs) with the indicated cytokines (*n* = 5 independent biological samples). Data information: (A–D) Error bars represent the mean ± SEM. (A, C, D) Statistical analysis was performed using the paired Student's *t*‐test. (B) Statistical analysis was performed using the Mann–Whitney *U* test. (E, F) The Pearson's correlation coefficient (*r*) was used to measure the strength of association between two variables. ns, not significant. *P < 0.05, **P < 0.01, ***P < 0.001, and ****P < 0.0001. All experiments were performed with two technical replicates. Source data are available online for this figure.

**Figure EV1:**
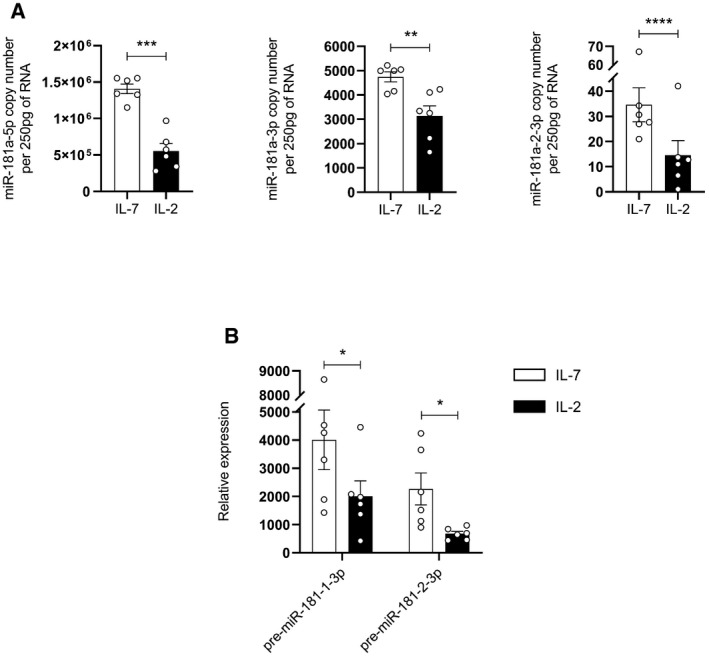
miR‐181a‐5p and 3p strands are downregulated upon IL‐2 stimulation RT–PCR analysis of miR‐181a‐5p, miR‐181a‐1‐3p and miR‐181a‐2‐3p copy numbers in γδ thymocytes cultured with IL‐7 versus IL‐2 (*n* = 6 independent biological samples).RT–PCR analysis of pre‐miR‐181a‐1‐3p and pre‐miR‐181a‐2‐3p expression in γδ thymocytes cultured with IL‐7 versus IL‐2 (*n* = 6 independent biological samples). Data information: Error bars represent the mean ± SEM. Statistical analysis was performed using the paired Student's *t*‐test. *P < 0.05, **P < 0.01, ***P < 0.001, and ****P < 0.0001. All experiments were performed with two technical replicates. Source data are available online for this figure.

Given each individual's history of infections, circulating γδ T cells in healthy donors are mostly functionally mature cells (Gibbons *et al*, 2009; deBarros *et al*, 2011). By contrast, we have previously shown that human γδ thymocytes isolated from pediatric biopsies of thymus are functionally immature and can be induced to acquire a type 1/cytotoxic profile upon stimulation with IL‐2 or IL‐15, but not IL‐4 or IL‐7 (Ribot *et al*, 2014). Based on our data described above, we hypothesized that miR‐181a might be downregulated during type 1 effector differentiation of human γδ thymocytes. Consistent with our hypothesis, we observed that the expression of both miR‐181a strands was significantly lower in *in vitro* differentiated (IL‐2 cultured) γδ thymocytes compared to immature (IL‐7 cultured) controls (Fig 2A). In fact, all miR‐181a species, including pre‐miRNAs and mature 5p and 3p strands, were downregulated upon IL‐2 treatment (Fig EV1A and B). Moreover, this downregulation of miR‐181a (‐5p and 2‐3p) expression was also found when comparing freshly isolated (immature) γδ thymocytes *versus* (mature) peripheral γδ T cells *ex vivo* (Fig 2B).

**Figure EV2:**
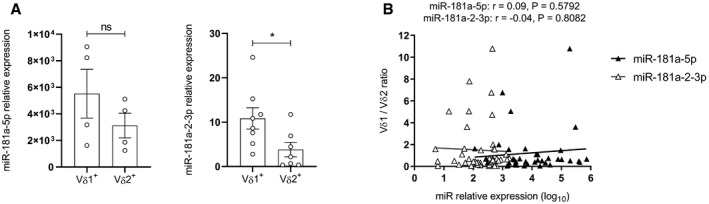
miR‐181a expression in Vδ1^+^ versus Vδ2^+^ γδ T cells RT–PCR analysis of the expression of miR‐181a(‐5p and ‐2‐3p) in freshly isolated Vδ1^+^ versus Vδ2^+^ sorted γδ PBLs (*n* = 4–8 independent biological samples).Correlation between Vδ1/Vδ2 ratio versus miR‐181a(‐5p and ‐2‐3p) expression in freshly isolated γδ PBLs (*n* = 39–46 independent biological samples). Data information: (A) Error bars represent the mean ± SEM. Statistical analysis was performed using the unpaired Student's *t*‐test. (B) The Pearson's correlation coefficient (*r*) was used to measure the strength of association between two variables. ns, not significant. *P < 0.05. All experiments were performed with two technical replicates. Source data are available online for this figure.

Given that human γδ T cells comprise two major subsets, Vδ1^+^ cells, that are more abundant in the thymus and non‐lymphoid tissues, and Vδ2^+^ cells, which represent 60–95% of γδ T cells in the peripheral blood and in lymph nodes (Fichtner *et al*, 2020), we assessed whether the composition of these populations could influence the levels of miR‐181a expression in total γδ T cell samples. Although we found a higher level of miR‐181a expression in the Vδ1^+^ γδ T cell subset (Fig EV2A), the Vδ1/Vδ2 ratio did not correlate with miR‐181a expression levels (Fig EV2B). We next anticipated that, besides IL‐2, other cytokines might regulate miR‐181a expression and thus cultured γδ thymocytes with a set of pro‐ and anti‐inflammatory mediators. IL‐7 was used as control condition, allowing cell survival without promoting their functional differentiation (Ribot *et al*, 2014). Like IL‐2, IL‐15, which is also known to promote γδ T cell functional differentiation (Ribot *et al*, 2014), substantially downregulated the expression of both miR‐181a strands (Fig 2C). By contrast, TGF‐β upregulated miR‐181a expression, while other cytokines such as IL‐4, IL‐12, IFN‐γ, and TNF‐α showed no impact (Fig 2C). Collectively, these data revealed that miR‐181a expression is downregulated by drivers of type 1 effector γδ T cell differentiation, while being induced by immunosuppressive cytokines, namely, TGF‐β, which is typically enriched in the cancer setting (Batlle & Massagué, 2019). This may have pathophysiological relevance, as TGF‐β significantly enhanced miR‐181a expression in γδ T cells isolated from the peripheral blood of healthy donors (Fig 2D), thus mimicking the increased miR‐181a levels displayed by γδ T cells from patients with prostate cancer (Fig 1E).

**Figure EV3:**
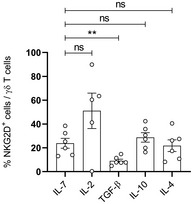
TGF‐β signals reduce NKG2D expression FACS analysis of the expression of NKGD2 in γδ T cells isolated from PBLs cultured with the indicated cytokines for 4–6 days (*n* = 5–6 independent biological samples, paired Student's *t*‐test). Data represent the mean ± SEM. ns, not significant. **P < 0.01. All experiments were performed with two technical replicates. Source data are available online for this figure.

To further document the negative association of miR‐181a with type 1 effector γδ T cells, we next analyzed the expression of molecular hallmarks in various γδ T cell samples at different stages of differentiation. Namely, we used the same samples (i.e., freshly isolated versus *in vitro* IL‐7‐ or IL‐2‐cultured γδ thymocytes) to measure the expression of miR‐181a‐5p and ‐2‐3p, as well as the surface protein levels of the type 1 cytotoxic mediator NKG2D, and the intracellular expression of the type 1 cytokines, IFN‐γ and TNF‐α. We observed a striking inverse correlation between the expression of both miR‐181a strands and the percentages of γδ thymocytes positive for IFN‐γ, TNF‐α, and NKG2D (Fig 2E), fully consistent with our hypothesis that miR‐181a negatively regulates type 1 effector γδ T cell differentiation. Interestingly, this inverse correlation was not sustained in *ex vivo* peripheral blood γδ T cells (Fig 2F), suggesting a role for miR‐181a during the early differentiation process, after which cytokine production becomes constitutive in mature cells. On the other hand, NKG2D showed a more dynamic profile in peripheral γδ T cells, which maintained the inverse correlation between NKG2D and miR‐181a levels (Fig 2F) as observed in γδ thymocytes (Fig 2E). Consistently, the upregulation of miR‐181a expression observed in TGF‐β cultured peripheral γδ T cells (Fig 2D) associated with a lower percentage of NKG2D^+^ cells (Fig EV3). While this may suggest a segregation of the molecular mechanisms that regulate NKG2D and the pro‐inflammatory cytokines in fully mature peripheral γδ T cells, our results collectively pointed toward a role for miR‐181a during effector γδ T cell differentiation, which we set out to test using a gain‐of‐function approach.

### miR‐181a overexpression impairs γδ T cell differentiation

**Figure 3:**
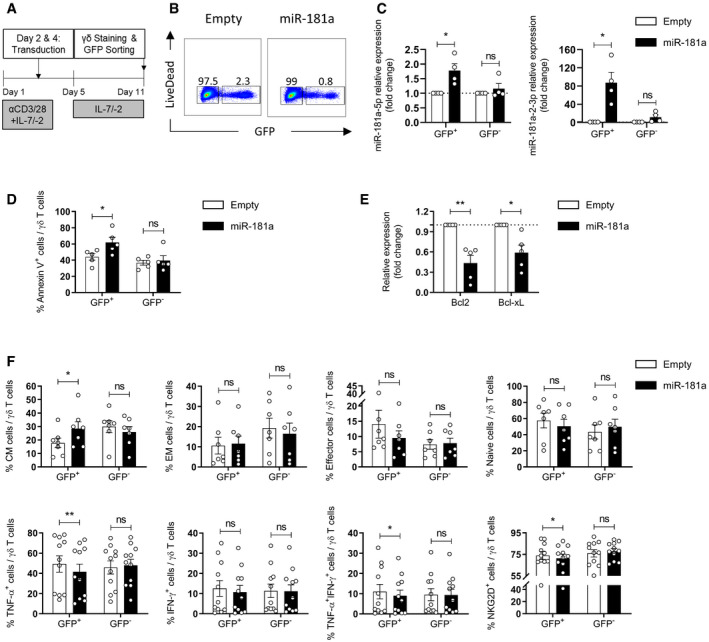
miR‐181a overexpression impairs γδ T cell functional differentiation Retroviral transduction workflow for γδ thymocytes.Gating strategy for identification of the GFP^+^ versus GFP^−^ cells.RT–PCR analysis of the expression of miR‐181a(‐5p and ‐2‐3p) (normalized to the values obtained with the empty virus) in transduced (GFP^+^) and untransduced (GFP^−^) γδ thymocytes, cultured with IL‐7 plus IL‐2 for 11 days (*n* = 4 independent biological samples).FACS analysis of the expression of Annexin V in miR‐181a versus empty transduced (GFP^+^) and untransduced (GFP^−^) γδ thymocytes, cultured with IL‐7 plus IL‐2 for 11 days (*n* = 5 independent biological samples).RT–PCR analysis of the expression of Bcl2 and Bcl‐xL (normalized to the values obtained with the empty virus) in transduced (GFP^+^) γδ thymocytes, cultured with IL‐7 plus IL‐2 for 11 days (*n* = 5 independent biological samples).FACS‐derived expression of indicated surface and intracellular markers in miR‐181a versus empty transduced (GFP^+^) and untransduced (GFP^−^) γδ T thymocytes, cultured with IL‐7 plus IL‐2 for 11 days (*n* = 7–11 independent biological samples). Data information: (C–F) Error bars represent the mean ± SEM. Statistical analysis was performed using the paired Student's *t*‐test. ns, not significant. *P < 0.05 and **P < 0.01. All experiments were performed with two technical replicates. Source data are available online for this figure.

To formally test whether miR‐181a is able to regulate effector γδ T cell differentiation, we used retroviral transduction to overexpress miR‐181a. We designed a construct in order to overexpress the native stem‐loop of miR‐181a (containing both ‐5p and ‐2‐3p strands) in γδ thymocyte cultures. Cells were freshly isolated, activated, transduced, and maintained in the presence of IL‐7 and IL‐2 to promote type 1 cytotoxic differentiation (Fig 3A). The expression of the GFP reporter allowed us to identify the subpopulation that integrated the retroviral construct (Fig 3B). As a technical control of this set of experiments, we verified that sorted GFP^+^ cells transduced with miR‐181a vector displayed a significant increase in the expression of miR‐181a (both the ‐5p and ‐2‐3p strands) when compared to cells transduced with the empty virus (Fig 3C). On the other hand, as an internal control, we consistently analyzed the untransduced GFP^−^ population, where miR‐181a expression remained at baseline (Fig 3C), and thus, no changes in any of the below‐mentioned readouts were expected.

miR‐181a overexpression seemingly impaired γδ T cell survival, given the increase in Annexin V^+^ cells (Fig 3D) and the reduction in expression of the anti‐apoptotic genes Bcl2 and Bcl‐xL (Fig 3E) among the transduced (but not the untransduced) γδ T cells. On the other hand, miR‐181a overexpression increased the percentage of cells with a central memory profile (CD27^+^CD45RA^−^), while reducing the percentage of cells displaying a typical naïve (CD27^+^CD45RA^+^) and effector phenotype (CD27^−^CD45RA^+^; Fig 3F). Since previous studies reported that memory T cells arise from the presence of high concentration levels of IL‐2 (Berard & Tough, 2002; Yurova *et al*, 2017), our results support a functional crosstalk between miR‐181a and IL‐2‐dependent signaling.

**Figure EV4:**
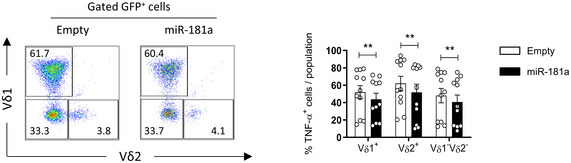
miR‐181a overexpression impact on TNF‐α cytokine production in different γδ T cell subpopulations Gating strategy for the identification of the Vδ1 versus Vδ2 subpopulations in miR‐181a versus empty transduced (GFP^+^) γδ thymocytes (left panel) and TNF‐α expression gated on either Vδ1^+^, Vδ2^+^, or Vδ1^−^Vδ2^−^ populations, in (GFP^+^) miR‐181a versus empty transduced γδ thymocytes, cultured with IL‐7 plus IL‐2 for 11 days (right panel, *n* = 11 independent biological samples). Data Information: Error bars represent the mean ± SEM. Statistical analysis was performed using the paired Student's *t*‐test. **P < 0.01. All experiments were performed with two technical replicates. Source data are available online for this figure.

From a functional standpoint, we demonstrated that miR‐181a overexpression led to significant reductions in the percentages of TNF‐α^+^ and NKG2D^+^ γδ T cells (Fig 3F). Interestingly, while the percentage of IFN‐γ^+^ γδ T cells was not affected, we observed a decrease in the percentage of TNF‐α^+^ IFN‐γ^+^ subset, suggesting that, in the type 1 effector differentiation pathway, miR‐181a could also regulate a step where IFN‐γ production would be induced from the TNF‐α^+^ population, as previously proposed for Vδ2^+^ γδ T cells and NK cells (Skeen & Ziegler, 1995; Li *et al*, 2008). Of note, we observed that miR‐181a overexpression equally affected the percentage of TNF‐α^+^ cells within Vδ1^+^ and Vδ2^+^ population (Fig EV4).

**Figure EV5:**
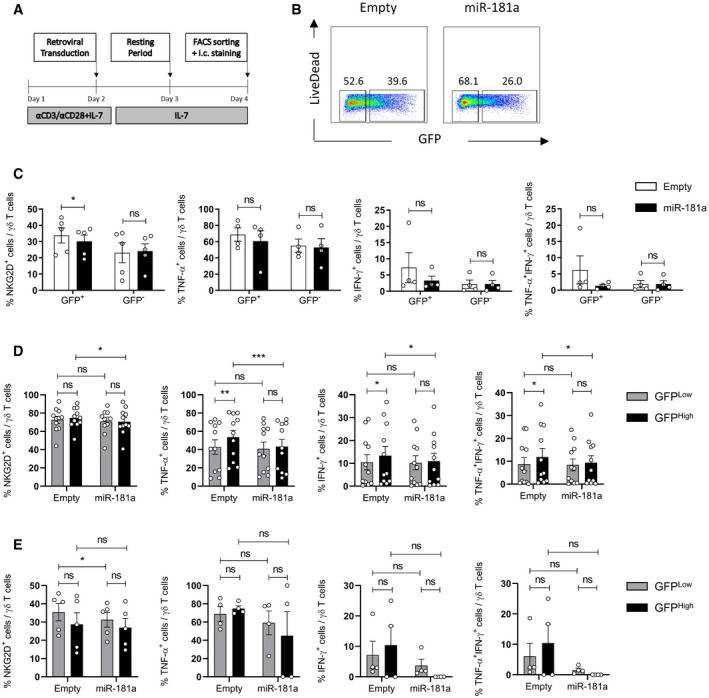
Absence of dose effect of miR‐181a‐bearing vector transduction in γδ T PBLs and thymocytes ARetroviral transduction workflow for γδ PBLs.BGating strategy for the identification of the GFP^+^ versus GFP^−^ cells.CFACS analysis of the expression of indicated surface and intracellular markers in miR‐181a versus empty transduced (GFP^+^) and untransduced (GFP^−^) γδ PBLs, cultured with IL‐7 for 4 days (*n* = 4–5 independent biological samples).D, EImpact of miR‐181a levels on γδ T cells isolated from (D) thymus and (E) PBLs. GFP^Low^ and GFP^High^ subsets were analyzed in miR‐181a versus empty transduced γδ T cells for their expression of NKG2D, TNF‐α and IFN‐γ. γδ T thymocytes were cultured with IL‐7 plus IL‐2 for 11 days and γδ PBLs were cultured with IL‐7 for 4 days (*n* = 11 and *n* = 4–5 independent biological samples, respectively). Data Information: (C–E) Error bars represent the mean ± SEM. Statistical analysis was performed using the paired Student's *t*‐test. ns, not significant. *P < 0.05, **P < 0.01, and ***P < 0.001. All experiments were performed with two technical replicates. Source data are available online for this figure.

We further overexpressed miR‐181a in mature γδ T cells isolated from healthy donor peripheral blood (Fig EV5A and B) and found a significant albeit minor decrease in NKG2D levels, while type 1 differentiation program remained intact (Fig EV5C). This is consistent with our previous data pointing at an inverse correlation in peripheral γδ T cells between the expression of miR‐181a and NKG2D, but not type 1 cytokine expression (Fig 2F). Of note, by comparing GFP^Low^ and GFP^High^ subsets, either in γδ thymocyte (Fig EV5D) or PBL (Fig EV5E) cultures, we did not observe any dose effect of the levels of miR‐181a‐bearing vector transduction. Overall, the differences between PBL and thymocyte cultures suggest that the maturation status of γδ T cells conditions their sensitivity to miR‐181a action, which is maximized at early stages of functional γδ T cell differentiation.

### miR‐181a targets Map3k2 and Notch2 mRNAs

To dissect the molecular mechanisms underlying miR‐181a function, we went on to identify putative mRNA targets. For this, a bioinformatical analysis using the mirDIP v4.1 database, coupled to a Gene Set Enrichment Analysis (GSEA) of candidates involved in Th1 differentiation and dysregulated in cancer, allowed us to predict a list of candidates based on the miR‐181a‐5 and ‐2‐3p mature sequences, common to both strands (Dataset EV1). Interestingly, we could highlight the Notch and Mapk signaling pathways that were already described to regulate the functional differentiation of mature T lymphocytes (Ribot *et al*, 2014; Amsen *et al*, 2015; Duval *et al*, 2015). Based on their putative binding sites, Map3k2 and Notch2 were thus selected as potential miR‐181a(‐5p and ‐2‐3p) targets.

**Figure 4:**
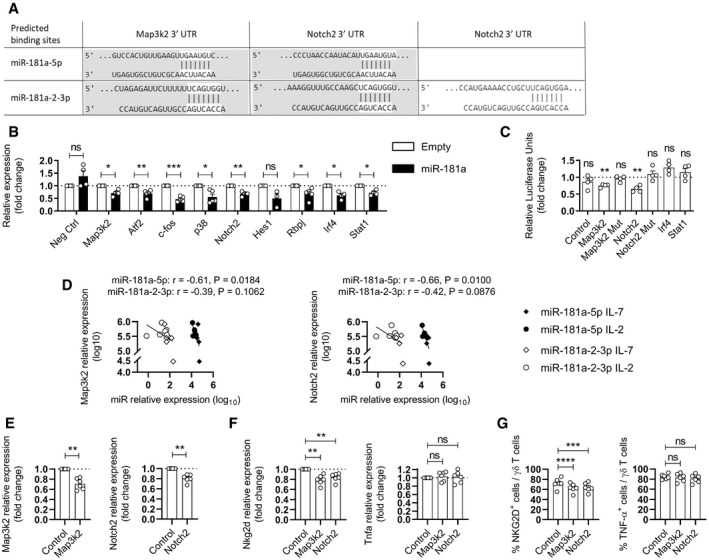
miR‐181a targets Map3k2 and Notch2 mRNAs APutative binding sites of miR‐181a(‐5p and ‐2‐3p) in the 3′ UTR region of Map3k2 and Notch2. Potential miR‐181a(‐5p and ‐2‐3p) gene targets were selected based on predictions obtained in referenced mirDIP v4.1database. The predicted binding regions that were amplified for the luciferase assay are highlighted in gray.BRT–PCR analysis of the expression of the indicated genes associated with Mapk, Notch, Irf4, or Stat1 signaling in sorted GFP^+^ γδ thymocytes transduced with empty or miR‐181a viral vector. Data are normalized to the value obtained with the empty virus. Samples were cultured with IL‐7 plus IL‐2 for 11 days (*n* = 3–5 independent biological samples).CDual luciferase reporter assay performed to verify binding between miR‐181a and Map3k2, Notch2, Irf4, or Stat1 mRNA. HEK293 T cells were co‐transfected with a pmirGLO Dual‐Luciferase miRNA Target Expression Vector containing either the WT or mutated (Mut) 3′ UTR target sites plus miR‐181a. A negative construct (without miR‐181a binding sites) was included. Firefly/Renilla ratios were normalized to those obtained for the empty vector (*n* = 4 independent biological samples).DRT–PCR analysis of the expression of miR‐181a(‐5p and ‐2‐3p) versus MAP3K2 (left panel) or NOTCH2 (right panel) expression in γδ T cells isolated from thymic biopsies. Samples were cultured for 11 days with the indicated cytokines (*n* = 6 independent biological samples).E–GRT–PCR analysis in circulating γδ T cells of Map3k2 or Notch2 expression (E), Nkg2d or Tnfa expression (F), and NKG2D or TNF‐α protein expression (G) after siRNA‐mediated knockdown of Map3k2 or Notch2. In each experiment, a control condition was also used where a nontargeting negative control siRNA was transfected. γδ were cultured with IL‐7 for 4 days (*n* = 6 independent biological samples). Data information: (B, C, E–G) Error bars represent the mean ± SEM. Statistical analysis was performed using the paired Student's *t*‐test. (D) The Pearson's correlation coefficient (*r*) was used to measure the strength of association between two variables. ns, not significant. *P < 0.05, **P < 0.01, ***P < 0.001, and ****P < 0.0001. All experiments were performed with two technical replicates. Source data are available online for this figure.

To experimentally validate these bioinformatics predictions, γδ thymocytes were transduced with either the empty or the miR‐181a viral vector and then differentiated in the presence of IL‐2. We next sorted the GFP^+^ transduced fraction and analyzed the expression of Map3k2, Notch2, and Ptbp1 (as negative control) by RT–PCR. As predicted, miR‐181a overexpression in the type 1 differentiation protocol induced a significant decrease in Map3k2 and Notch2 mRNA levels (Fig 4A and B). We also analyzed the expression of genes involved in both MAPK (namely Atf2 and c‐Fos) and NOTCH (namely Hes1) signaling pathways and observed a reduction of the expression of all these signature genes within γδ T cells transduced with miR‐181a, when compared to the empty control vector (Fig 4B). We further noticed a reduction of additional genes associated with both pathways, namely, p38 and Rbpj (Fig 4B). Given the described role for both these genes as key regulators of DNA methylation and histone acetylation processes (Clark *et al*, 2003; Schmeck *et al*, 2005; Giaimo *et al*, 2017; Rozenberg *et al*, 2018), such results point to an alternative upstream transcriptional regulation or epigenetic alterations that can potentially be modulated by miR‐181a upregulation. To assess direct binding between miR‐181a and its target mRNAs, Map3k2, and Notch2, we designed reporter constructs in a pmirGLO Dual‐Luciferase to include the Map3k2 or Notch2 3′ UTR target regions for both miR‐181a‐5p and ‐2‐3p species (Fig 4A). However, due to the technical limitations to include the entire region of interest from the 3′ UTR Notch2 candidate, we decided to amplify the binding site with the higher percentile context score, as predicted by the TargetScan v8.0 (Fig 4A). We included an additional negative control vector containing a 3′ UTR without miR‐181a binding sites. We transiently transfected these constructs into HEK293T cells together with a vector plasmid for either miR‐181a or an empty control. Importantly, co‐transfection of miR‐181a could elicit a significant repression of the luciferase activity of both Map3k2 and Notch2 mRNA targets, while not affecting the luciferase activity of the control mRNA vector (Fig 4C). By contrast, no repression was observed for the candidates or the control mRNAs in cells co‐transfected with the empty control. Of note, based on the target score obtained from our *in silico* analysis (Dataset EV1), we also assessed other promising candidate genes, namely, Irf4 and Stat1, two transcription factors crucial for T cell differentiation (O'Shea *et al*, 2011; Huber & Lohoff, 2014). While the expression of these genes was downregulated in miR‐181a transduced γδ T cells (Fig 4B), neither Irf4 nor Stat1 were validated as miR‐181a targets when first tested in luciferase assays (Fig 4C).

We performed additional luciferase assays by amplifying the 3′ UTR regions of Map3k2 and Notch2 in both miR‐181a‐5p and ‐2‐3p potential binding sites, and included a control condition where the amplified 3′ UTR region was mutated in both binding sites. This set of experiments allowed to validate that miR‐181a directly targets Map3k2 and Notch2 (Fig 4C). Here, it is important to highlight the fact that having amplified a 3′ UTR sequence containing a potential binding site for both miR‐181a‐5p and ‐2‐3p species makes it impossible to clearly define which, or if both strands, regulate Map3k2 and Notch2 mRNA expression. Analogously, the simultaneous mutation of the predicted binding sites for both this miR strands leading to a complete derepression, does not provide a strict answer, and individual luciferase assays where only one of the 3′ UTR predicted (‐5p or ‐2‐3p) binding sites is mutated would be required to address this question. This notwithstanding, we correlated the levels of expression of both miR‐181a‐5p and ‐2‐3p with Map3k2 and Notch2 in γδ thymocytes cultured with IL‐7 versus IL‐2, and could clearly find an inverse correlation for both miR‐181a‐5p and ‐2‐3p species (Fig 4D). Of note, only the correlations for the miR‐181a‐5p species reached a statistical significance. These data, together with the observation of a predominance of the 5p strand in our samples, suggest that Map3k2 and Notch2 are more likely repressed by miR‐181a‐5p in a physiological context.

Finally, we performed siRNA targeting Notch2 and Map3k2 and achieved a 20–30% reduction in the expression of these genes (Fig 4E). This strategy phenocopied the effect of miR‐181a overexpression on NKG2D expression in peripheral blood γδ T cells, at both the mRNA (Fig 4F) and protein (Fig 4G) levels. Altogether, these data identified Notch2 and Map3k2, two known mediators of T cell differentiation and cytotoxicity (Su *et al*, 2001; Amsen *et al*, 2007; Maekawa *et al*, 2008; Chang *et al*, 2011), as direct and relevant targets of miR‐181a in human γδ T cells. Interestingly, Map3k2 has been shown to negatively regulate TGF‐β‐mediated T cell differentiation, as Map3k2^−/−^ mice showed an abnormal accumulation of Treg cells in the periphery, with a subsequent decrease in IFN‐γ production (Chang *et al*, 2011). Additionally, genetic loss of Map3k2 *in vitro* abrogated IL‐2 production in T cells (Su *et al*, 2001). Likewise, Notch2 is well known to modulate T cell activation and differentiation. CD8^+^ T cells from Notch2‐deficient mice display impaired cytotoxic properties caused by the inability to form a transcriptional coactivator complex on the promoter of the gene encoding granzyme B (Maekawa *et al*, 2008). Notch signaling also contributes to the generation of human γδ thymocytes (Van Coppernolle *et al*, 2012) and regulates peripheral γδ T cell type 1 effector function and cytotoxic activity (Gogoi *et al*, 2014). Of note, the miR‐181 family has been previously described to have a key role in modulating Notch signaling, as deletion of miR‐181a‐1/b‐1 inhibits the development of Notch1 oncogene‐driven T cell acute lymphoblastic leukemia (Fragoso *et al*, 2012).

In sum, we propose that miR‐181a acts as a brake during γδ T cell development, preventing their functional differentiation in the human thymus. This could help explain why contrary to murine γδ T cells, which commit to effector functions in the thymus (Ribot *et al*, 2009), human γδ thymocytes remain functionally immature (Ribot *et al*, 2014).

The miR‐181 family encompasses six miRs, organized in three clusters: miR‐181a/b‐1, miR‐181a/b‐2, and miR‐181c/d (Zietara *et al*, 2013). Importantly, the mature 5p strands for miR‐181a‐1 and a‐2 as well as b‐1 and b‐2 display identical sequences (Winter & Krueger, 2019), indicating that these miRs might share some common regulatory functions. Within this family, miR‐181a/b‐1 is the most extensively studied, probably due to its dynamic expression throughout thymocyte development (Winter & Krueger, 2019). In mice, miR‐181a expression was shown to be higher in immature DP thymocytes (Neilson *et al*, 2007; Kirigin *et al*, 2012), but lower in more differentiated T cell populations, such as Th1 and Th2 effector cells (Li *et al*, 2007). Mice deficient in miR‐181a/b‐1 displayed a defect in thymic agonist selection (Zietara *et al*, 2013; Blume *et al*, 2016), impacting on invariant natural killer T (iNKT) cell and mucosal‐associated invariant T (MAIT) cell development (Zietara *et al*, 2013; Winter *et al*, 2019), while retaining normal numbers of γδ T cells (Sandrock *et al*, 2015).

miR‐181a is also known to regulate T cell functions in the context of graft‐versus‐host disease, where mice receiving donor T cells with enhanced miR‐181a expression remained healthy, whereas T cells lacking miR‐181a/b‐1 induced an exacerbated disease (Lee *et al*, 2016). Overexpression of different members from the miR‐181a family has been previously associated with an impairment of type 1 cytokine production in other immune partners. For instance, previous studies reported a decrease in IFN‐γ production upon overexpression of miR‐181a‐5p in murine CD8^+^ and human CD4^+^ T cells (Sang *et al*, 2015; Amado *et al*, 2020).

Several studies have highlighted the need to investigate the 3p strands, especially in a pathological context (Jazdzewski *et al*, 2009; Mitra *et al*, 2015, 2020; Pink *et al*, 2015; Misono *et al*, 2018). In fact, a recent pan‐cancer analysis has revealed a miR cooperativity of both (5p and 3p) strands to be able to regulate tumorigenesis and patient survival (Mitra *et al*, 2020). Here, we used specific primers to assess both miR‐181a‐5p and ‐2‐3p expression in different γδ T cell samples and observed a consistent higher expression of the ‐5p compared to the ‐2‐3p strand. Of note, a recent elegant study in mouse shows that miRNA activity is dependent on its concentration and thus suggests that miRNA signatures should incorporate abundance thresholds to establish their regulatory relevance (Rose *et al*, 2021).

Consistently, based on a mathematical model‐derived predictions to describe gene regulation on the single cell level, another pioneer study had previously described that low expressed miRNA below median have low or no inhibitory capability, while miRNAs over the median and third quartile show a range of behavior that does not reflect their expression levels (Lemus Diaz *et al*, 2017). Of note, the authors propose that the gene regulatory function is actually a dynamic and complex process, in which some miRNAs also display their functionality according to the subcellular location, with low functional and high expressed miRNA being at least partially located in the nucleus, while some mid expressed but high functional miRNAs are mainly seen in the cytoplasmic region (Lemus Diaz *et al*, 2017).

Albeit the fact that miR‐181a‐5p was by far the most predominant species present in our study, as the variation in expression of both miR‐181a‐5p and ‐2‐3p was consistent among the γδ T cells, we postulated that both species could work together to regulate γδ T cell differentiation, and we thus overexpressed both upon transduction of the complete miR‐181a hairpin. We show that miR‐181a overexpression impairs human γδ T cell differentiation into TNF‐α ^+^ effectors, while also reducing the expression of NKG2D. By contrast, we show that miR‐181a seemingly did not affect differentiation into IFN‐γ producers. This highlights the complexity of action of miRNAs, including the members from the miR‐181a family, in fine tuning a restricted gene expression profile by acting on specific targets. Of note, since we found very small subsets of IL‐4^+^ (1–2%) or IL‐17A^+^ (< 1%) γδ T cells, we have not addressed the potential role of miR‐181a in type 2 or type 17 differentiation of human γδ T cells.

The concept of miR‐181a as a brake to functional γδ T cell differentiation is supported by the fact that its two main cytokine drivers, IL‐2 and IL‐15, downregulate miR‐181a(‐5p and ‐2‐3p) expression. Conversely, we found the immunosuppressive cytokine, TGF‐β, to be the main inducer of miR‐181a(‐5p and ‐2‐3p) expression in human γδ T cells. We thus propose that the upregulation of miR‐181a expression by TGF‐β may be part of a negative feedback loop that would prevent excessive inflammation in order to limit tissue damage (Li *et al*, 2017). On the other hand, established cancer settings are often rich in TGF‐β (Batlle & Massagué, 2019), and therefore, we speculate that miR‐181a upregulation may trigger γδ T cell functional exhaustion in prostate cancer, as previously reported for other miRs in human T cells in the context of breast cancer (Zhao *et al*, 2016) or non‐small‐cell lung cancer (Wan *et al*, 2018). Interestingly, previous studies demonstrated that tumor‐derived exosomal miRs can alter T cell activation or effector profile, thus highlighting potential tumor evasion mechanisms (Ye *et al*, 2014; Bland *et al*, 2018).

While miR‐181a can impact both NKG2D expression and type 1 cytokine production in differentiating γδ thymocytes, it can no longer regulate the latter process in γδ T cells isolated from the peripheral blood, which are mostly mature (Gibbons *et al*, 2009; DeBarros *et al*, 2011; Ribot *et al*, 2014), with particular effector/memory phenotypes (Dieli *et al*, 2003). This suggests that miR‐181a controls type 1 cytokine production at early stages and within a limited time window of γδ T cell differentiation and further implies that γδ T cell maturation status, which presumably associates with dynamic repertoires of mRNA targets, dictates sensitivity to miR‐181a. The inverse correlation between miR‐181a(‐5p and ‐2‐3p) expression and NKG2D levels was found in both differentiating γδ thymocytes and in circulating γδ T cells; and reduced NKG2D expression associated with high miR‐181a(‐5p and ‐2‐3p) levels in γδ T cells isolated from the blood of patients with prostate cancer. NKG2D plays a critical role in tumor surveillance, by promoting target recognition and cytotoxicity, and thus is particularly relevant to prevent tumor cell metastasis (Sivori *et al*, 2019). Furthermore, the NKG2D/NKG2DL pathway is being targeted for cancer treatment, and an improved understanding of its post‐transcriptional regulation may open new avenues for next‐generation immunotherapies.

### Concluding remarks

Our study constitutes an important addition to the molecular mechanisms that govern the acquisition of type 1 effector functions of human γδ T cells, and especially at the post‐transcriptional level which is less explored. We propose a two‐step model for a dynamic miR‐181a‐based regulation of γδ T cell differentiation, which is finely tuned within pro‐ versus anti‐inflammatory environments. First, in the thymus, elevated concentrations of TGF‐β (Jurberg *et al*, 2015) would induce high levels of miR‐181a, thus impairing Map3k2 and Notch2 expression, which ultimately would help restrain γδ T cell differentiation. Then, at peripheral inflamed sites, IL‐2 or IL‐15 signals would reduce γδ T cell expression of miR‐181a, thus releasing the functional brake to promote type 1 cytokine and NKG2D expression. This could potentially be mediated by an increase in miR‐181a mRNA targets expression, such as Map3k2 or Notch2, combined with other key regulatory mechanisms, such as epigenetic modifications. However, high miR‐181a expression levels can be induced *de novo* in peripheral γδ T cells by TGF‐β, which leads to reduced expression of NKG2D as a possible mean to prevent collateral tissue damage. We postulate that tumors take advantage of this mechanism to settle their immune evasion. Thus, we believe our work has pathophysiological implications in cancer immunity and inflammatory disease settings. Importantly, miR‐based therapies already exist or are currently under clinical trials, as it is the case for the thyroid cancer combined therapy ThyraMIR^®^/ThyGeNEXT^®^, or the Miravirsen treatment against hepatitis C (Lanford *et al*, 2009; Bonneau *et al*, 2019). This clearly highlights the potential of using miR‐based strategies in the design of new (or improved) γδ T cell‐based clinical protocols, toward their effective application in cancer treatment.

## Materials and Methods

### Patients and healthy donor samples

12 ml of peripheral blood was collected at baseline (before starting new line of therapy) from 119 patients diagnosed with stage IV breast or prostate cancer (breast cancer patients *n* = 83, 52.3 ± 11.9 years old; prostate cancer patients *n* = 36, 70.9 ± 8.5 years old) and from 47 healthy donors (female healthy donors *n* = 27, 46.3 ± 11.3 years old; male healthy donors *n* = 20, 49.0 ± 8.9 years old), followed at CHULN‐Hospital Santa Maria, Lisbon. Thymic specimens were routinely obtained during pediatric corrective cardiac surgery (from new‐born to 10‐year‐old children), performed at Hospital de Santa Cruz Carnaxide. For RT–PCR analysis of miR expression, 15 thymic samples were collected from female (*n* = 7) and male (*n* = 8) donors. For miR retroviral transduction, 11 thymic samples were collected from female (*n* = 7) and male (*n* = 6) donors. Concentrated leukocyte suspension samples (buffy‐coats) from healthy donors were collected at Instituto Português do Sangue e Transplantação. For RT–PCR analysis of miR expression, 13 buffy‐coat samples were collected from female (*n* = 5) and male (*n* = 8) donors. For miR retroviral transduction, 5 buffy‐coat samples were collected from female (*n* = 3) and male (*n* = 2) donors. For siRNA transfection experiments, 6 buffy‐coat samples were collected from female (*n* = 4) and male (*n* = 2) donors. All samples were collected with informed consent and approved by the Ethical Board of the Academic Medical Center of Lisbon CAML (CHLN/FMUL/IMM). Experiments are conformed to the principles set out in the WMA Declaration of Helsinki and the Department of Health and Human Services Belmont Report.

### Lymphocyte preparations

Blood samples were separated on a Histopaque‐1077 density gradient. Thymic samples were cut into small pieces (< 0.5 cm^2^) and smashed into a 70‐μm filter and Histopaque‐1077 (Sigma‐Aldrich) density gradient. γδ T cells were isolated by magnetic cell sorting (MACS) by positive selection, according to manufacturer's instructions (Miltenyi Biotec).

### Cell culture

MACS isolated γδ T cells were cultured at a density of 10^6^ cells/ml in round‐bottom 96‐well plates with RPMI 1640 and 2 mM L‐glutamine supplemented with 10% FBS, 1% of sodium pyruvate, 1% of HEPES, 1% of minimum essential amino acids (NEAA), and 1% of penicillin and streptomycin (Pen/Strep; all from Invitrogen Life Technologies).

For the analysis of miR‐181a expression, γδ T cells were cultured with the following cytokines: IL‐2, IL‐7, IL‐12, IL‐15, TNF‐α, IFN‐γ, IL‐4, IL‐10, and TGF‐β (all from PeproTech, 10 ng/ml). Anti‐TGF‐β blocking antibody (1D11.16.8, BioXCell, 10 μg/ml) was added when mentioned. γδ T cells from buffy‐coats or thymic biopsies were cultured for 4–6 or 11 days, respectively, and then pelleted, frozen, and stored for posterior analysis.

For retroviral transduction experiments, γδ T cells were cultured at a 5 × 10^5^ cell/ml density, round‐bottom 96‐well plates with RPMI 1640 and 2 mM l‐glutamine supplemented with 10% FBS, 1% of sodium pyruvate, 1% of HEPES, 1% of minimum essential amino acids (NEAA), and 1% of penicillin and streptomycin (Pen/Strep; all from Invitrogen Life Technologies). On the transduction days, cells were transferred into flat‐bottom 96‐well plates in a DMEM medium (Invitrogen Life Technologies) supplemented with 15% FBS, 1% of HEPES, 1% NEAA, and 1% of Pen/Strep. Indicated cytokines were added when mentioned (all from PeproTech, 10 ng/ml).

For retrovirus generation, the human embryonic kidney 293 cells with large T antigen (HEK293T) cell line (ATCC^®^ CRL‐11268™) and the NIH/3T3 cells (ATCC^®^ CRL‐1658™) were grown in T 175 flasks in DMEM supplemented with 15% FBS, 1% of HEPES, 1% of NEAA, and 1% of Pen/Strep, during 4–5 days. After reaching the required cell number for the experiment, the HEK293T cell line was cultured in 10 cm tissue culture plates (Sigma‐Aldrich TPP^®^) at a density of 5 × 10^5^ cells/ml, while the NIH/3T3 cells were cultured in flat‐bottom six‐well plates (Corning Incorporated) at a density of 7 × 10^4^ cells/ml.

For dual luciferase assays, HEK293T cell line was cultured in flat‐bottom 12‐well plates at a density of 1.5 × 10^5^ cells/ml, in DMEM supplemented with 15% FBS, 1% of HEPES, 1% of NEAA, and 1% of Pen/Strep.

For siRNA targeting, γδ T cells were cultured at a 5 × 10^5^ cell/ml density in round‐bottom 96‐well plates with RPMI 1640 and 2 mM l‐glutamine supplemented with 10% FBS, 1% of sodium pyruvate, 1% of HEPES, and 1% of minimum essential amino acids (NEAA), without antibiotics. Indicated cytokines were added when mentioned (all from PeproTech, 10 ng/ml).

All incubations were performed at 37°C in a 5% CO_2_ environment.

### Retroviral transduction of miR‐181a

The native precursor stem‐loop of the hsa‐miR‐181a was cloned into the MSCV‐IRES‐GFP vector (Addgene #9044), modified to include a PGK‐GFP‐WPRE sequence (pMIG‐PGW) as previously described (Schmolka *et al*, 2018), using human genomic DNA as a template and the following primers for hsa‐miR‐181a: forward primer, CGCCGGAATTAGATCTCCTAGTATATAGCAGATCCCCAAT and reverse primer, AACCTCGAGAGATCGACTGCTCCTTACCTTGTTGAA. Purified PCR product was then transformed in the Stellar™ Competent Cells according to the manufacturer's protocol PT5055‐2 (Clontech, 2011). The plasmid pMIG‐PGW either empty (pMIG‐PGW‐Empty) or containing the miR‐181a stem‐loop fragment was extracted from the bacteria using the GeneJET Plasmid Miniprep Kit (Thermo Fisher Scientific). The presence of the miR insert was confirmed by DNA digestion using proper restriction enzymes and analyzing the fragment lengths in an agarose gel. Validation of the results was performed by analyzing the sequences, expected and obtained, with the SnapGene^®^ Viewer Software V. 3.1.4. The viral supernatant was produced by transfecting the plasmids pMIG‐PGW (either empty or with miR‐181a), pCMV‐VSV‐G and pCL‐Eco with the Opti‐MEM^®^ (Life Technologies) and the X‐tremeGENE9 DNA Transfection Reagent (Roche) into the HEK293T TAT cells cultured in 10 cm culture plates (x‐TremeGene, 2013). Titers of infectious virus were determined by transducing 100,000 NIH/3T3 cells per well in a Nunc™ six‐well plate, with 8 μg/ml of polybrene during a 60 min centrifugation at 32°C, 974 *g*. Cells were infected at a multiplicity of infection of 2.5 or 5, which resulted in a transduction efficiency between 65–80 and 85–98%, respectively.

The pMIG‐PGW vector encodes a green fluorescent protein (GFP) that allows to assess the transduction efficiency.

For retroviral transduction of γδ T cells, the latter were cultured in 96‐well plate and stimulated overnight with plate‐bound mAbs anti‐CD3 (1 μg/ml; HIT3a; eBioscience) and anti‐CD28 (1 μg/ml; CD28.2; eBioscience) in a media supplemented with IL‐2 and IL‐7 (10 ng/m PeproTech), for 11 days. 5 × 10^5^ γδ T cells per well were transduced with pMIG‐PGW‐miR‐181a or a pMIG‐PGW‐Empty vector. The transduction was performed with 4 μg/ml of polybrene (Sigma) followed by a 45 min centrifugation at 32°C, 974 *g*. One week after the last transduction, γδ T cells were stained with mAbs for FACS analysis. Alternatively, γδ T cells were electronically sorted, based on their GFP expression, in order to assess miR‐181a transduction efficiency by RT–PCR.

### siRNA gene silencing of Map3k2 and Notch2

γδ T cells were cultured in 96‐well plate as previously described and stimulated overnight with plate‐bound mAbs anti‐CD3 (1 μg/ml; HIT3a; eBioscience) and anti‐CD28 (1 μg/ml; CD28.2; eBioscience) in a media supplemented with IL‐7 (10 ng/ml PeproTech). siRNAs were pre‐incubated with the DharmaFECT™ transfection reagent for 20 min, according to the manufacturer's protocol (Dharmacon). Cells were then transfected with either an siRNA against Map3k2, Notch2, or a negative control siRNA (nontargeting) and analyzed after 48 h. siRNAs were obtained from Dharmacon (ON‐TARGETplus Human) and used at a concentration of 500 nM.

### Flow cytometry (FACS) analysis

For intracellular cytokine staining, cells were stimulated with PMA (50 ng/ml, Sigma‐Aldrich; P‐8139) and Ionomycin (1 μg/ml, Sigma‐Aldrich; I‐0634) for 3 h at 37°C, with the addition of Brefeldin A (BFA; 10 μg/ml, Sigma‐Aldrich; B‐7651). Cells were stained for the identified cell surface markers for a period of 30 min at 4°C, fixed with Fixation/Permeabilization buffer (eBioscience) supplemented with BFA and left to incubate for 30 min at 4°C. Alternatively, to ensure the maintenance of GFP expression, retrovirally transduced γδ T cells were fixed with 4% Paraformaldehyde (Sigma‐Aldrich). Cells were then permeabilized with Permeabilization buffer (eBioscience) and resuspended in Fc block (eBioscience) for 10 min at 4°C. For intracellular cell, staining cells were incubated for 30 min at 4°C in 50 μl of Permeabilization buffer containing the indicated antibodies.

The following anti‐human fluorescently labeled mAbs were used (Table 1): anti‐Vδ1‐PE (REA173; Miltenyi Biotec), anti‐Annexin V‐APC (VAA‐33), anti‐IFN‐γ‐APC (4S.B3) (all from eBioscience), anti‐TNF‐α‐PE‐Cy7 (MAb11), anti‐NKG2D‐BV421 (1D11), anti‐Vδ2‐PerCP (B6), anti‐IL‐2‐BV510 (MQ1‐17H12), anti‐CD45RA‐BV510 (HI100), and anti‐CD27‐BV605 (O323; all from Biolegend). Live cells were identified through a staining with the LIVE/DEAD™ Fixable Near‐IR Dead Cell Stain Kit (Life technologies), for a period of 30 min at 4°C, and dead cells were electronically excluded. Transduced cells were identified by the GFP^+^ fluorescence. Cells were sorted on FACS Aria (BD Biosciences) and samples were acquired using FACS Fortessa (BD Biosciences) cell analyzer. All data were analyzed using FlowJo X 10.0.7r2 software (Tree Star).

**Table 1:** List of antibodies used for FACS analysis.

Specificity	Clone	Fluorochrome	Cat #	Origin
Vδ1	REA173	PE	130‐120‐440	Miltenyi Biotec
Annexin V	VAA‐33	APC	88‐8007‐74	eBioscience
IFN‐γ	4S.B3	APC	502512	eBioscience
TNF‐α	MAb11	PE‐Cy7	502930	Biolegend
NKG2D	1D11	BV421	320822	Biolegend
Vδ2	B6	PerCP	331410	Biolegend
IL‐2	MQ1‐17H12	BV510	500338	Biolegend
CD45RA	HI100	BV510	304142	Biolegend
CD27	O323	BV605	302830	Biolegend

### RNA isolation, cDNA production, and real‐time PCR

For miR and mRNA expression analysis, total RNA was extracted from MACS isolated γδ T cells or electronically sorted γδ T cells by using the miRNeasy Mini Kit (Qiagen) according to the manufacturer's protocol. RNA concentration and purity were determined using the NanoDrop™ 2000 spectrophotometer (Thermo Fisher Scientific).

For miR, reverse transcription was performed with miRCURY LNA^TM^ Universal RT Kit (Qiagen). For mRNA, reverse transcription from total RNA was performed with random oligonucleotides (Invitrogen) using Moloney murine leukemia virus reverse transcriptase (Promega). Total RNA was reverse‐transcribed into cDNA using the T100^®^ Thermal Cycler (Bio‐Rad), and all quantitative PCRs (qPCRs) were performed in MicroAmp^®^ Optical 384‐Well Reaction Plate (Applied Biosystems) using the RT–PCR ViiA7^TM^ system (Applied Biosystems).

For miR expression analysis, LNA^TM^ PCR primer sets (Qiagen) were used on the cDNA previously obtained and relative quantification of specific miRs to small nucleolar RNA C/D Box 44 (SNORD44) reference was carried out using SYBR on ABI ViiA7™ cycler (Applied Biosystems). For mRNA expression analysis, primer sets (Sigma) designed by Universal Probe Library Assay Design Center (Roche) were used on the cDNA previously obtained and relative quantification of specific cDNA species to endogenous references GUSB or PSMB6 was carried out using SYBR on ViiA7 cycler (Applied Biosystems).

For standard curve construction and sample absolute quantification, three unmodified oligoribonucleotides corresponding to hsa‐miR‐181a‐5p (AACAUUCAACGCUGUCGGUGAGU), hsa‐miR‐181a‐1‐3p (ACCAUCGACCGUUGAUUGUACC), and hsa‐miR‐181a‐2‐3p (ACCACUGACCGUUGACUGUACC) were synthesized (Eurofins Genomics). Different dilutions of oligoribonucleotides in RNase‐free water were prepared, and the appropriate dilution was reverse‐transcribed to cDNA in 10 μl total reaction, using the miRCURY LNA™ Universal RT Kit (Qiagen). A tenfold dilution series over six points were prepared from the cDNA and used as templates for qPCR. The standard curve was constructed by plotting *C*_q_ values against the logarithmic copy numbers of the calibrator oligoribonucleotides. The amount of an unknown sample was quantified by interpolating the *C*_q_ values in the standard curve.

For pre‐miR‐181a‐1‐3p and pre‐miR‐181a‐2‐3p expression analysis, specific primers were ordered (Eurofins Genomics) to allow for the amplification of each respective stem‐loop region, as follows: pre‐miR‐181a‐1‐3p forward primer, GAACATTCAACGCTGTCGGT, and reverse primer, GTTAGCCATAGGGTACAATCAACG; pre‐miR‐181a‐2‐3p forward primer, TATCAGGCCAGCCTTCAGAGG, and reverse primer, GTACAGTCAACGGTCAGTGGTT. Relative quantification of specific cDNA species was performed as explained above.

Data were analyzed using ViiA7™ software v1.2.1.

### Dual luciferase reporter assay

The 3′ UTR of Map3k2, Notch2, Irf4, Stat1, and a negative control (Ptbp1) were cloned into pmirGLO vector (Promega) using human genomic DNA as a template and the following primers: Map3k2 forward primer, GCTCGCTAGCCTCGAGTTGCCATACTCTTGGTTGCT, and reverse primer, CGACTCTAGACTCGAGTGTTGCACGTACCACACTGTA; Notch2 forward primer, GCTCGCTAGCCTCGAGTTCCCGGTATCCCTTGGAGT, and reverse primer, CGACTCTAGACTCGAGTTCACTTAAGGAATGTTACAAACCA; Irf4 forward primer, GCTCGCTAGCCTCGAGCGTCCAATTGACTGCCCTCT, and reverse primer, CGACTCTAGACTCGAGGGAGATCCACCTGCATCGAG; Stat1 forward primer, GCTCGCTAGCCTCGAGGACAGCAGAGCGCCTGTATT, and reverse primer, CGACTCTAGACTCGAGAACCATGCCGAATTCCCAAAG; Ptbp1 forward primer, GCTCGCTAGCCTCGAGGACCCAGGTCCAAGTTCTCG, and reverse primer, CGACTCTAGACTCGAGAAAAGCCCACGTACCTCTGG. Primers are available upon request (Sigma). In a second round of assays, mutations in the predicted target sequences for both miR‐181a‐5p and ‐2‐3p of the 3′ UTR of Map3k2 and Notch2, were introduced by gene synthesis (GeneCust Europe). Each luciferase reporter vector carrying one of the 3′ UTR sequences described above was co‐transfected with either the pMIG‐PGW‐miR‐181a or the control pMIG‐PGW‐Empty vector into the HEK293T cell line using Lipofectamine 2000 (Thermo Fisher Scientific). After 48h, firefly and Renilla luciferase activity were measured by using the Dual‐Glo Luciferase Assay System (Promega). Renilla luciferase activity served as the internal control and results were expressed as relative luciferase activity, normalized to the empty vector.

### Statistical analysis

Statistical analysis and data presentation were performed using GraphPad Prism 8 software (La Jolla). The values presented are mean ± SEM of an independent experiments. The statistical significance of differences between two populations was assessed using either the two‐tailed nonparametric Mann–Whitney *U* test or the Student's *t*‐test as indicated in the figure legends. The Pearson's correlation coefficient (*r*) was used to measure the strength of association between two variables. Correlation Strength (*r*): 0.1–0.3 weak; 0.3–0.5 moderate; 0.5–1.0 strong. Outlier analysis was applied using the Grubbs' test (Alpha = 0.01) and the ROUT test (*Q* = 1%). Statistical significance was indicated at *P < 0.05, **P < 0.01, ***P < 0.001, and ****P < 0.0001.

## Author contributions

GG designed and performed most of the experiments, analyzed the data, and contributed to the manuscript writing; SC‐P assisted in the experiments; PC and PA processed the healthy donor and patient blood samples; LC provided the healthy donors and patients samples; AQG assisted in the experimental design and provided key intellectual input; BS‐S provided critical research tools and key intellectual input, and contributed to the manuscript writing; JCR designed the study, supervised the research, and wrote the manuscript.

## Conflict of interest

The authors declare that they have no conflict of interest.

## Supporting information



Expanded View Figures PDF

Dataset EV1

Source Data for Expanded View

Source Data for Figure 1

Source Data for Figure 2

Source Data for Figure 3

Source Data for Figure 4

## Data Availability

This study includes no data deposited in external repositories.
