# Supplementary material for: MicroRNA‐181a restricts human γδ T cell differentiation by targeting Map3k2 and Notch2
Source: EMBO Rep. 2021 Nov 24;23(1):e52234. doi: 10.15252/embr.202052234 (PMC8728617; doi:10.15252/embr.202052234)
Supplement: Supplementary file 1 — Expanded View Figures PDF [file EMBR-23-e52234-s003.pdf]

Expanded View Figures

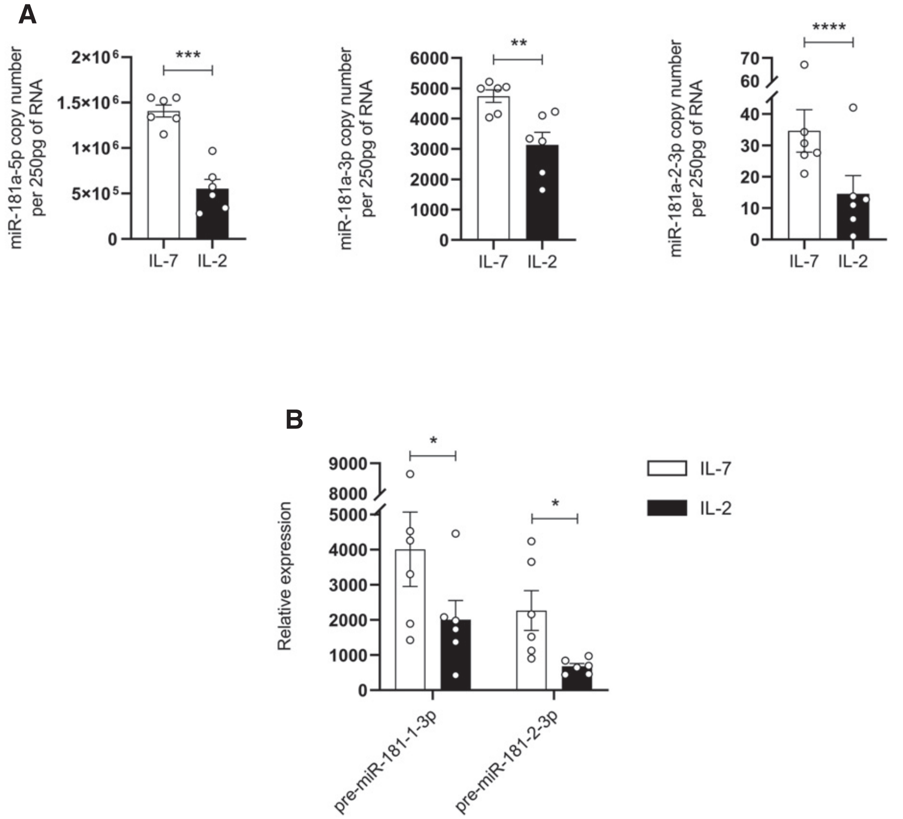

Figure EV1. miR-181a-5p and 3p strands are downregulated upon IL-2 stimulation.

A RT–PCR analysis of miR-181a-5p, miR-181a-1-3p and miR-181a-2-3p copy numbers in γδ thymocytes cultured with IL-7 versus IL-2 ($n = 6$ independent biological samples).

B RT–PCR analysis of pre-miR-181a-1-3p and pre-miR-181a-2-3p expression in γδ thymocytes cultured with IL-7 versus IL-2 ($n = 6$ independent biological samples).

Data information: Error bars represent the mean ± SEM. Statistical analysis was performed using the paired Student's *t*-test. *$P < 0.05$, **$P < 0.01$, ***$P < 0.001$, and ****$P < 0.0001$. All experiments were performed with two technical replicates.

Source data are available online for this figure.

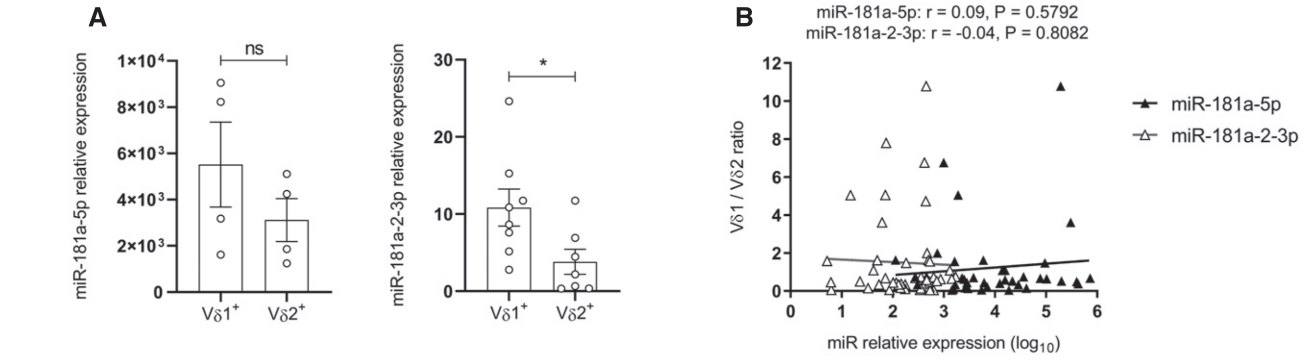

Figure EV2. miR-181a expression in Vδ1⁺ versus Vδ2⁺ γδ T cells.

A RT–PCR analysis of the expression of miR-181a(-5p and -2-3p) in freshly isolated Vδ1⁺ versus Vδ2⁺ sorted γδ PBLs ($n = 4$–8 independent biological samples).

B Correlation between Vδ1/Vδ2 ratio versus miR-181a(-5p and -2-3p) expression in freshly isolated γδ PBLs ($n = 39$–46 independent biological samples).

Data information: (A) Error bars represent the mean ± SEM. Statistical analysis was performed using the unpaired Student's *t*-test. (B) The Pearson's correlation coefficient (*r*) was used to measure the strength of association between two variables. ns, not significant. *$P < 0.05$. All experiments were performed with two technical replicates.

Source data are available online for this figure.

© 2021 The Authors

© 2021 The Authors

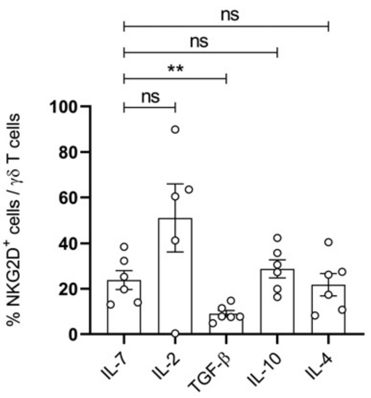

Figure EV3. TGF-β signals reduce NKG2D expression.

FACS analysis of the expression of NKGD2 in γδ T cells isolated from PBLs cultured with the indicated cytokines for 4–6 days (n = 5–6 independent biological samples, paired Student's t-test). Data represent the mean ± SEM. ns, not significant. **P < 0.01. All experiments were performed with two technical replicates.

Source data are available online for this figure.

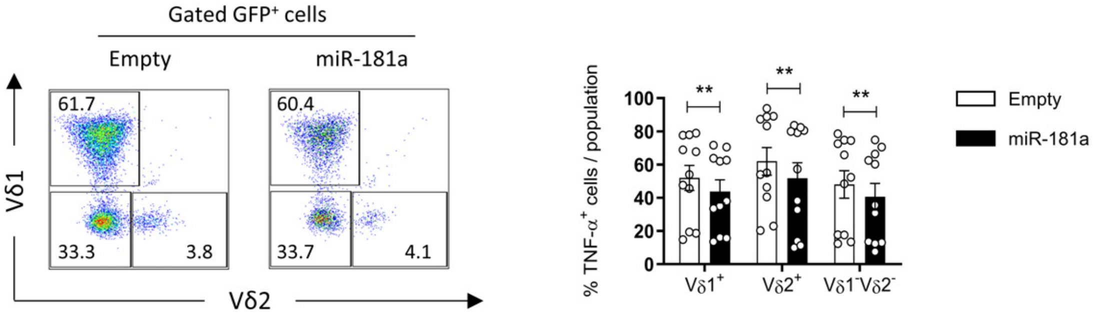

Figure EV4. miR-181a overexpression impact on TNF-α cytokine production in different γδ T cell subpopulations.

Gating strategy for the identification of the Vδ1 versus Vδ2 subpopulations in miR-181a versus empty transduced (GFP$^+$) γδ thymocytes (left panel) and TNF-α expression gated on either Vδ1$^+$, Vδ2$^+$, or Vδ1$^-$Vδ2$^-$ populations, in (GFP$^+$) miR-181a versus empty transduced γδ thymocytes, cultured with IL-7 plus IL-2 for 11 days (right panel, n = 11 independent biological samples).

Data Information: Error bars represent the mean ± SEM. Statistical analysis was performed using the paired Student's t-test. **P < 0.01. All experiments were performed with two technical replicates.
Source data are available online for this figure.

 © 2021 The Authors

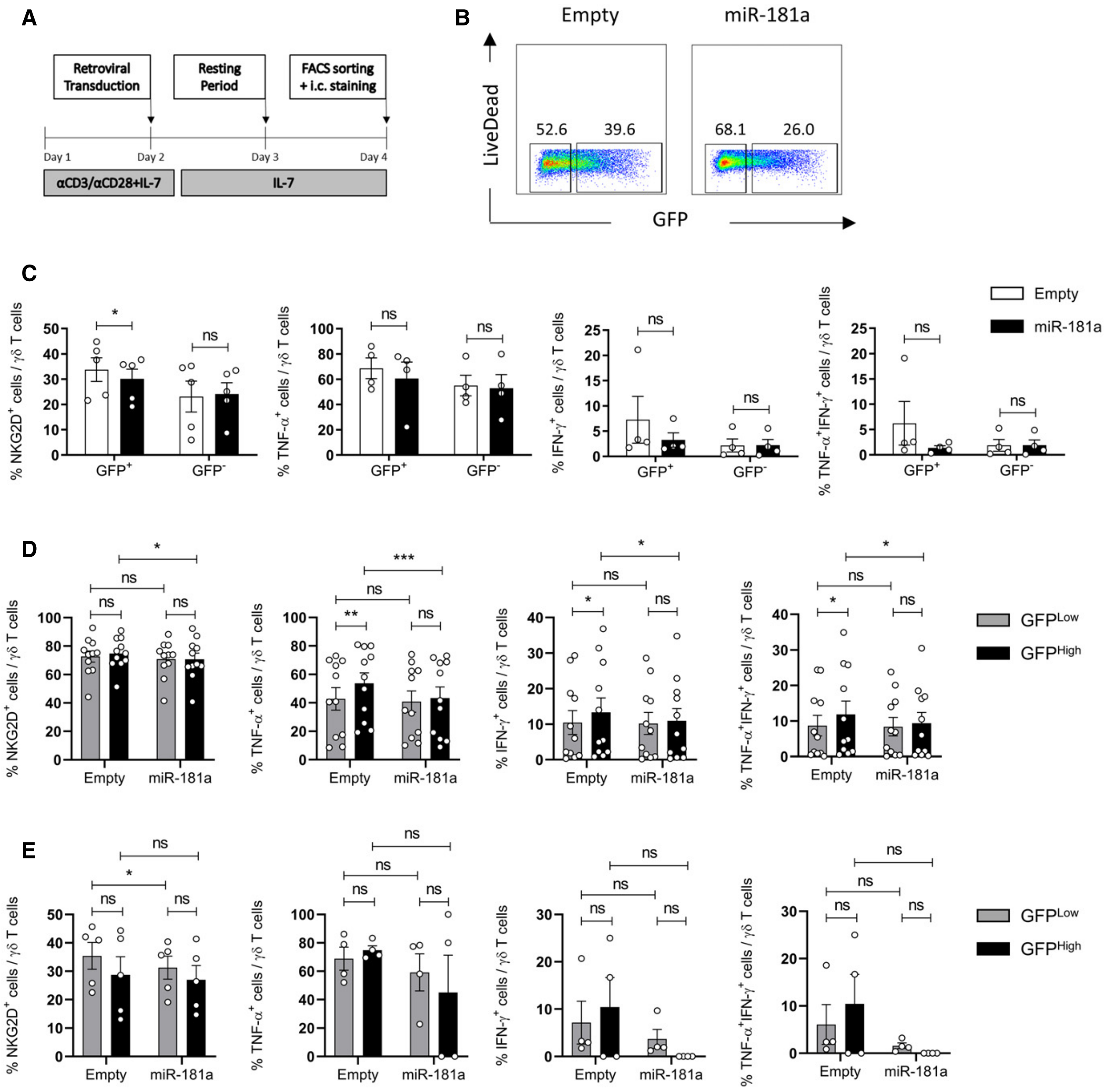

Figure EV5. Absence of dose effect of miR-181a-bearing vector transduction in γδ T PBLs and thymocytes.

A Retroviral transduction workflow for γδ PBLs.

B Gating strategy for the identification of the GFP$^+$ versus GFP$^-$ cells.

C FACS analysis of the expression of indicated surface and intracellular markers in miR-181a versus empty transduced (GFP$^+$) and untransduced (GFP$^-$) γδ PBLs, cultured with IL-7 for 4 days (n = 4–5 independent biological samples).

D, E Impact of miR-181a levels on γδ T cells isolated from (D) thymus and (E) PBLs. GFP^{Low} and GFP^{High} subsets were analyzed in miR-181a versus empty transduced γδ T cells for their expression of NKG2D, TNF-α and IFN-γ. γδ T thymocytes were cultured with IL-7 plus IL-2 for 11 days and γδ PBLs were cultured with IL-7 for 4 days (n = 11 and n = 4–5 independent biological samples, respectively).

Data Information: (C–E) Error bars represent the mean ± SEM. Statistical analysis was performed using the paired Student's t-test. ns, not significant. *P < 0.05, **P < 0.01, and ***P < 0.001. All experiments were performed with two technical replicates.
Source data are available online for this figure.

© 2021 The Authors
