# Supplementary material for: MicroRNA‐181a restricts human γδ T cell differentiation by targeting Map3k2 and Notch2
Source: EMBO Rep. 2021 Nov 24;23(1):e52234. doi: 10.15252/embr.202052234 (PMC8728617; doi:10.15252/embr.202052234)
Supplement: Supplementary file 8 [file EMBR-23-e52234-s007.pdf]

EMBO reports

MicroRNA-181a Restricts Human γδ T Cell Differentiation by Targeting Map3k2 and Notch2

Gisela Gordino, Sara Costa-Pereira, Patrícia Corredeira, Patrícia Borges Alves, Luís Costa, Anita Gomes, Bruno Silva-Santos, and Julie Ribot
DOI: 10.15252/embr.202052234

Corresponding author(s): Julie Ribot (jribot@medicina.ulisboa.pt) , Bruno Silva-Santos (bssantos@medicina.ulisboa.pt)

Review Timeline:

Submission Date:	8th Dec 20
Editorial Decision:	20th Jan 21
Revision Received:	11th May 21
Editorial Decision:	14th Jun 21
Revision Received:	16th Jun 21
Editorial Decision:	15th Jul 21
Revision Received:	30th Aug 21
Editorial Decision:	28th Sep 21
Revision Received:	14th Oct 21
Editorial Decision:	22nd Oct 21
Revision Received:	29th Oct 21
Accepted:	9th Nov 21

Editor: Achim Breiling

Transaction Report:

(Note: With the exception of the correction of typographical or spelling errors that could be a source of ambiguity, letters and reports are not edited. Depending on transfer agreements, referee reports obtained elsewhere may or may not be included in this compilation. Referee reports are anonymous unless the Referee chooses to sign their reports.)

1st Editorial Decision **20th Jan 2021**

Dear Dr. Ribot,

Thank you for the submission of your research manuscript to EMBO reports. We have now received the reports from the three referees that were asked to evaluate your study, which can be found at the end of this email.

As you will see, the referees think that these findings are of interest. However, they have several comments, concerns and suggestions, indicating that a major revision of the manuscript is necessary to allow publication of the study in EMBO reports. As the reports are below, and I think all their points need to be addressed, I will not detail them here.

Given the constructive referee comments, we would like to invite you to revise your manuscript with the understanding that all referee concerns must be addressed in the revised manuscript or in the detailed point-by-point response. Acceptance of your manuscript will depend on a positive outcome of a second round of review. It is EMBO reports policy to allow a single round of revision only and acceptance of the manuscript will therefore depend on the completeness of your responses included in the next, final version of the manuscript.

Revised manuscripts should be submitted within three months of a request for revision. We are aware that many laboratories cannot function at full efficiency during the current COVID-19/SARS-CoV-2 pandemic and we have therefore extended our 'scooping protection policy' to cover the period required for full revision. Please contact me to discuss the revision should you need additional time, and also if you see a paper with related content published elsewhere.

When submitting your revised manuscript, please also carefully review the instructions that follow below.

PLEASE NOTE THAT upon resubmission revised manuscripts are subjected to an initial quality control prior to exposition to re-review. Upon failure in the initial quality control, the manuscripts are sent back to the authors, which may lead to delays. Frequent reasons for such a failure are the lack of the data availability section (please see below) and the presence of statistics based on n=2 (the authors are then asked to present scatter plots or provide more data points).

When submitting your revised manuscript, we will require:

1) a .docx formatted version of the final manuscript text (including legends for main figures, EV figures and tables), but without the figures included. Please make sure that changes are highlighted to be clearly visible. Figure legends should be compiled at the end of the manuscript text.

2) individual production quality figure files as .eps, .tif, .jpg (one file per figure), of main figures and EV figures. Please upload these as separate, individual files upon re-submission.

The Expanded View format, which will be displayed in the main HTML of the paper in a collapsible format, has replaced the Supplementary information. You can submit up to 5 images as Expanded View. Please follow the nomenclature Figure EV1, Figure EV2 etc. The figure legend for these should be included in the main manuscript document file in a section called Expanded View Figure Legends after the main Figure Legends section. Additional Supplementary material should be supplied as a single pdf file labeled Appendix. The Appendix should have page numbers and needs to include a table of content on the first page (with page numbers) and legends for all content. Please follow the nomenclature Appendix Figure Sx, Appendix Table Sx etc. throughout the text, and also label the figures and tables according to this nomenclature.

For more details, please refer to our guide to authors:
http://www.embopress.org/page/journal/14693178/authorguide#manuscriptpreparation

See also our guide for figure preparation:
http://wol-prod-cdn.literatumonline.com/pb-assets/embo-site/EMBOPress_Figure_Guidelines_061115-1561436025777.pdf

3) a .docx formatted letter INCLUDING the reviewers' reports and your detailed point-by-point responses to their comments. As part of the EMBO Press transparent editorial process, the point-by-point response is part of the Review Process File (RPF), which will be published alongside your paper.

4) a complete author checklist, which you can download from our author guidelines (https://www.embopress.org/page/journal/14693178/authorguide). Please insert page numbers in the checklist to indicate where the requested information can be found in the manuscript. The completed author checklist will also be part of the RPF.

Please also follow our guidelines for the use of living organisms, and the respective reporting guidelines:
http://www.embopress.org/page/journal/14693178/authorguide#livingorganisms

5) that primary datasets produced in this study (e.g. RNA-seq, ChIP-seq and array data) are deposited in an appropriate public

database. If no primary datasets have been deposited, please also state this in the respective section (e.g. 'No primary datasets have been generated and deposited'), see below.

See also: http://embor.embopress.org/authorguide#datadeposition

Please remember to provide a reviewer password if the datasets are not yet public.

The accession numbers and database should be listed in a formal "Data Availability " section (placed after Materials & Methods) that follows the model below. This is now mandatory (like the COI statement). Please note that the Data Availability Section is restricted to new primary data that are part of this study.

Data availability

The datasets produced in this study are available in the following databases:

- RNA-Seq data: Gene Expression Omnibus GSE46843 (https://www.ncbi.nlm.nih.gov/geo/query/acc.cgi?acc=GSE46843)
- [data type]: [name of the resource] [accession number/identifier/doi] ([URL or identifiers.org/DATABASE:ACCESSION])

*** Note - All links should resolve to a page where the data can be accessed. ***

Moreover, I have these editorial requests:

6) We strongly encourage the publication of original source data with the aim of making primary data more accessible and transparent to the reader. The source data will be published in a separate source data file online along with the accepted manuscript and will be linked to the relevant figure. If you would like to use this opportunity, please submit the source data (for example scans of entire gels or blots, data points of graphs in an excel sheet, additional images, etc.) of your key experiments together with the revised manuscript. If you want to provide source data, please include size markers for scans of entire gels, label the scans with figure and panel number, and send one PDF file per figure.

7) Our journal encourages inclusion of *data citations in the reference list* to directly cite datasets that were re-used and obtained from public databases. Data citations in the article text are distinct from normal bibliographical citations and should directly link to the database records from which the data can be accessed. In the main text, data citations are formatted as follows: "Data ref: Smith et al, 2001" or "Data ref: NCBI Sequence Read Archive PRJNA342805, 2017". In the Reference list, data citations must be labeled with "[DATASET]". A data reference must provide the database name, accession number/identifiers and a resolvable link to the landing page from which the data can be accessed at the end of the reference. Further instructions are available at: http://www.embopress.org/page/journal/14693178/authorguide#referencesformat

8) Regarding data quantification and statistics, can you please specify, where applicable, the number "n" for how many independent experiments (biological replicates) were performed, the bars and error bars (e.g. SEM, SD) and the test used to calculate p-values in the respective figure legends. Please provide statistical testing where applicable, and also add a paragraph detailing this to the methods section. See:
http://www.embopress.org/page/journal/14693178/authorguide#statisticalanalysis

9) Please also note our new reference format:
http://www.embopress.org/page/journal/14693178/authorguide#referencesformat

Finally, please note that all corresponding authors are required to supply an ORCID ID for their name upon submission of a revised manuscript. Please find instructions on how to link the ORCID ID to the account in our manuscript tracking system in our Author guidelines: http://www.embopress.org/page/journal/14693178/authorguide#authorshipguidelines

I look forward to seeing a revised version of your manuscript when it is ready. Please let me know if you have questions or comments regarding the revision.

Yours sincerely

Achim Breiling
Editor
EMBO Reports

Referee #1:

In their manuscript Gordino and colleagues characterize the function of miRNA miR-181a in human gdT cells. Whereas the role of this miRNA in murine T cells is well characterized, its function in human T cells remains largely elusive. In particular human

gdT cells have some characteristics not shared by their murine counterparts, including distinct transcriptional requirements during development and the existence of subsets that constitute promising targets in tumor therapy. This manuscript adds to the difference between human and murine gdT cells in defining a role of miR-181a in differentiation. The authors describe elevated expression of miR-181a in gdT cells derived from prostate cancer patients (but not breast cancer patients). Moreover, they demonstrate a differentiation-dependent decline in miR-181a expression in thymic gdT cells upon stimulation with IL-2 and IL-15. Conversely, miR-181a expression was elevated upon stimulation by TGF-b. Using retrovirus-mediated overexpression they show that ectopic expression of miR-181a limits differentiation of gdT cells, as indicated by reduced frequencies of TNFa+, IL-2+, and NKG2D+ cells. Finally, employing ectopic expression and luciferase assays, they provide evidence for Notch2 and Map3k2 as potential targets of miR-181a-2-3p. The information presented in this study is novel and may add to our understanding of gdT cell differentiation in the context of human cancer. However, the largely descriptive nature, lack of mechanistic insight as well as some conceptual and technical flaws limit the enthusiasm for this manuscript.

Fig. 2 shows that reduced miR-181a expression is directly linked to differentiation of thymic gdT cells, but not gdT cells derived from PB. How do the authors connect this comparative lack of responsiveness to their findings from Fig. 1?

Some observed effects appear to be not very generalizable: Elevated miR-181a levels are only observed in PC gdT cells, but not BC gdT cells. IL-10 has a significant effect, but in terms of effect size there is no difference to IL-4, TNFa or IFNg. Ectopic expression of miR-181a limits differentiation in most aspects, but selectively does not hamper IFNg production. For IL-2 there is a reduction, but it appears that empty vector has a larger (opposing) effect than ectopic expression of miR-181a.

The consequences of ectopic expression require a more extensive analysis to permit refined conclusions: The complete system contains IL-2 as differentiation factor early on. In light of the findings of Fig. 2D vs. Fig. 2E this might hamper the outcome of this experiment. It would be more informative to attempt transduction in the presence of IL-7 only or at least remove IL-2 after sorting. The consequence of limited survival vs. limited differentiation requires attention as well.

Identification of targets Map2k3 and Notch2 is based on ectopic expression of miR-181a followed by mRNA expression analysis in gdT cells and luciferase assays in HEK293T cells. There is no evidence that the changes of expression of these putative targets are causative for the observed block in differentiation or that MAPK and/or Notch signaling are even affected by these changes. The indicated base pairings in Fig. 4A are not aligned with the actual base pairings.

It is widely assumed that for miR-181a the 5p strand is much more likely to be incorporated into the RISC than the 3p strand (cf. also the cumulative RNAseq data in miRbase). Can the authors exclude a substantial contribution of 5p? Along these lines: Throughout Figs. 1 and 2, the qPCR assays appear to provide a broad range of relative expression values e.g., ~500 in ctrl M in Fig. 1D vs. barely detectable in Fig. 2B (were these male or female samples?) or ~50 in IL-7 control in Fig. 2A vs. ~5000 (a 100-fold difference!) in Fig. 2B.

The authors suggest a role for miR-181a-2 in this setting. This is interesting, because in mouse, miR-181a-2 expression is largely restricted to germ cells, whereas the majority of miR-181a in lymphocytes stems from miR-181a-1. The authors should more carefully explore a potential contribution of miR-181a-1.

Referee #2:

In this interesting study by Gordino and colleagues the authors show that miR-181a expression is up-regulated in γδ T cells from the peripheral blood of a subset of metastatic breast and prostate cancer patients, compared with γδ T cells from gender-matched healthy controls.
The authors then switch to freshly isolated human γδ thymocytes and in vitro differentiation cultures of these cells. Here, expression of miR-181a is high in immature γδ thymocytes, becomes downregulated upon type 1 effector differentiation and correlates negatively with the expression of hallmark transcripts of type 1 γδ T cells (IFN-γ, TNF-α, and NKG2D) that are involved in cancer surveillance. A negative correlation between miR-181a and NKG2D but not IFN-γ and TNF-α can be found in γδ T cells isolated from the peripheral blood. In order to elucidate the mechanism, the authors perform gain-of-function studies in immature γδ thymocytes and find functional effector differentiation is impaired in miR-181a overexpressing cells. By focusing on the Notch and MAPKinase signaling pathways, previously shown to be important for functional differentiation of mature T lymphocytes, the authors identify Map3k2 and Notch2 as miR-181a targets.
The role of miR-181a has been extensively studied in αβ T cells with miR-181 having pleiotropic functions in T cell signaling and development. In a recent publication (Amado et al, 2020) the authors have shown a role for miR-181a in regulating IFN-γ-production in effector CD8 T cells via Id2. miR-181a has no effect on murine γδ T cell development or γδ1/γδ17 differentiation (Sandrock et al, 2015) but has not been studied in human γδ T cells before.
Taken together, the manuscript is the first to investigate the role of miR-181a in human γδ T cells and implicates its role in acquisition of type1 effector function potentially via regulation of Map3k2 and Notch2 transcripts. The link between this exciting novel in vitro molecular mechanism in γδ thymocytes and the clinical observation in metastatic cancer patients is however less clear and it remains to be seen whether dis-regulated miR-181a expression could lead to an impaired type 1 γδT cell surveillance in prostate and breast cancer.

Major points:

1. The authors show that γδ T cell counts are lower in the blood of metastatic cancer patients. This is likely due to the dose and type of anti-cancer therapy these patients have been receiving at the time of sampling. The authors have to discuss this and investigate whether the extend of γδ T cell reduction correlates with treatment intensity. It is further unknown to which extend, if at all, theyγδ T cell compartment in the peripheral blood is representative of the γδ T cell compartment in the tumour. If possible, it would be good to show increased miR-181a expression in γδ T cells from the TME of resected tumours from these patients.
2. Only γδ T cells from a subset of metastatic cancer patients showed increased miR-181a expression. Is miR-181a expression correlated to TCRγ and TCRδ subsets? Or cytokine profile (steady state and after stim)? Or NKG2D expression?
3. A major disconnect in the manuscript represents the author's observation of miR-181a expression in mature peripheral γδ T cells in prostate and breast cancer patients and the author's proposed molecular mechanism that they characterized using immature γδ thymocytes cultures in vitro. In γδ thymocytes expression of miR-181 is negatively correlated to type 1 effector phenotypes (NKG2D expression and production of IFN-γ, TNF-α upon stimulation) (Fig. 2D). By contrast, only NKG2D expression but not secretion of type 1 cytokines is inversely correlated with miR-181a expression in peripheral γδT cells isolated from blood (Fig. 2E). These results suggest that miR-181a may not affect type 1 effector functions of differentiated mature γδT cells in the periphery. Thus, the authors should explain clearer how the miR-181a expression is dis-regulated in γδT cells in the peripheral blood of cancer patients. Moreover, the authors show in Fig. 3 only γδ thymocytes but not mature peripheral γδ T cells that overexpression of miR-181a in vitro compromise their type 1 effector functions. The authors should provide results showing the effect of miR-181a in vitro with peripheral γδ T cells from healthy individuals and/or cancer patients to demonstrate the pathological relevance of miR-181a in modulating the type 1 γδ effector functions.
4. The chosen effectors downstream of miR-181a are finely picked. Have the authors analysed a wider range of potential targets? Have they checked Id2 levels in miR-181a overexpressing cells? Why does miR-181a overexpression lead to increased apoptosis? Can miR-181a-insensitive Map3k2 and Notch2 mutants rescue the phenotype?

Minor points:

1. Fig. 1: Please indicate which cancer patients are age-matched with which healthy controls.
2. Have the authors investigated the expression of miR-181a in γδ T cells at earlier stages of cancer progression?
3. Fig. 1: Please use the same y-axis for Fig. 1D and Fig. 1E. In the current version the figure is misleading and it is not clear whether for the majority of samples the statement in line 99-100 ("miR-181a expression was substantially higher in γδT cells when compared to their αβ T cell counterparts.")
4. Lines 136-139: "Interestingly, IFN-γ and TNF-α expression did not correlate with miR-181a expression in ex vivo peripheral blood γδ T cells (Fig 2E), suggesting a role for miR-181a during the differentiation process, after which cytokine production becomes constitutive in mature cells." Could the authors clarify at which stage of the differentiation process, the miR-181a expression is affecting immature γδ thymocytes and mature peripheral γδ T cells differently? Immature γδ T thymocytes are affected before lineage commitment and mature peripheral γδ T cells are affected only after the lineage is determined and constitutively secreting type 1 cytokines? Again, in this case, would the molecular mechanisms identified using γδ thymocytes be applicable to mature peripheral γδ T cells in cancer patients?
5. Fig. 2B: Peripheral γδ T cells seem to express miR-181a in a much wider range compared to γδ thymocytes. Could it be due to the composition of different γδ T cell subsets in different individuals? (see major point 2)
6. Fig. 2D: Do the authors find positive correlations between immature γδ T cell markers and miR-181a?
7. The authors should investigate whether modulation of miR-181a expression affects the differentiation of γδ T cells (thymocytes and/or peripheral cells) toward IL-17 lineages and their effector functions (eg. IL-17 secretion) upon in vitro stimulation.
8. Please indicate in the methods how ecotropic pMIG-based retroviruses were made to infect human cells.
9. Fig. 3: Transduction of γδ T cells with pMIG does not result in discret GFP positive and negative subsets but rather a smear. Due to the IRES levels of GFP expression should correlate with levels of miR-181a expression. Have the authors gated on GFP high/medium/low cells overexpressing miR-181a and observed a dose response?
10. In all qRTPCR experiments multiple housekeeping genes should be used to validate the results. Please indicate.

Referee #3:

In this manuscript, Gordino and co-authors report that miR-181a inhibits functional maturation of human γδ T-cells via effects on Map3k2 and Notch2. Given the potent anti-tumor effector functions of the γδ T-cell lineage, these data aim to inform ongoing efforts to manipulate γδ T-cells for cancer treatment (currently a hot topic in immunotherapy). Consistent with this concept, the authors were able to identify that blood γδ T-cells from patients with metastatic prostate cancer display upregulation of miR-181a in parallel with reduced expression of the cytotoxicity mediator NKG2D.

Differential expression levels of miR-181a between immature thymocytes and derivative peripheral T-cell populations has previously been identified in murine models (which the authors have referenced appropriately in the discussion). There remains substantial novelty in identifying if/how this molecule can modulate the γδ T-cell compartment in human blood and tissues, which

is a major focus of current research in oncoimmunology. The data presented in support of the authors' claims are generally of good quality and presented in a clear, concise fashion throughout. Greater discussion of the possible interpretations of these findings would be beneficial.

It would have been valuable to determine if miR-181a only impacts on the Th1 functions of activated γδ T-cells, but the authors have not assessed alternative fates in their manuscript (previous work in murine models has also observed inhibition of conventional Th2 effector cells for example). Did the authors also attempt to establish whether γδ T-cell acquisition of Th17 effector functions can also be impaired by miR-181a? This would be interesting to know, although I suspect quite difficult to test given the challenges of inducing this γδ T-cell profile in a human system. For the short report format being used here, these data would probably qualify as 'nice to have' rather than critical to the authors' conclusions.

A significant omission is the lack of discrimination between Vδ1 and Vδ2 subsets in the authors' data (although they have clearly assessed these populations separately judging by the use of subset-specific antibodies in the methods section). The functional differences between these populations are significant enough that I would not anticipate uniform results for each across the various assay types employed in this report. In several of the graphs displayed, there appear to be both cytokine high and low expressing populations of γδ T-cells present, so it is possible that miR-181a does not impact the biology of both subsets equally. Currently, it is not possible to assess this because only global results for the total γδ T-cell pool are provided. If technical limitations necessitated the use of bulk γδ T-cell preparations in places, then this should be acknowledged / explained in the text. At present, the Vδ2 population is suddenly mentioned in line 164 of the manuscript without any further explanation, which will be difficult for a non-specialist audience to understand.

The influence of miR-181a appears to occur during γδ thymocyte differentiation rather than by impacting on the effector functions of mature cells. Consistent with this, the authors observed that IFNγ and TNFα expression by peripheral blood γδ T-cells did not correlate with miR-181a expression. This being the case, how do the authors propose that miR-181a inhibits the blood γδ T-cell pool of >50yo cancer patients? (who presumably have minimal thymic output by this age). Couldn't the reduced blood frequency of γδ T-cells in these individuals simply be a function of age? I suppose it is possible that γδ thymic emigrants might display increased miR-181a levels / defective Th1 function for a long period prior to oncogenesis. Alternatively, perhaps this miRNA species can indeed be induced in the periphery / is able to modify the activity of mature γδ T-cells in a cancer setting? Whatever their interpretation, it would be useful for the authors to discuss their findings as part of a broader framework that helps readers to understand the possible implications (assuming that space limitations don't prevent this).

Minor points
In Fig 3E, overexpression of miR-181a looks to have minimal impact on cytokine expression (TNF / IFNγ), while effects on other readouts appear extremely variable (NKG2D). It would be very useful to see how these results segregate between Vδ1 / Vδ2 lineages.

IL-7 is included for some experiments (γδ thymocyte transduction) but later omitted when molecular targets are being identified (Map3k2). Would be useful to explain the reasoning.

Fig. 2A and C: Would be useful to see IL-2 and IL-15 data on the same graph (currently only displayed in isolation - how do these compare?)

Line 106/107: Should specify pediatric biopsies *of thymus*.

Line 180-186: The rationale for focusing on Notch and Mapk signaling pathways could be explained in more detail. What were the specific criteria applied / how were other things excluded?

Line 189: Specify the negative control (PTBP1)

Line 289: Should read "diagnosed with stage IV breast *or* prostate cancer".

Line 300: Should explain the "tissue dispersion" method in more detail.

Line 667: Add symbols for TNF-a and IFN-g.

Point-by-point reply to the Reviewers' comments (Gordino, et al. EMBOR-2020-52234V1)

We thank the Editor and the Reviewers for their constructive criticisms, which prompted a series of additional experiments and clarifications that significantly improved the quality of our revised manuscript. We provide below a point-by-point reply to the concerns that were previously raised. Changes in the revised manuscript are highlighted in yellow.

Referee #1 comments:

In their manuscript Gordino and colleagues characterize the function of miRNA miR-181a in human gdT cells. Whereas the role of this miRNA in murine T cells is well characterized, its function in human T cells remains largely elusive. In particular human gdT cells have some characteristics not shared by their murine counterparts, including distinct transcriptional requirements during development and the existence of subsets that constitute promising targets in tumor therapy. This manuscript adds to the difference between human and murine gdT cells in defining a role of miR-181a in differentiation. The authors describe elevated expression of miR-181a in gdT cells derived from prostate cancer patients (but not breast cancer patients). Moreover, they demonstrate a differentiation-dependent decline in miR-181a expression in thymic gdT cells upon stimulation with IL-2 and IL-15. Conversely, miR-181a expression was elevated upon stimulation by TGF-b. Using retrovirus-mediated overexpression they show that ectopic expression of miR-181a limits differentiation of gdT cells, as indicated by reduced frequencies of TNFa+, IL-2+, and NKG2D+ cells. Finally, employing ectopic expression and luciferase assays, they provide evidence for Notch2 and Map3k2 as potential targets of miR-181a-2-3p. The information presented in this study is novel and may add to our understanding of gdT cell differentiation in the context of human cancer. However, the largely descriptive nature, lack of mechanistic insight as well as some conceptual and technical flaws limit the enthusiasm for this manuscript.

Fig. 2 shows that reduced miR-181a expression is directly linked to differentiation of thymic gdT cells, but not gdT cells derived from PB. How do the authors connect this comparative lack of responsiveness to their findings from Fig. 1?

We thank the Reviewer for raising this issue and giving us the opportunity to clarify this important point linked to the dynamics of γδ T cell differentiation and maturation status.
We propose that the γδ T cell maturation status, which presumably associates with dynamic repertoires of mRNA targets, dictates sensitivity to miR181a. Contrary to their mature circulating counterparts, γδ T cells isolated from thymic biopsies are functionally immature and their type 1 differentiation (induced *in vitro* upon IL-2 signaling) is fully permissive to miR-181a regulation (Fig 3E). Our results in γδ thymocyte cultures indeed suggest that miR-181a controls γδ T cell expression of type 1 cytokines and NKG2D in an early time window.
We further show that high miR-181a expression associates with low NKG2D expression in circulating γδ T cells from patients with prostate cancer (Fig 1). Consistently, we also observe an

inverse correlation between miR-181a and NKG2D expression levels in circulating γδ T cells from healthy donors (Fig 2F). However, whereas miR-181a expression inversely correlated with the expression of IFN-γ and TNF-α in γδ thymocytes (Fig 2E), it did not correlate nor associate with these hallmarks in circulating γδ T cells, whether they were isolated from healthy donors (Figure 2F) or from cancer patients (Fig EV1).
Thus, we propose a two-step model for a dynamic miR181a based-regulation of γδ T cell differentiation, which is finely tuned by pro- versus anti-inflammatory environments. First, in the thymus, elevated concentrations of TGF-β (Jurberg *et al*, 2015) maintain γδ T cell in an immature state and fully permissive to miR181a action. Then, at inflamed peripheral sites, IL-2 or IL-15 signals would reduce γδ T cell expression of miR181a and promote their expression of type 1 cytokine and of NKG2D. Importantly, peripheral γδ T cells remain partially sensitive to miR181a, whose expression can be *de novo* induced by TGF- β, thus reducing γδ T cell expression of NKG2D in order to prevent collateral tissue damage. We postulate that tumors may have taken advantage of this mechanism to settle their immune evasion. Thus, we believe our work has pathophysiological implications in cancer immunity and inflammatory disease settings. Altogether, these considerations are now discussed pages 9-12 of our revised manuscript.

Some observed effects appear to be not very generalizable: Elevated miR-181a levels are only observed in PC gdT cells, but not BC gdT cells. IL-10 has a significant effect, but in terms of effect size there is no difference to IL-4, TNFa or IFNg. Ectopic expression of miR-181a limits differentiation in most aspects, but selectively does not hamper IFNg production. For IL-2 there is a reduction, but it appears that empty vector has a larger (opposing) effect than ectopic expression of miR-181a.

We agree that not all the parameters assessed in our study are generalizable, but this is expected from the fine tuning of specific target gene expression typically ascribed to microRNAs. Thus, the impact of a given miRNA is generally mild and selective, which may explain why ectopic expression of miR-181a limits certain features of γδ T cell type 1 differentiation (such as TNF-α expression), while not affecting others (namely IFN- γ expression). Moreover, as highlighted in our response to the first comment above, miR-181a action on γδ T cell differentiation seemingly depends on the cell's maturation status (likely due to the dynamic interplay between miRNA and mRNA repertoires).
We took on board the Reviewer's comments on the IL-10 effect on miR-181a expression; and on the caveat of the empty vector control on the IL-2 expression data, and thus removed the respective reference and/ or data in the revised manuscript.
Regarding the Reviewer's point on miR-181a expression in γδ T cells from patients with breast versus prostate cancer, we were able to exclude a potential sex bias, by showing a similar expression of miR-181a in male and female circulating γδ T cells from the control samples (Figure 1D, no significant differences between the CTRL M and CTRL F groups).

Furthermore, the nature of the treatment received also does not seem to influence miR-181a expression in circulating γδ T cells, although we would need to enroll more patients in the radiotherapy and combination groups to completely rule out this possibility (Figure R1).

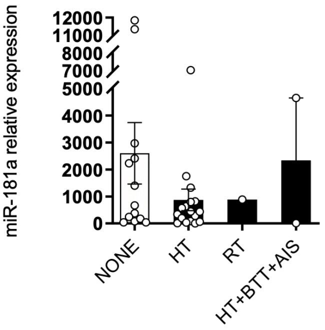

<u>Figure R1:</u> Influence of cancer treatment on miR-181a expression in freshly isolated γδ PBLs from prostate cancer patients who received: no treatment in the previous 6 months before blood collection (NONE); Hormone Therapy (HT); Radiotherapy (RT); Bone-Targeted Therapy (BTT); or Anti-Inflammatory Steroid (AIS).

The consequences of ectopic expression require a more extensive analysis to permit refined conclusions: The complete system contains IL-2 as differentiation factor early on. In light of the findings of Fig. 2D vs. Fig. 2E this might hamper the outcome of this experiment. It would be more informative to attempt transduction in the presence of IL-7 only or at least remove IL-2 after sorting. The consequence of limited survival vs. limited differentiation requires attention as well.

We have previously demonstrated that naive γδ T cells isolated from thymic biopsies acquire type 1 / cytotoxic functions in response to IL-2, but not IL-7, signals (Ribot, JI 2014). In other words, removing IL-2 from the system would prevent the induction of γδ thymocyte differentiation, and thus from uncovering the impact of miR-181a on this process.
Furthermore, although IL-7 is known to promote γδ T cell survival, we observed that in its presence alone (i.e., in the absence of IL-2), γδ T cells are more sensitive to manipulation, as revealed by the proportion of apoptotic cells (80% of Annexin V^+ in GFP^+ cells *versus* 40% in GFP^-) (Figure R2). Naturally, all dead cells were electronically excluded based on a livedead staining (as now clarified in the Material and Method section of our revised manuscript).

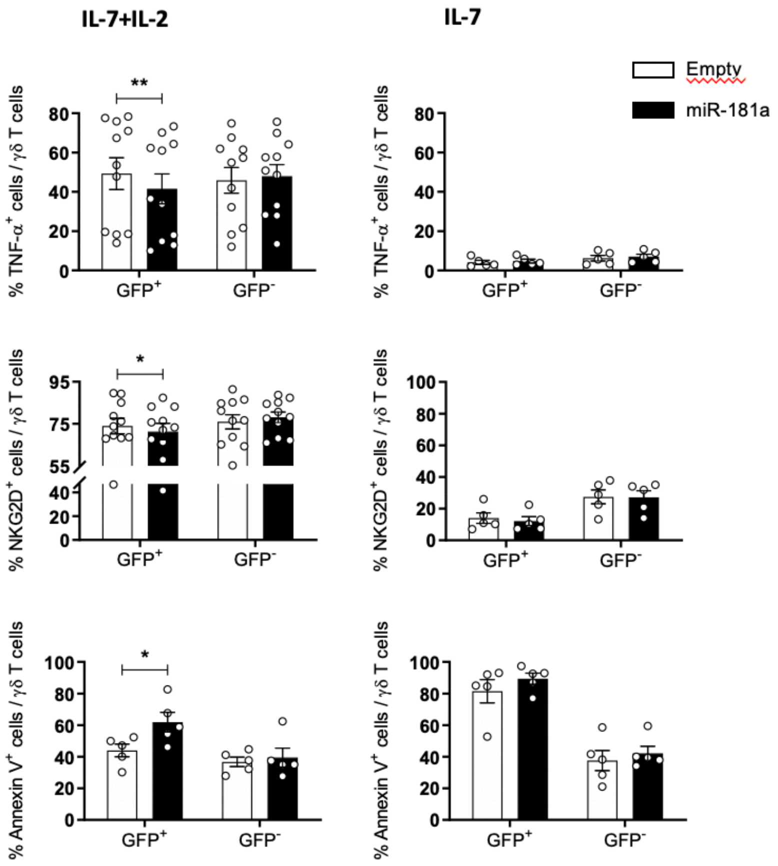

Figure R2: **FACS analysis of the expression of indicated surface and intracellular markers in miR-181a *versus* empty transduced (GFP^+) and untransduced (GFP^-) γδ thymocytes, cultured with IL-7+IL-2 (left panel) *versus* IL-7 only (right panel). Paired Student's t-test. *$P < 0.05$ and **$P < 0.01$.**

This notwithstanding, we have now directly addressed the Reviewer's suggestion to remove IL-2 from our miR-181a overexpression experimental design, using (mature) circulating γδ T cells, that were isolated from the peripheral blood of healthy donors and transduced in the presence of IL-7 only. Consistent with the correlations reported in Figure 2F, we observed that miR-181a overexpression in circulating γδ T cells reduced their expression of NKG2D without affecting their expression of IFN-γ or TNF-α (please refer to our new Figure EV6).

Identification of targets Map2k3 and Notch2 is based on ectopic expression of miR-181a followed by mRNA expression analysis in gdT cells and luciferase assays in HEK293T cells. There is no evidence that the changes of expression of these putative targets are causative for the observed block in differentiation or that MAPK and/or Notch signaling are even affected by these changes.

We thank the Reviewer for raising this key point. To support our identification of Map2k3 and Notch2 as miR-181a targets, we have analyzed the expression of genes linked to MAPK (namely Atf2, c-fos and p38) and NOTCH (namely Hes1 and Rbpj) signaling pathways. We observed that γδ T cells transduced with miR-181a showed a reduction of the expression of these genes, when

compared to control samples. These results were added to the revised version of our manuscript as new Fig 4B, which is mentioned on lines 218-221.

Most importantly, we further investigated whether siRNA targeting Map3k2 and Notch2 could phenocopy the effect of miR-181a overexpression. As we could not have access to new thymic biopsies during the pandemic period, we performed these experiments using γδ T cells from healthy donor peripheral blood. We observed a modest but significant decrease in NKG2D (but not TNF-α) expression upon Map3k2 and/or Notch2 siRNA treatment, which is consistent with the impact of miR-181a overexpression on circulating γδ T cells (Figure EV6), and was added to our revised manuscript as new Fig 4D and discussed on lines 240-242.

The indicated base pairings in Fig. 4A are not aligned with the actual base pairings.

We apologize for this error and have corrected Figure 4A accordingly.

It is widely assumed that for miR-181a the 5p strand is much more likely to be incorporated into the RISC than the 3p strand (cf. also the cumulative RNAseq data in miRbase). Can the authors exclude a substantial contribution of 5p?

Our qPCR primers and the strand loaded in the plasmid designed for our retroviral transduction and luciferase assays is specific for miR-181a-2-3p, so we did not assess the contribution from the 5p strand in our experiments. As mentioned below, we do not exclude a potential impact miR-181a-5p in γδ T cell differentiation, as its expression was also decreased in IL-2 (compared to IL-7) cultured γδ thymocytes (please see Fig R3 below).

Along these lines: Throughout Figs. 1 and 2, the qPCR assays appear to provide a broad range of relative expression values e.g., ~500 in ctrl M in Fig. 1D vs. barely detectable in Fig. 2B (were these male or female samples?) or ~50 in IL-7 control in Fig. 2A vs. ~5000 (a 100-fold difference!) in Fig. 2B.

We thank the Reviewer for this remark. The samples used in Figure 2 were from both males and females, which we pooled since there were no significant differences in miR-181a expression; these informations has been added to the Material and Methods section.

On the other hand, variations in miR-181a range of expression between γδ T cells from patient and control blood samples (in Figure 1) versus buffy coat samples (Figure 2) could be explained by differences in sample collection and conservation until being processed. Blood from patients and healthy donors are collected in EDTA coated tubes while buffy coats are kept in specific CompoFlow bag, which are supplemented with citric acid monohydrate, sodium citrate dihydrate, glucose monohydrate and sodium dihydrogen phosphate dihydrate. Regarding γδ thymocytes, the variations observed between Fig. 2A and Fig. 2B are likely due to the impact of cell culture, as they are respectively from 11 day cultures versus freshly isolated from thymic biopsies. Importantly, and given such intrinsic limitations, no comparison of relative miR-181a expression levels across different experimental conditions is being made in our manuscript.

The authors suggest a role for miR-181a-2 in this setting. This is interesting, because in mouse, miR-181a-2 expression is largely restricted to germ cells, whereas the majority of miR-181a in lymphocytes stems from miR-181a-1. The authors should more carefully explore a potential contribution of miR-181a-1.

We thank the Reviewer for this comment. We acknowledge that γδ T cell differentiation could be potentially influenced by other microRNAs, namely by the related miR-181a-1, which expression was also decreased in IL-2 (compared to IL-7) cultured γδ thymocytes (Fig R3). However, given the amount of work needed to decisively address this hypothesis, we kindly suggest to follow-up this data in future studies.

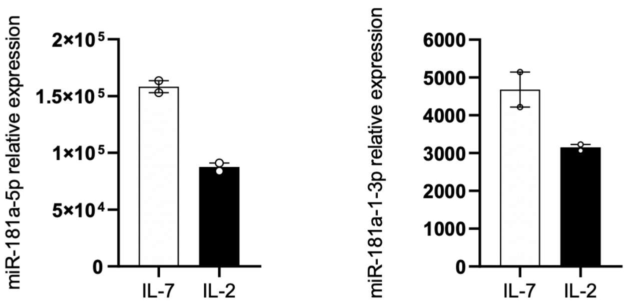

<u>Figure R3:</u> miR-181a-5p and miR-181a-1-3p expression in thymic γδ T cells cultured for 11 days in the presence of IL-7 or IL-2.

Referee #2 comments:

In this interesting study by Gordino and colleagues the authors show that miR-181a expression is up-regulated in γδ T cells from the peripheral blood of a subset of metastatic breast and prostate cancer patients, compared with γδ T cells from gender-matched healthy controls. The authors then switch to freshly isolated human γδ thymocytes and in vitro differentiation cultures of these cells. Here, expression of miR-181a is high in immature γδ thymocytes, becomes downregulated upon type 1 effector differentiation and correlates negatively with the expression of hallmark transcripts of type 1 γδ T cells (IFN-γ, TNF-α, and NKG2D) that are involved in cancer surveillance. A negative correlation between miR-181a and NKG2D but not IFN-γ and TNF-α can be found in γδ T cells isolated from the peripheral blood. In order to elucidate the mechanism, the authors perform gain-of-function studies in immature γδ thymocytes and find functional effector differentiation is impaired in miR-181a overexpressing cells. By focusing on the Notch and MAPKinase signaling pathways, previously shown to be important for functional differentiation of mature T lymphocytes, the authors identify Map3k2 and Notch2 as miR-181a targets.

The role of miR-181a has been extensively studied in αβ T cells with miR-181 having pleiotropic functions in T cell signaling and development. In a recent publication (Amado et al, 2020) the authors have shown a role for miR-181a in regulating IFN-γ-production in effector CD8 T cells via Id2. miR-181a has no effect on murine γδ T cell development or γδ1/γδ17 differentiation (Sandrock et al, 2015) but has not been studied in human γδ T cells before.

Taken together, the manuscript is the first to investigate the role of miR-181a in human γδ T cells and implicates its role in acquisition of type1 effector function potentially via regulation of Map3k2 and Notch2 transcripts. The link between this exciting novel in vitro molecular mechanism in γδ thymocytes and the clinical observation in metastatic cancer patients is however less clear and it remains to be seen whether dis-regulated miR-181a expression could lead to an impaired type 1 γδT cell surveillance in prostate and breast cancer.

Major points:

1. The authors show that γδ T cell counts are lower in the blood of metastatic cancer patients. This is likely due to the dose and type of anti-cancer therapy these patients have been receiving at the time of sampling. The authors have to discuss this and investigate whether the extend of γδ T cell reduction correlates with treatment intensity.

We thank the Reviewer for raising this important point. We have segregated samples in function of the type (or lack) of anti-cancer treatment and observed that hormone therapy (HT) and radiotherapy (RT) did <u>not</u> reduce γδ T cell counts when compared to patients that did not receive any treatment for the past 6 months before baseline blood collection (Figure R4). In this same line, treated groups also did <u>not</u> display a reduction in γδ T cell expression of NKG2D (Figure R4), although more samples within the radiotherapy and combination treatment group would be required to confirm this preliminary result. Of note, as previously noted in our response to

Reviewer 1, we also observed no significant differences in their expression of miR-181a (Fig R1, please see above). These data are now mentioned lines 96-97 in our revised manuscript.

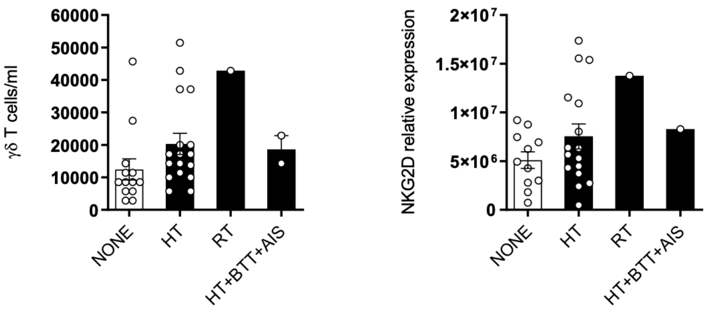

<u>Figure R4:</u> Influence of cancer treatment on γδ T cell counts (left) and NKG2D expression (right) in freshly isolated γδ PBLs from prostate cancer patients who received: no treatment in the previous 6 months before blood collection (NONE); Hormone Therapy (HT); Radiotherapy (RT); Bone-Targeted Therapy (BTT); or Anti-Inflammatory Steroid (AIS).

It is further unknown to which extend, if at all, the γδ T cell compartment in the peripheral blood is representative of the γδ T cell compartment in the tumour. If possible, it would be good to show increased miR-181a expression in γδ T cells from the TME of resected tumours from these patients.

We fully agree that tumour biopsies would be very interesting to analyze. Unfortunately, in this study and under its specific ethical approval, it was not possible to access such samples.

2. Only γδ T cells from a subset of metastatic cancer patients showed increased miR-181a expression. Is miR-181a expression correlated to TCRγ and TCRδ subsets? Or cytokine profile (steady state and after stim)? Or NKG2D expression?

We thank the Reviewer for this comment. As shown in Fig 1, higher miR-181a expression associated with lower NKG2D expression in patients with prostate cancer, when compared to healthy controls (Fig 1B). However, both parameters failed to correlate, mostly due to the variability introduced by three patients with very high miR-181a expression levels (Fig R5A). miR-181a expression also did not correlate with type 1 cytokine expression (Fig R5B-C), nor with Vδ1/Vδ2 ratio (Fig R5D). Of note, we found similar Vδ1/Vδ2 ratios in cancer patients and healthy controls (Figure R6).

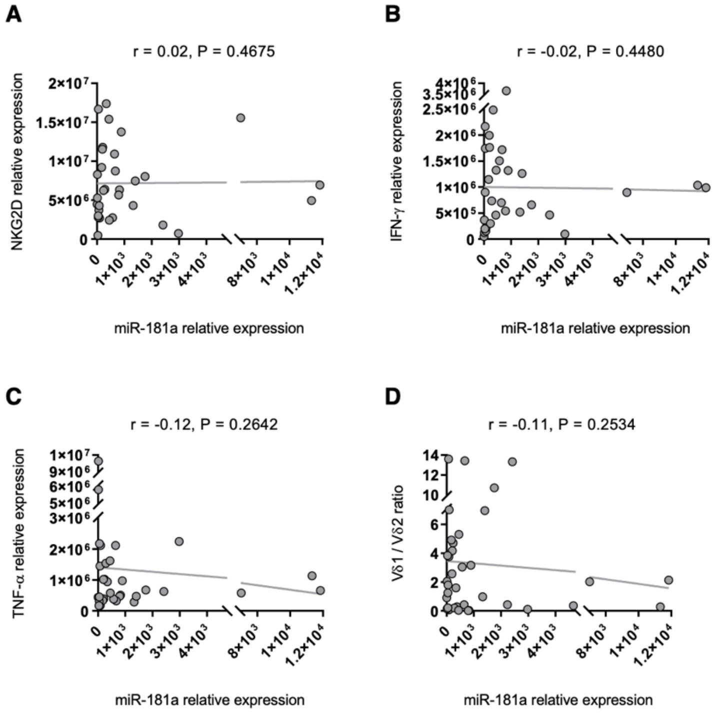

Figure R5: **miR-181a expression does not correlate with (A) NKG2D expression, (B) IFN-γ expression, (C) TNF-α expression, and (D) Vδ1 / Vδ2 ratio in freshly isolated γδ PBLs from prostate cancer patients.**

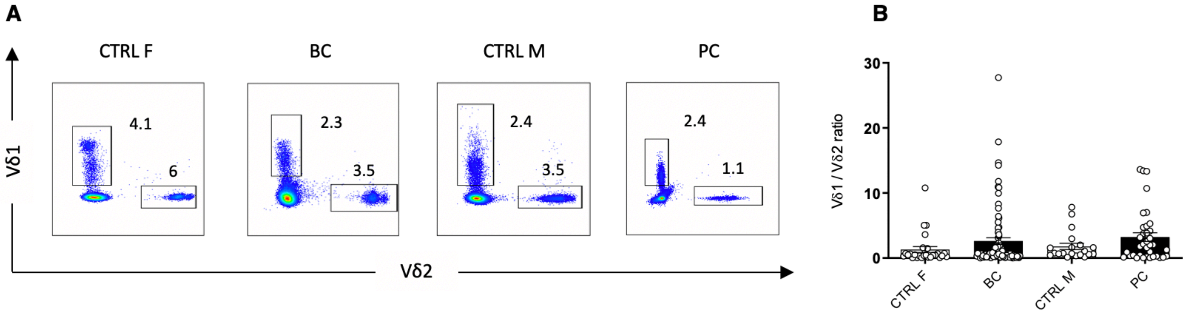

Figure R6: Vδ1^{+} *versus* Vδ2^{+} cells in freshly isolated γδ PBLs. (A) Representative dot plots for the indicated group samples. (B) Percentage of Vδ1^{+} *versus* Vδ2^{+} cells in freshly isolated γδ PBLs for the indicated group samples, namely Female healthy donors (CTRL F), patients with Breast Cancer (BC), Male healthy donors (CTRL M) and patients with Prostate Cancer (PC).

3. A major disconnect in the manuscript represents the author's observation of miR-181a expression in mature peripheral γδ T cells in prostate and breast cancer patients and the author's proposed molecular mechanism that they characterized using immature γδ thymocytes cultures in vitro. In γδ thymocytes expression of miR-181 is negatively correlated to type 1 effector phenotypes (NKG2D expression and production of IFN-γ, TNF-α upon stimulation) (Fig. 2D). By contrast, only NKG2D expression but not secretion of type 1 cytokines is inversely correlated with miR-181a expression in peripheral γδT cells

isolated from blood (Fig. 2E). These results suggest that miR-181a may not affect type 1 effector functions of differentiated mature γδT cells in the periphery. Thus, the authors should explain clearer how the miR-181a expression is dis-regulated in γδT cells in the peripheral blood of cancer patients. Moreover, the authors show in Fig. 3 only γδ thymocytes but not mature peripheral γδ T cells that overexpression of miR-181a in vitro compromise their type 1 effector functions. The authors should provide results showing the effect of miR-181a in vitro with peripheral γδ T cells from healthy individuals and/or cancer patients to demonstrate the pathological relevance of miR-181a in modulating the type 1 γδ effector functions.

We thank the Reviewer for bringing up this point that gave us the opportunity to provide additional insights on the regulation of miR-181a expression and potential consequences for pathophysiology.

We propose that the γδ T cell maturation status, which presumably associates with dynamic repertoires of mRNA targets, dictates sensitivity to miR181a. Contrary to their mature circulating counterparts, γδ T cells isolated from thymic biopsies are functionally immature and their type 1 differentiation is fully permissive to miR-181a regulation (Fig 3E). Our results in γδ thymocyte cultures indeed suggest that miR-181a controls γδ T cell expression of type 1 cytokines and NKG2D in an early time window.

We further show that high miR-181a expression associates with low NKG2D expression in circulating γδ T cells from patients with prostate cancer (Fig 1). Consistently, we also observe an inverse correlation between miR-181a and NKG2D expression levels in circulating γδ T cells from healthy donors (Fig 2F). However, whereas miR-181a expression inversely correlated with the expression of IFN-γ and TNF-α in γδ thymocytes (Fig 2E), it did not correlate nor associate with these hallmarks in circulating γδ T cells, whether they were isolated from healthy donors (Figure 2F) or from cancer patients (Fig EV1).

Altogether, we propose a two-step model of a dynamic miR181a based-regulation of γδ T cell differentiation, which would be finely tuned within pro- versus anti-inflammatory environments. This may have pathophysiological implications in cancer immunity and inflammatory disease settings, as now discussed in our revised manuscript (lines 327-337).

Along these lines and as requested by the Reviewer, we have overexpressed miR-181a in peripheral γδ T cells from healthy individuals and found a modest but significant decrease in the percentage of NKG2D expressing cells, while type 1 cytokine hallmarks remained unaffected. This is consistent with Figure 2 that shows an inverse correlation in peripheral γδ T cells between the expression of miR-181a and NKG2D, but not type 1 cytokine expression. These new data were added to our revised manuscript (Figure EV6 and lines 193-200).

We also followed the Reviewer's suggestion and speculated on the molecular mechanisms that dysregulate the expression of miR-181a in the peripheral blood from patients, namely in the context of prostate cancer (Fig 1D). For this, peripheral γδ T cells were isolated from healthy donors and cultured in the presence of anti-inflammatory cytokines, with the aim to mimic an immunosuppressive environment typical of a cancer setting. We observed that TGF-β increases miR-181a expression (Fig 2D) while decreasing NKG2D expression (Fig EV3). These results fit

the hypothesis of a TGF-β-mediated tumor evasion mechanism that hampers γδ T cell effector functions, as now discussed on lines 286-287 and 333-337 of the revised manuscript.

4. The chosen effectors downstream of miR-181a are finely picked. Have the authors analysed a wider range of potential targets?

We selected a list of miR-181a candidate mRNA targets based on (i) their high target score and number of predictor sources > 5 (miRDIP v4.1 analysis); (ii) gene set enrichment analysis (GSEA) focusing on type 1 differentiation pathway; and (iii) GSEA and COSMIC database analysis of genes dysregulated in cancer. From the intersection of these tables, we identified Map3k2, Notch2, Irf4 and Stat1 as one of the 12 most promising candidates. The detailed analysis can now be found in Dataset EV1.
Besides Map3k2 and Notch2, that were functionally validated as miR-181a targets (Fig. 4C), we also analyzed Irf4 and Stat1, as these two transcription factors are well known to play critical roles in T cell differentiation (O'Shea, NRI 2011, Huber, EJI 2014), with Irf4 also implicated in T cell exhaustion (Man, Immunity 2017). We found that miR-181a transduced γδ T cells displayed a significant decrease in expression of these candidate genes (Figure EV7A). However, and contrary to Map3k2 and Notch2, neither Irf4 nor Stat1 validated as miR-181a targets using luciferase assays (Figure EV7B). We now refer to these experiments on lines 234-239 of our revised manuscript.

Have they checked Id2 levels in miR-181a overexpressing cells?

We found that miR-181a transduced γδ T cells displayed reduced expression of Id2, when compared to empty vector transduced cells (Figure R7).
Our group has recently shown that miR-181a controlled the production of IFN-γ in murine CD8 T cells by targeting Id2 (Amado, 2020). However, in the present study, miR-181a overexpression did not affect the expression of IFN-γ by human γδ T cells (Figure 3D), which detracted us from further exploring Id2.

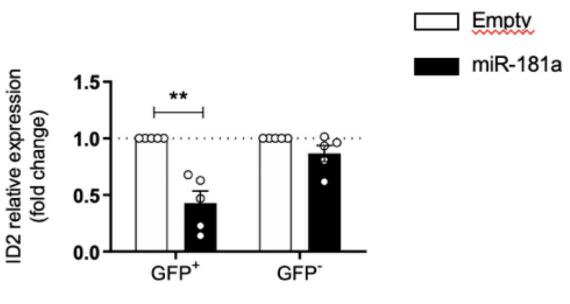

<u>Figure R7:</u> RT-PCR analysis of the expression of Id2 in miR-181a *versus* empty transduced (GFP^+) and untransduced (GFP^-) γδ thymocytes (data normalized to the values obtained with the empty virus). Paired Student's t test. **P < 0.01.

Why does miR-181a overexpression lead to increased apoptosis?

Consistent with the increase in Annexin V$^+$ cells within transduced γδ T cells, we observed that miR-181a overexpression induced a two-fold reduction in the expression of Bcl2 and Bcl-xL anti-apoptotic genes (Fig EV4).

Can miR-181a-insensitive Map3k2 and Notch2 mutants rescue the phenotype?

While we have not attempted to rescue the observed phenotype, we used a siRNA strategy to test whether silencing Map3k2 and Notch2 would phenocopy miR-181a overexpression. As we could not gain access to sufficient thymic biopsies within the timeframe of our manuscript revision, we used γδ T cells isolated from peripheral blood. We observed that siRNA targeting Map3k2 or Notch2 reduced NKG2D expression without affecting type 1 cytokine expression (Fig 4D), which was consistent with the results obtained from our miR-181 overexpression strategy (Fig EV6). Adding to the results obtained with luciferase assays (Fig 4C), these new data confirm that miR-181a targeting of Map3k2 and Notch2 impacts on γδ T cell differentiation. The results were added to our revised manuscript (Fig. 4D and lines 240-242).

Minor points:

1. Fig. 1: Please indicate which cancer patients are age-matched with which healthy controls.

We have clarified this issue in the material and methods section.

2. Have the authors investigated the expression of miR-181a in γδ T cells at earlier stages of cancer progression?

For the purpose of this study, we did not have access to peripheral blood samples at earlier stages of cancer progression. Our cohort only includes metastatic stage IV patients. Notwithstanding, we thank the Reviewer for this suggestion that we will assess in follow-up investigations.

3. Fig. 1: Please use the same y-axis for Fig. 1D and Fig. 1E. In the current version the figure is misleading and it is not clear whether for the majority of samples the statement in line 99-100 ("miR-181a expression was substantially higher in γδT cells when compared to their αβ T cell counterparts.")

We apologize for this inconsistency. The revised version of our manuscript has been corrected accordingly.

4. Lines 136-139: "Interestingly, IFN-γ and TNF-α expression did not correlate with miR-181a expression in ex vivo peripheral blood γδ T cells (Fig 2E), suggesting a role for miR-181a during the differentiation process, after which cytokine production becomes constitutive in mature cells." Could the authors clarify at which stage of the differentiation process, the miR-181a expression is affecting immature γδ thymocytes and mature peripheral γδ T cells differently? Immature γδ T thymocytes are affected before

lineage commitment and mature peripheral γδ T cells are affected only after the lineage is determined and constitutively secreting type 1 cytokines? Again, in this case, would the molecular mechanisms identified using γδ thymocytes be applicable to mature peripheral γδ T cells in cancer patients?

Having previously shown that γδ thymocytes are immature (Ribot, 2014), we now propose that miR-181a regulates an early step of peripheral γδ T cell differentiation (upon activation). Interestingly, we can also speculate that miR-181a, which is highly expressed by γδ thymocytes (Fig 2B), may act as a functional brake during γδ T cell development in the human thymus. This would explain why contrary to murine γδ T cells, that commit to effector functions in the thymus (Ribot, NI 2009), human γδ thymocytes remain immature (Ribot, 2014). We discuss these ideas in our revised manuscript (lines 259-263, 280-283, 297-300 and 327-337).
Due to their naïve phenotype, we believe that γδ thymocytes are a great tool to uncover molecular mechanisms of γδ T cell differentiation. We further believe that our findings are applicable to peripheral γδ T cells from cancer patients, at least to some extent, as we stated on lines 300-309: *"the inverse correlation between miR-181a expression and NKG2D levels was found in both differentiating γδ thymocytes and in circulating γδ T cells; and reduced NKG2D expression associated with high miR-181a levels in γδ T cells isolated from the blood of patients with prostate cancer. NKG2D plays a critical role in tumor surveillance, by promoting target recognition and cytotoxicity, and thus is particularly relevant to prevent tumor cell metastasis (Sivori et al, 2019). Furthermore, the NKG2D/NKG2DL pathway is being targeted for cancer treatment, and an improved understanding of its post-transcriptional regulation may open new avenues for next-generation immunotherapies."*

5. Fig. 2B: Peripheral γδ T cells seem to express miR-181a in a much wider range compared to γδ thymocytes. Could it be due to the composition of different γδ T cell subsets in different individuals? (see major point 2)

We thank the Reviewer for raising this point. We sorted circulating Vδ1$^+$ and Vδ2$^+$ T cells from buffy coat and observed that miR-181a was similarly expressed in both subsets (Figure EV2A). Furthermore, we observed no correlation between Vδ1/Vδ2 ratio and miR-181 expression, in healthy controls (Figure EV2B), as well as in cancer patients (please see Fig R5D above). Collectively, these data suggest that individual's Vδ-based subset composition does not influence the expression of miR-181a in total γδ T cells. This point is now mentioned on lines 119-126 of the revised version of our manuscript.

6. Fig. 2D: Do the authors find positive correlations between immature γδ T cell markers and miR-181a?

We observed a tendency for a positive correlation between γδ T cell miR-181a expression and the fraction of the CD27⁺CD45RA⁺ immature/ naïve compartment (Fig R8), but since this was not statistically significant, we did not add it to the paper.

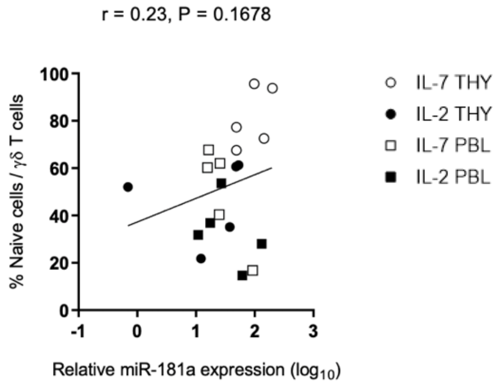

Figure R8: **Correlation between miR-181a and naïve γδ T cells isolated from thymic biopsies (THY) and peripheral blood (PBL), cultured with IL-7 or IL-2, as indicated.**

7. The authors should investigate whether modulation of miR-181a expression affects the differentiation of γδ T cells (thymocytes and/or peripheral cells) toward IL-17 lineages and their effector functions (eg. IL-17 secretion) upon in vitro stimulation.

We observed a very limited percentage (< 1%) of IL-17 expressing cells within our γδ T cell cultures and found no significant alteration in miR-181a transduced subset (Figure R9).
Notwithstanding, at this advanced stage of our study, we did not try to induce further IL-17 expression with a specific Th17 polarizing protocol. We thank the Reviewer for this suggestion that we propose to follow-up in future investigations.

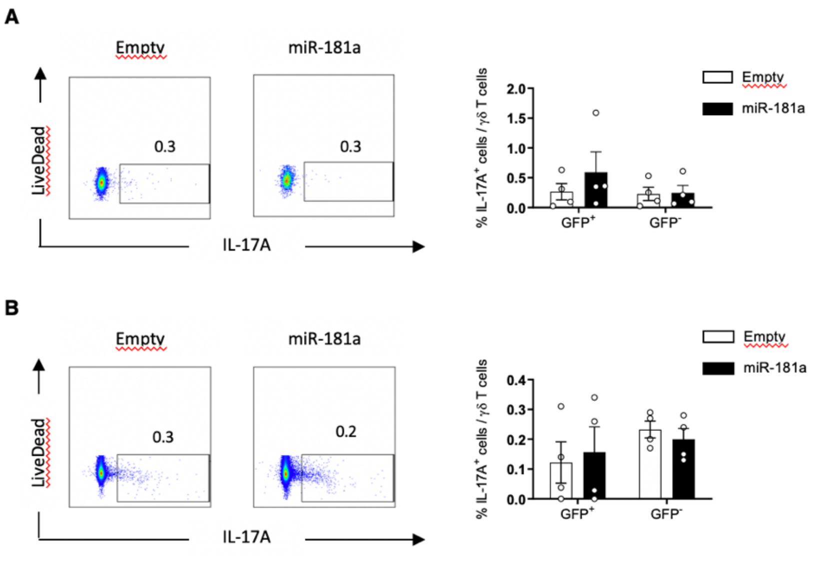

Figure R9: **IL-17A expression in miR-181a *versus* empty transduced (GFP⁺) and untransduced (GFP⁻) γδ T cells from (A) Thymus and (B) PBL samples.**

8. Please indicate in the methods how ecotropic pMIG-based retroviruses were made to infect human cells.

We have clarified this point in the Materials and Methods section of the reformulated manuscript (lines 416-429).

9. Fig. 3: Transduction of γδ T cells with pMIG does not result in discret GFP positive and negative subsets but rather a smear. Due to the IRES levels of GFP expression should correlate with levels of miR-181a expression. Have the authors gated on GFP high/medium/low cells overexpressing miR-181a and observed a dose response?

We thank the Reviewer for this suggestion. We actually do not observe a dose response, as GFP^{high} and GFP^{low} cells were both significantly affected by miR-181a overexpression (Fig R10).

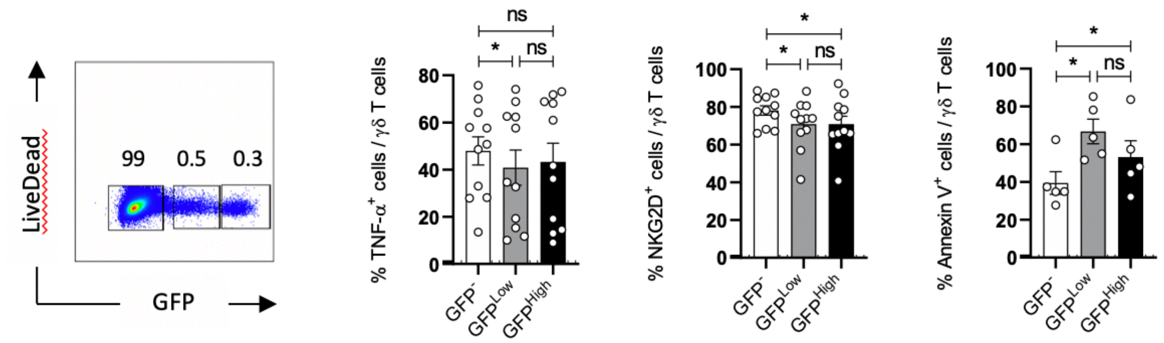

Figure R10: Impact of miR-181a levels on transduced γδ thymocytes. GFP^-, GFP^{Low} and GFP^{High} subsets were analyzed for their expression of TNF-α, NKG2D and Annexin V. Paired Student's t test. *P < 0.05.

10. In all qRTPCR experiments multiple housekeeping genes should be used to validate the results. Please indicate.

This information was added to our revised manuscript in the Materials and Methods section: "relative quantification of specific miRs to small nucleolar RNA C/D Box 44 (SNORD44) reference" and "relative quantification of specific cDNA species to endogenous references GUSB or PSMB6".

Referee #3 comments:

In this manuscript, Gordino and co-authors report that miR-181a inhibits functional maturation of human γδ T-cells via effects on Map3k2 and Notch2. Given the potent anti-tumor effector functions of the γδ T-cell lineage, these data aim to inform ongoing efforts to manipulate γδ T-cells for cancer treatment (currently a hot topic in immunotherapy). Consistent with this concept, the authors were able to identify that blood γδ T-cells from patients with metastatic prostate cancer display upregulation of miR-181a in parallel with reduced expression of the cytotoxicity mediator NKG2D. Differential expression levels of miR-181a between immature thymocytes and derivative peripheral T-cell populations has previously been identified in murine models (which the authors have referenced appropriately in the discussion). There remains substantial novelty in identifying if/how this molecule can modulate the γδ T-cell compartment in human blood and tissues, which is a major focus of current research in oncoimmunology. The data presented in support of the authors' claims are generally of good quality and presented in a clear, concise fashion throughout. Greater discussion of the possible interpretations of these findings would be beneficial.

It would have been valuable to determine if miR-181a only impacts on the Th1 functions of activated γδ T-cells, but the authors have not assessed alternative fates in their manuscript (previous work in murine models has also observed inhibition of conventional Th2 effector cells for example). Did the authors also attempt to establish whether γδ T-cell acquisition of Th17 effector functions can also be impaired by miR-181a? This would be interesting to know, although I suspect quite difficult to test given the challenges of inducing this γδ T-cell profile in a human system. For the short report format being used here, these data would probably qualify as 'nice to have' rather than critical to the authors' conclusions.

We thank the Reviewer for these remarks. As mentioned above (answering Reviewer 2 minor point 7), we observed a very limited percentage (< 1%) of IL-17 expressing cells within our γδ T cell cultures and found no significant alteration in miR-181a transduced subset (please see Figure R9 above). Likewise, we found IL-4 expression to be rare in our isolated γδ T cells (Figure R11), although we did not have the possibility to test the impact of miR-181a on this small IL-4$^+$ subset during the timeframe provided for our manuscript revision. We now mention these open questions on lines 277-279 of the discussion of our revised manuscript.

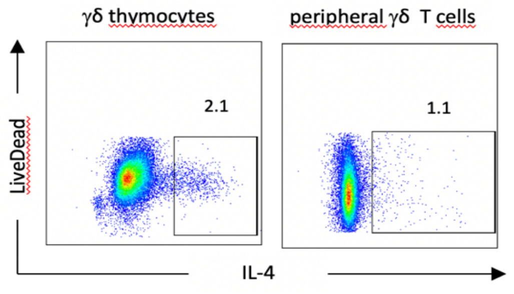

Figure R11: **IL-4 protein expression in γδ T cells freshly isolated from thymic biopsies (left) and peripheral blood (right).**

A significant omission is the lack of discrimination between Vδ1 and Vδ2 subsets in the authors' data (although they have clearly assessed these populations separately judging by the use of subset-specific antibodies in the methods section). The functional differences between these populations are significant enough that I would not anticipate uniform results for each across the various assay types employed in this report. In several of the graphs displayed, there appear to be both cytokine high and low expressing populations of γδ T-cells present, so it is possible that miR-181a does not impact the biology of both subsets equally. Currently, it is not possible to assess this because only global results for the total γδ T-cell pool are provided. If technical limitations necessitated the use of bulk γδ T-cell preparations in places, then this should be acknowledged / explained in the text. At present, the Vδ2 population is suddenly mentioned in line 164 of the manuscript without any further explanation, which will be difficult for a non-specialist audience to understand.

We thank the Reviewer for this important comment, which was also raised by Reviewer 2 in his/her major point 2 and minor point 5. As mentioned above, we observed that miR-181a was not differentially expressed in Vδ1 *versus* Vδ2 subsets (Figure EV2A), suggesting that the impact of miR-181a on γδ T cells would potentially be independent of their Vδ usage. Moreover, as assessed by TNF-α expression, both Vδ1 and Vδ2 thymocytes responded in a similar way to miR-181a transduction (Figure EV5). Furthermore, we observed no correlation between Vδ1/Vδ2 ratio and miR-181 expression, either in healthy controls (Figure EV2B) or in cancer patients (please see Fig R5D above). Collectively, these data suggest that our findings on miR-181a may be applicable to both γδ T cell subsets. We now further clarify this point on lines 119-126 and 191-192 of the revised version of our manuscript.

The influence of miR-181a appears to occur during γδ thymocyte differentiation rather than by impacting on the effector functions of mature cells. Consistent with this, the authors observed that IFNγ and TNFα expression by peripheral blood γδ T-cells did not correlate with miR-181a expression. This being the case, how do the authors propose that miR-181a inhibits the blood γδ T-cell pool of >50yo cancer patients? (who presumably have minimal thymic output by this age). Couldn't the reduced blood frequency of γδ T-cells in these individuals simply be a function of age? I suppose it is possible that γδ thymic emigrants might display increased miR-181a levels / defective Th1 function for a long period prior to oncogenesis. Alternatively, perhaps this miRNA species can indeed be induced in the periphery / is able to modify the activity of mature γδ T-cells in a cancer setting? Whatever their interpretation, it would be useful for the authors to discuss their findings as part of a broader framework that helps readers to understand the possible implications (assuming that space limitations don't prevent this).

We thank the Reviewer for this suggestion. We have assessed potential links between patient ages and γδ T cell numbers or miR-181a expression and found no significant correlations, in both cases (Figure R12). Instead, we speculate that an established cancer setting, rich in immunosuppressive cytokines, could enhance the expression of miR-181a in γδ T cells. As proof of concept, we have cultured peripheral γδ T cells in the presence of anti-inflammatory cytokines and observed that TGF-β indeed increased miR-181a expression (new Fig 2D), while reducing γδ T cell activation, as assessed by their expression of NKG2D (Fig EV3). This suggests a potential

tumor evasion mechanism in which miR-181a could inhibit γδ T cell activities (alike to "T cell exhaustion").

On the other hand, we also propose that miR-181a regulates an early step of γδ T cell differentiation upon activation in the periphery. Based on our thymocyte and PBL cultures, we suggest that the balance between pro- *versus* anti- inflammatory cytokines regulates miR-181a expression which finely tunes γδ T cell activation and type 1 differentiation. These points are now further discussed pages 11-12 our revised manuscript.

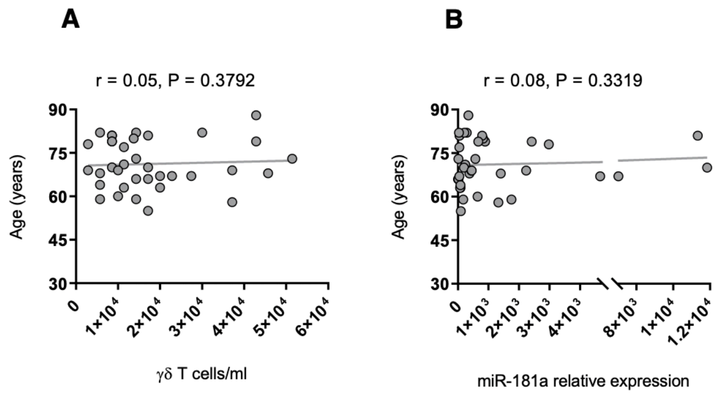

<u>Figure R12:</u> **Age does not correlate with (A) γδ T cell numbers nor (B) miR-181a expression in freshly isolated peripheral γδ T cells from PC patients.**

Minor points:

1. In Fig 3E, overexpression of miR-181a looks to have minimal impact on cytokine expression (TNF / IFNγ), while effects on other readouts appear extremely variable (NKG2D). It would be very useful to see how these results segregate between Vδ1 / Vδ2 lineages.

This has been addressed in response to the Reviewer's 2nd major point on page 16 above.

2. IL-7 is included for some experiments (γδ thymocyte transduction) but later omitted when molecular targets are being identified (Map3k2). Would be useful to explain the reasoning.

We apologize for this inconsistency. For a question of cell survival, γδ T cell cultures are always performed in the presence of IL-7, including in Fig. 4B, which legend has been corrected accordingly. On the other hand and following the manufacturer's instructions, luciferase assays were performed in the absence of cytokine.

3. Fig. 2A and C: Would be useful to see IL-2 and IL-15 data on the same graph (currently only displayed in isolation - how do these compare?)

We thank the Reviewer for this suggestion. We have altered Fig. 2C to display both cytokines in the same graph.

4. Line 106/107: Should specify pediatric biopsies *of thymus*.

Thank you, this has been corrected accordingly.

5. Line 180-186: The rationale for focusing on Notch and Mapk signaling pathways could be explained in more detail. What were the specific criteria applied / how were other things excluded?

We thank the Reviewer for this comment, which was also raised by Reviewer 2 (see his/her major point 4).
We selected miR-181a mRNA target candidates based (i) their high target score and number of predictor sources > 5 (miRDIP v4.1 analysis); (ii) gene set enrichment analysis (GSEA) focusing on type 1 differentiation pathway; and (iii) GSEA and COSMIC database analysis of genes dysregulated in cancer. From the intersection of these tables, we identified Map3k2, Notch2, Irf4 and Stat1 as one of the 12 most promising candidates (Dataset EV1). Of note, we had also selected the transcription factors Irf4 and Stat1, which are well known to play critical roles in T cell differentiation (O'Shea, NRI 2011, Huber, EJI 2014). Like with Notch2 and Map3k2, miR-181a transduced γδ T cells displayed a significant decrease in the expression of Irf4 and Stat1 (Figure 4B and Figure EV7A). However, luciferase assays, which confirmed Map3k2 and Notch2 as direct miR-181a targets (Figure 4C), failed to validate both Irf4 and Stat1 (please see Figure EV7B), which were therefore abandoned for the purpose of this study. We have added a brief mention to Irf4 and Stat1 on pages 8-9 of the revised manuscript.

6. Line 189: Specify the negative control (PTBP1)

Thank you, this has been corrected accordingly.

7. Line 289: Should read "diagnosed with stage IV breast *or* prostate cancer".

Thank you, this has been corrected accordingly.

8. Line 300: Should explain the "tissue dispersion" method in more detail.

We have removed "tissue dispersion" and replaced it by a brief description of the procedure: "Thymic samples were cut into small pieces (< 0,5 cm^2) and smashed onto a 70 μm filter".

9. Line 667: Add symbols for TNF-a and IFN-g.

Thank you, this has been corrected accordingly.

Dear Dr. Ribot,

Thank you for the submission of your revised manuscript to our editorial offices. We have now received the reports from the three referees that were asked to re-evaluate your study, you will find below. As you will see, the referees #2 and #3 now support the publication of your study. However, referees #1 and #2 have remaining concerns and suggestions to improve the manuscript, we ask you to address in a final revised manuscript. Please also provide a detailed point-by-point response to the points of referees #1 and #2.

Moreover, I have these editorial requests:

- Please provide the abstract written in present tense.

- We would like to publish your manuscript (as also indicated by you) as Report. However, for a Scientific Report we require that results and discussion sections are combined in a single chapter called "Results & Discussion". Please do this for your manuscript. For more details please refer to our guide to authors:
http://www.embopress.org/page/journal/14693178/authorguide#researcharticleguide

- For a report we only allow 5 EV figures, but there can be up to 5 main figures. Please arrange your display items accordingly. Finally, please update all the respective callouts.

- Please make sure that all figure panels are called out sequentially. It seems presently there are no separate callouts for the panels of Figs. EV2, EV4,E V5, EV6 and EV7. Please check.

- Regarding data quantification and statistics, please make sure that the number "n" for how many independent experiments were performed, their nature (biological versus technical replicates), the bars and error bars (e.g. SEM, SD) and the test used to calculate p-values is indicated in the respective figure legends (also of the EV figures). Presently many diagrams have no (or only partially) statistics. Please add statistical testing to all diagrams with n>2. Please also indicate (e.g. with n.s.) if testing was performed, but the differences are not significant.

- Please fuse the acknowledgements and the funding paragraph; please just call this part 'Acknowledgements'.

- Dataset EV2 is a small table related to the Methods part. Please add this as Table 1 to the main manuscript text, add a title and a legend, and add a callout to the respective section(s) of the methods.

- Finally, please find attached a word file of the manuscript text (provided by our publisher) with changes we ask you to include in your final manuscript text, and some queries, we ask you to address. Please provide your final manuscript file with track changes, in order that we can see any modifications done.

In addition, I would need from you:
- a short, two-sentence summary of the manuscript (35 words).
- three to four bullet points highlighting the key findings of your study.
- a schematic summary figure (synopsis image) in jpeg or tiff format with the exact width of 550 pixels and a height of not more than 400 pixels that can be used as a visual synopsis on our website.

I look forward to seeing the further revised version of your manuscript when it is ready. Please let me know if you have questions regarding the revision.

Kind regards,

Achim Breiling
Editor
EMBO Reports

Referee #1:

With their revised version, the authors provide an improved manuscript. They have adequately addressed some points raised by the reviewer. Most notably, they provide a much clearer conceptual connection between miR-181a action in thymus versus periphery.

However, experimentally, there is still little direct evidence for this connection. Notably, this is partially due to the fact that no new thymus tissue could be obtained because of the pandemic situation. The authors themselves refer to potential alterations in

context-dependent function of miR-181a due to alterations in the targetome. Does this also apply to the two characterized targets here? Or is it possible that thymocytes and PB gd T cells differentially depend on signals transmitted via those two targets? Also, the issue of differences between PC and BC derived gd T cells has not been resolved.

The authors have substantiated Map3k2 and Notch2 as putative targets using an siRNA approach in order to generate potential phenocopies, with which they partially succeeded. In addition, they demonstrated additional downregulation of downstream genes in new Figure 4. While this new data constitutes an improvement, there are still some issues: In 4D the authors should show a graph with a full y-axis starting at 0. Similar to the data shown in Fig. 3, the effect size is marginal. In Fig. 4B, it appears that the authors have accidentally mislabeled the central and right graphs. Also, whereas Hes1 is a prototypical transcriptional target of Notch signaling, Rbpj is not generally assumed to be under transcriptional control of Notch, but rather serves as a cofactor. Along the same line, whereas Atf2 is a key transcriptional target in the MAP kinase pathway, p38 is generally regulated through phosphorylation. Thus, this new data adds some confusion, especially when taking into account that no extrinsic Notch signal is provided in this assay. Is it possible to elicit a similar phenotype using gamma-secretase inhibitors? Overall, complementation experiments as suggested by one of the other reviewers are more appropriate to address this question.

With regard to a potential role of miR-181a-5p and the a-1 vs a-2 locus, the authors' response is not entirely satisfactory. They state that their vector is designed to specifically drive expression of miR-181a-2-3p. However, based on the primer sequences provided in Materials and Methods, this is not the case. These primers allow amplification of the complete miR-181a-2 hairpin including some flanking sequences. Thus, the vector encodes both a-2-5p and a-2-3p. Given the preferential recruitment of 5p into the RISC, it is most likely that this is a bona fide 5p expression vector (Of note, under Addgene #9044 the reviewer could only find the "classic" pMIG vector, not a vector called pMIG-PGW). If the authors wanted a 3p-specific construct, they would have needed to choose an artificial hairpin or single stranded mimics. In their Figure R3, it is evident that miR-181a-5p levels are roughly 30 times higher than those of 3p. The authors should provide copy numbers per cell in order to support their hypothesis of a prominent role of 3p. In order to assess, whether in fact, miR-181a-1 or a-2 are the relevant family members, qRT-PCR for mature miRNAs might not be sufficiently sensitive, because of the high degree of similarity (2 mismatches in 3p only). Rather, qRT-PCR for the pri-miRs would be more informative.

Referee #2:

The authors have substantially improved the original manuscript and included additional data.

Before publication the following points should be addressed:

1) Fig. 3D: miR-181a-IRES-GFP expression indicates two distinct populations with different miR levels (high and low, Fig.3B). Please gate on these populations individually and establish whether % of TNFa and IFNg+ cells correlate with this (Fig. 3D). Do the same for EV6 and also include non-transduced gd T cells to confirm correct gating on non-transduced cells.

2) Fig. 4D: show successful depletion of Notch2 and Map3k2 RNA/protein.

3) EV2 A: Has outlier analysis been performed? Additional samples/ repeats might proof useful to validate higher expression of miR-181a in Vd1 cells (which would fit very well with the authors hypotheses).

4) Correct statement line 156-158.

5) line 429: add which MOIs have been used and what transduction efficiencies were achieved

----

Referee #3:

My questions have been fully addressed and I have no further comments to add.

We thank all the Referees for their time evaluating our revised manuscript, EMBOR-2020-52234V2. We were pleased to read that we have addressed most of the concerns that were previously raised, namely by adding new data that substantially improved our original manuscript. We provide below a point-by-point reply to the issues remaining for Referees #1 and #2 in this second stage of revisions. New changes are highlighted in green in the updated manuscript.

Referee #1

With their revised version, the authors provide an improved manuscript. They have adequately addressed some points raised by the reviewer. Most notably, they provide a much clearer conceptual connection between miR-181a action in thymus versus periphery.

1) However, experimentally, there is still little direct evidence for this connection. Notably, this is partially due to the fact that no new thymus tissue could be obtained because of the pandemic situation. The authors themselves refer to potential alterations in context-dependent function of miR-181a due to alterations in the targetome. Does this also apply to the two characterized targets here? Or is it possible that thymocytes and PB gd T cells differentially depend on signals transmitted via those two targets? Also, the issue of differences between PC and BC derived gd T cells has not been resolved.

We thank the Reviewer for these comments. As previously stated, we did not have the opportunity to confirm that siRNA targeting Map3k2 and Notch2 in γδ thymocytes, as we show for peripheral blood γδ T cells. This notwithstanding, we observed that both Map3k2 and Notch2 expression is increased in functionally differentiated γδ thymocytes in the presence of IL-2, when compared to IL-7 control cultures (Figure R1). This upregulation upon differentiation associates with decreased miR-181a expression (Fig 2A). These data suggest that miR-181a may also regulate γδ thymocyte functional differentiation by targeting Map3k2 and Notch2.

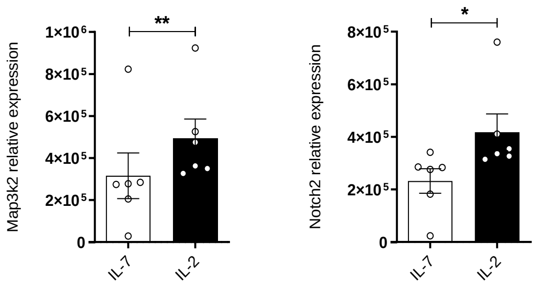

Figure R1: **RT-PCR analysis of Map3k2 and Notch2 expression in γδ thymocytes cultured with IL-7 *versus* IL-2. *P < 0.05 and **P < 0.01.**

Regarding the issue of differences between PC and BC derived γδ T cells, we understand that the Reviewer is referring to his previous comment, stating that elevated miR-181a levels are only observed in PC but not in BC γδ T cells. Since we could not find any influence of sex or cancer therapy (as detailed in our previous point-to-point reply), we assume that intrinsic characteristics of the respective cancer types account for these differences, which are out of the scope of our study.

2) The authors have substantiated Map3k2 and Notch2 as putative targets using an siRNA approach in order to generate potential phenocopies, with which they partially succeeded. In addition, they demonstrated additional downregulation of downstream genes in new Figure 4. While this new data constitutes an improvement, there are still some issues: In 4D the authors should show a graph with a full y-axis starting at 0. Similar to the data shown in Fig. 3, the effect size is marginal. In Fig. 4B, it appears that the authors have accidentally mislabeled the central and right graphs. Also, whereas Hes1 is a prototypical transcriptional target of Notch signaling, Rbpj is not generally assumed to be under transcriptional control of Notch, but rather serves as a cofactor. Along the same line, whereas Atf2 is a key transcriptional target in the MAP kinase pathway, p38 is generally regulated through phosphorylation. Thus, this new data adds some confusion, especially when taking into account that no extrinsic Notch signal is provided in this assay. Is it possible to elicit a similar phenotype using gamma-secretase inhibitors? Overall, complementation experiments as suggested by one of the other reviewers are more appropriate to address this question.

We thank the Reviewer for these comments.
The y-axis of our graph in former Figure 4D, now Figure 4F, has been corrected as suggested. Although we recognize the marginal effect size on the NKG2D protein expression data, we have consolidated these data with a new panel E showing a significant reduction of *Nkg2d* at the mRNA level.
As recommended by Reviewer 2, we further added a graph showing a 20%-30% downregulation of Notch2 and Map3k2 mRNA expression upon siRNA targeting (New Fig4D). This partial but significant depletion potentially explain the modest impact of Notch2 and Map3k2 siRNAs mentioned here by the Reviewer.
We had indeed mislabeled the central and right graphs in Fig4B, and thank the Reviewer for noticing this error. Following the criticism about these graphs, we agree that the analysis of Rbpj and p38 in our previous Figure 4B added some confusion, and thus have removed these data from the new Fig 4B.

In the context of our study, we did not assess the impact of exogenous Notch signals on γδ T cell differentiation into type 1 effectors (producing IFN-γ and TNF-α). This has been previously demonstrated by others, using OP9-DL1 monolayer to seed γδ thymocytes (Van Coppernolle, Leukemia, 2012), or γδ PBLs (Gogoi, JI, 2014) treated with gamma secretase inhibitors, as suggested by the Reviewer. Interestingly, the latter loss-of-function approach led to impaired type 1 differentiation, similarly to our miR-181a overexpression results on γδ thymocytes. Those two references have been added to our updated manuscript, lines 319-321.

3) With regard to a potential role of miR-181a-5p and the a-1 vs a-2 locus, the authors' response is not entirely satisfactory. They state that their vector is designed to specifically drive expression of miR-181a-2-3p. However, based on the primer sequences provided in Materials and Methods, this is not the case. These primers allow amplification of the complete miR-181a-2 hairpin including some flanking sequences. Thus, the vector encodes both a-2-5p and a-2-3p. Given the preferential recruitment of 5p into the RISC, it is most likely that this is a bona fide 5p expression vector (Of note, under Addgene #9044 the reviewer could only find the "classic" pMIG vector, not a vector called pMIG-PGW). If the authors wanted a 3p-specific construct, they would have needed to choose an artificial hairpin or single stranded mimics. In their Figure R3, it is evident that miR-181a-5p levels are roughly 30 times higher than those of 3p. The authors should provide copy numbers per cell in order to support their hypothesis of a prominent role of 3p. In order to assess, whether in fact, miR-181a-1 or

a-2 are the relevant family members, qRT-PCR for mature miRNAs might not be sufficiently sensitive, because of the high degree of similarity (2 mismatches in 3p only). Rather, qRT-PCR for the pri-miRs would be more informative.

We thank the Reviewer for these comments.

First, we apologize for this error and thank the Reviewer for insisting in its correction. Our vector indeed does not specifically drive expression of miR-181a-2-3p, as the primers allow for amplification of the complete miR-181a-2 hairpin. Thus, we cannot discard the contribution of the 5p strand in our assays. This point is now added to our updated revised manuscript (lines 363-365).
Of note, besides the retroviral transduction strategy, we had previously tried to electroporate γδ thymocytes with a (specific) miR-181a-2-3p mimic. While most of our attempts were unfruitful, mostly due to a low transfection efficiency and high toxicity for γδ T cells, some experiments pointed to a decrease in TNF-α expression levels in γδ thymocytes electroporated with miR-181a-2-3p mimic (Fig R2). This data is consistent with our results using the retroviral expression (Fig 3F), and thus support an important contribution of the miR-181a-2-3p strand regulating γδ T cell differentiation.

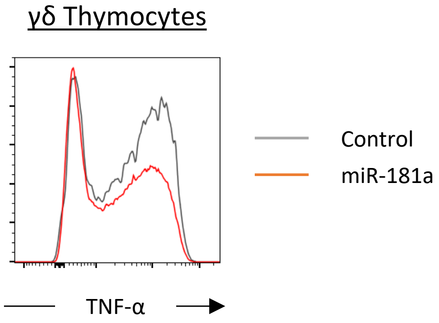

<u>Figure R2:</u> **FACS analysis of TNF-α expression in γδ thymocytes electroporated with a miR control mimic or miR-181a-2-3p mimic.**

Of note, although it has long been proposed that the 5p strand is the one being loaded into the RNA-induced silencing complex (RISC), recent evidence has disproven the idea that the 3p strand is mainly degraded during miR biogenesis (Kozomara, Nucleic Acids Res. 2019). Instead, both miR strands can be present and show differential expression levels according to the pathophysiological context under study (Mitra, Int J. Cancer, 2015). This is particularly the case in cancer, where genomic instability and epigenetic modifications influence the expression of precursor miRNA, resulting in altered levels of the 5p and 3p strands (Mitra, Int J. Cancer, 2015; Mitra, Nature Comm, 2020). These considerations were added to the introduction of our updated revised manuscript (lines 91-98)

While our overexpression strategy did not allow us to differentiate between the impact of the 3p and the 5p strands, we used primers specific for miR-181a-2-3p to report for miR-181a expression. Concerning the 3p strand origin being from miR-181a-1 or miR-181a-2, we acknowledge that the two mature sequences are similar, differing only in 3 nucleotides. To avoid miR misidentifications, we have chosen the LNA™ PCR primer sets from Qiagen (https://www.qiagen.com/us/products/discovery-and-translational-research/pcr-qpcr-dpcr/qpcr-assays-and-instruments/mirna-qpcr-assay-and-panels/mircury-

lna-mirna-pcr-assays/?clear=true#orderinginformation), which uses a technology with high specificity that discriminates closely related miRs and mature miR from precursors. Thus, we believe that a contribution of the 3p strand emerging from miR-181a-1 in our RT-PCR detection would be negligible.

Finally, we apologize for the incomplete description of the plasmid. The MSCV-IRES-GFP plasmid (pMIG #9044) was obtained from Addgene and has been modified to include a PGK-GFP-WPRE (PGW) sequence, hence the name pMIG-PGW. This information was added to the manuscript and can be found in lines 524-525.

Referee #2

The authors have substantially improved the original manuscript and included additional data.

Before publication the following points should be addressed:

1) Fig. 3D: miR-181a-IRES-GFP expression indicates two distinct populations with different miR levels (high and low, Fig.3B). Please gate on these populations individually and establish whether % of TNFa and IFNg+ cells correlate with this (Fig. 3D). Do the same for EV6 and also include non-transduced gd T cells to confirm correct gating on non-transduced cells.

Following the Reviewer's suggestion, we have gated on untransduced GFP$^-$, and transduced GFP^{low} and GFP^{high} populations of γδ thymocytes (from Fig 3) and PBLs (from former Fig EV6, now Fig EV4). However, we did not find any dose effect of the levels of miR-181a-bearing vector transduction (Fig R3), thus implying that the observed phenotypes (compared to control vector-transduced cells) are obtained with low miR-181a transduction and not maximized with higher levels of transduction.

We understand that the Reviewer is suggesting us to add this data as a Figure EV in our manuscript. However, we believe that the lack of significant differences between the untranduced GFP$^-$ samples and the transduced (GFP^{low} or GFP^{high}) samples for some conditions could be misleading for the reader. Instead, we put together a figure to show the absence of dose response using the more appropriate and widely accepted controls transduced with the Empty vector, and thus likewise submitted to retroviral infection. This data has been added to our revised manuscript as new Figure EV5.

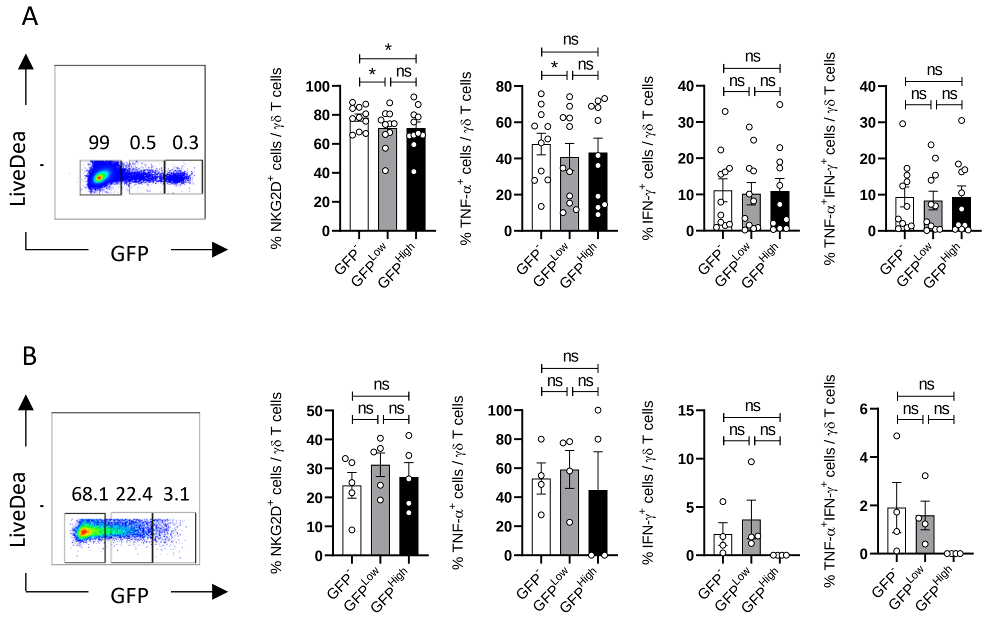

<u>Figure R3:</u> **Impact of miR-181a-bearing vector transduction levels on γδ T cells isolated from (A) thymus and (B) PBLs. GFP$^-$, GFP^{Low} and GFP^{High} subsets were analyzed for their expression of NKG2D, TNF-α and IFN-γ. *P < 0.05.**

2) Fig. 4D: show successful depletion of Notch2 and Map3k2 RNA/protein.

We thank the Reviewer for his comment.

We have added a new panel D to our Fig 4, that shows a 20%-30% downregulation of Notch2 and Map3k2 mRNA expression upon siRNA targeting. This significant albeit partial (as it is often the case with siRNA approaches) depletion may explain the also significant but somewhat marginal impact of Notch2 and Map3k2 siRNAs as previously mentioned by Reviewer 1.

3) EV2 A: Has outlier analysis been performed? Additional samples/ repeats might proof useful to validate higher expression of miR-181a in Vd1 cells (which would fit very well with the authors hypotheses).

We thank the Reviewer for this suggestion. We have performed an outlier analysis using the Grubbs's test (Alpha = 0.01) and also using the ROUT test (Q = 1%), as now stated in the revised Material and Methods. In both cases, an outlier was identified in the Vδ2$^+$ group. This point has been excluded and a new statistical analysis (unpaired t-test) indicates a slight but significant higher miR-181a expression in Vδ1$^+$ γδ T cells when compared to their Vδ2$^+$ counterpart (Former Fig EV2, now updated Fig EV1A). As the Reviewer anticipated, this result could indeed contribute to the differences observed between thymic and PBL γδ T cells. However, analyzing another set of samples, we did not observe any correlation between the Vδ1/ Vδ2 ratio and miR-181a expression (Fig EV1B). These considerations were added to our updated revised manuscript (lines 163-165)

4) Correct statement line 156-158.

We apologize for this error. The manuscript sentence has been altered as follows "[…] associated with a lower percentage of NKG2D$^+$ cells (line 203)."

5) line 429: add which MOIs have been used and what transduction efficiencies were achieved

We thank the Reviewer for his comment. We have added this information to our manuscript, which can be found on lines 543-544.

Dear Dr. Ribot,

Thank you for the submission of your revised manuscript to our editorial offices. I have now received the report from the two referees that were asked to re-evaluate your study, you will find below. As you will see, the referees are still not fully satisfied by the revision and have some further requests and suggestions to improve the study, I ask you to address in a further revised version of the manuscript.

I look forward to seeing the final revised version of your manuscript when it is ready. Please let me know if you have questions regarding the revision.

Yours sincerely,

Achim Breiling
Editor
EMBO Reports

Referee #1:

While the authors now provide preliminary data for the reviewer only that indeed miR-181a-2-3p might contribute to the observed effects in gd T cells, the overall issue of the role of 5p vs. 3p strands has not been adequately resolved. The reviewer acknowledges that 3p strands of an miRNA may contribute to biological function - prominent examples are miR-142 and miR-30. In addition, alterations in selective strand incorporation into the RISC may indeed occur. However, the authors do not demonstrate that this is the case here. Rather, in their reply, the refer to miRbase papers (Kozomara et al., 2019, 2014). However, miRbase data clearly indicate throughout for miR-181a that the 5p strand is the dominant one, which is consistent with the authors' own data presented in their first point-by-point reply, which indicated (given identical PCR efficiency) that the 5p strand is 30 times more prevalent than the 3p strand. Given the miRNA mechanism of action, a certain threshold intracellular concentration is required to generate a robust biological effect. This was recently validated in ImmGen's miRNA expression atlas paper in Nat Immunol (Brown et al., 2021). Therefore, the reviewer's request to provide copy numbers is still valid.

With regard to 3p-1 vs. 3p-2 discrimination the reviewer has to take at face value that the manufacturer's claims for specificity is correct. Substantiation of this claim by looking at pri-miR levels, which constitute a simple additional PCR, would have been more convincing.

With regard to Rbpj and p38 the reviewer considers it inadequate to simply remove data for the sake of simplicity, if such data may point to alternative hypotheses, e.g. that more global or upstream transcriptional or epigenetic alterations are in place.

As per another reviewer's request the authors added MOIs for viral infection. Unless the authors use a non-standard definition of MOI, the given values are implausible. Given that viral infection follows a Poisson distribution and that there are non-replicating particles, infection rates cannot exceed the frequency of viral particles, i.e. 1% for MOI=0.01 and 10% for MOI=0.1.

In summary, the paper's main claim that miR-181a contributes to human gd T cell differentiation is most certainly valid. However, whether that occurs via a miR-181a-3p-2-MAP3K2/Notch2 link remains to be established. It would be exciting to see more rigorous testing of this hypothesis.

Referee #2:

The authors have substantially improved the manuscript. The observation that MicroRNA-181a is regulating human gammadelta T cell differentiation is a nice conceptual extension of the known roles of MicroRNA-181a in the development and differentiation of other T cell lineages.
The correlative finding that gd T cells from patients with metastatic prostate cancer express higher levels of MicroRNA-181a and lower NKG2D compared to gd T cells from healthy controls cannot be developed further and may provide novel entry points into treatment of cancer patients and improve gd T cell therapy.

Remaining minor issues:

1) The finding that TGFb induces MicroRNA-181a expression in thymocyte and peripheral gd T cell cultures is very significant and should be validated with TGFb blocking antibodies in these cultures.

2) check statement in line 468: that MOIs of 0.01 and 0.1 leading to transduction efficiencies of 65-80% and 85-98% does not

make sense. Either dramatic cell death is going on in these cultures and transduction is preferentially occurring in the very few surviving cells or the MOIs have been calculated incorrectly.

3) Fig 3D: 40% of "live" gd T cells are early apoptotic (AnnV+) in the cultures irrespective of transduction. In order to firmly establish a moderate increase of AnnV+ cells in the MicroRNA-181a over-expressing gd T cells the authors should show the whole gating strategy to make ensure rigorous elimination of late-apoptotic cells. The staining protocol for the live/dead staining (RT or ice, 10 or 30min) should be included in the M&M).

4) Fig 3F: align graphs in bottom row.

We thank all the Referees for their time evaluating our revised manuscript, EMBOR-2020-52234V3. We were pleased to read that we have addressed most of the concerns that were previously raised, namely by adding new data that substantially improved our original manuscript. We provide below a point-by-point reply to the issues remaining for Referees #1 and #2 in this third stage of revision. New changes are highlighted in blue in the updated manuscript.

Referee #1

While the authors now provide preliminary data for the reviewer only that indeed miR-181a-2-3p might contribute to the observed effects in gd T cells, the overall issue of the role of 5p vs. 3p strands has not been adequately resolved. The reviewer acknowledges that 3p strands of an miRNA may contribute to biological function - prominent examples are miR-142 and miR-30. In addition, alterations in selective strand incorporation into the RISC may indeed occur. However, the authors do not demonstrate that this is the case here. Rather, in their reply, the refer to miRbase papers (Kozomara et al., 2019, 2014). However, miRbase data clearly indicate throughout for miR-181a that the 5p strand is the dominant one, which is consistent with the authors' own data presented in their first point-by-point reply, which indicated (given identical PCR efficiency) that the 5p strand is 30 times more prevalent than the 3p strand. Given the miRNA mechanism of action, a certain threshold intracellular concentration is required to generate a robust biological effect. This was recently validated in ImmGen's miRNA expression atlas paper in Nat Immunol (Brown et al., 2021). Therefore, the reviewer's request to provide copy numbers is still valid.

We have now performed an additional RT-PCR analysis of γδ thymocytes cultured with IL-7 vs IL-2, which includes standard curves generated using synthetic miRNA oligonucleotides. This allowed us to determine the absolute copy number quantification of each miR-181a strand, as depicted below (Figure R1). Of note, all three miR-181a strands (1-5p, 1-3p and 2-3p) were significantly downregulated upon IL-2 stimulation, in line with the main message of our paper. These results were added to the revised manuscript in the new Figure EV1 (please see below).

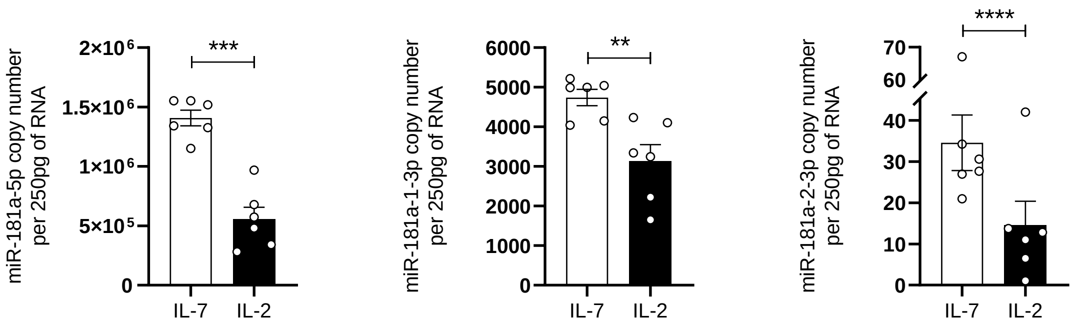

Figure EV1A: RT-PCR analysis of miR-181a-5p, miR-181a-1-3p and miR-181a-2-3p copy numbers in γδ thymocytes cultured with IL-7 *versus* IL-2. **P < 0.01, ***P < 0.001 and ****P < 0.0001.

With regard to 3p-1 vs. 3p-2 discrimination the reviewer has to take at face value that the manufacturer's claims for specificity is correct. Substantiation of this claim by looking at pri-miR levels, which constitute a simple additional PCR, would have been more convincing.

Given the shared similarities between the microRNA-181a-1-3p and 2-3p mature sequences, we agree with the Reviewer that looking at the pri-miR or pre-miR levels would be more accurate. We have therefore performed the suggested additional RT-PCR analysis to assess the expression levels of the pre-miRNA species based on the amplification of their stem-loop region, in γδ thymocytes cultured with IL-7 vs IL-2. We actually observed that both pre-miR-181-1-3p and 2-3p are significantly downregulated upon IL-2 stimulation. These results were also added to the revised manuscript in its new Figure EV1 (please see below).

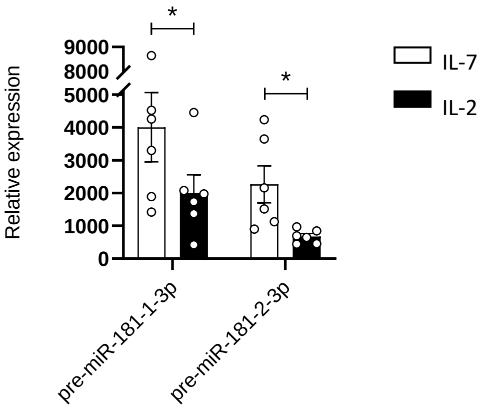

<u>Figure EV1B: </u>RT-PCR analysis of pre-miR-181a-1-3p and pre-miR-181a-2-3p expression in γδ thymocytes cultured with IL-7 *versus* IL-2. *P < 0.05.

With regard to Rbpj and p38 the reviewer considers it inadequate to simply remove data for the sake of simplicity, if such data may point to alternative hypotheses, e.g. that more global or upstream transcriptional or epigenetic alterations are in place.

We have now reintroduced this data in Figure 4B and discuss the data as suggested (lines 281-283).

As per another reviewer's request the authors added MOIs for viral infection. Unless the authors use a non-standard definition of MOI, the given values are implausible. Given that viral infection follows a Poisson distribution and that there are non-replicating particles, infection rates cannot exceed the frequency of viral particles, i.e. 1% for MOI=0.01 and 10% for MOI=0.1.

We apologize for this error. We have corrected this information in the manuscript as follows "Cells were infected at a multiplicity of infection of 2.5 or 5 which resulted in a transduction efficiency between 65-80% or 85-98%, respectively."

In summary, the paper's main claim that miR-181a contributes to human gd T cell differentiation is most certainly valid. However, whether that occurs via a miR-181a-3p-2-MAP3K2/Notch2 link remains to be established. It would be exciting to see more rigorous testing of this hypothesis.

Referee #2

The authors have substantially improved the manuscript. The observation that MicroRNA-181a is regulating human gammadelta T cell differentiation is a nice conceptual extension of the known roles of MicroRNA-181a in the development and differentiation of other T cell lineages.

The correlative finding that gd T cells from patients with metastatic prostate cancer express higher levels of MicroRNA-181a and lower NKG2D compared to gd T cells from healthy controls cannot be developed further and may provide novel entry points into treatment of cancer patients and improve gd T cell therapy.

Remaining minor issues:

1) The finding that TGFb induces MicroRNA-181a expression in thymocyte and peripheral gd T cell cultures is very significant and should be validated with TGFb blocking antibodies in these cultures.

We thank the Reviewer for his suggestion, that we addressed by adding a neutralizing anti-TGF-β antibody to peripheral γδ T cell cultures. As expected, we found that blocking TGF-β reversed the increase of miR-181a expression induced by TGF-β, reducing miR-181a expression to basal levels in γδ PBLs cultured with IL-7. These results were added to Figure 2D of our revised manuscript (please see below).

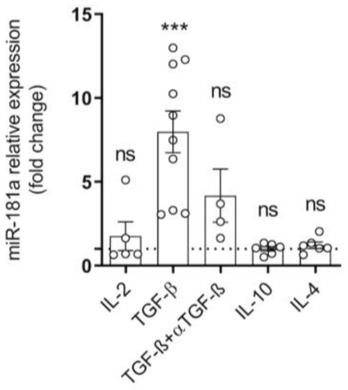

Figure 2D: RT-PCR analysis of the expression of miR-181a in γδ T cells isolated from PBLs and cultured with the indicated cytokines and an anti-TGF-β blocking antibody for 4-6 days.

2) check statement in line 468: that MOIs of 0.01 and 0.1 leading to transduction efficiencies of 65-80% and 85-98% does not make sense. Either dramatic cell death is going on in these cultures and transduction is preferentially occurring in the very few surviving cells or the MOIs have been calculated incorrectly.

We apologize for this error. We have corrected this information in the manuscript as follows "Cells were infected at a multiplicity of infection of 2.5 or 5 which resulted in a transduction efficiency between 65-80% or 85-98%, respectively."

3) Fig 3D: 40% of "live" gd T cells are early apoptotic (AnnV+) in the cultures

irrespective of transduction. In order to firmly establish a moderate increase of AnnV+ cells in the MicroRNA-181a over-expressing gd T cells the authors should show the whole gating strategy to make ensure rigorous elimination of late-apoptotic cells. The staining protocol for the live/dead staining (RT or ice, 10 or 30min) should be included in the M&M).

We have now included the Live/Dead staining protocol in the Materials and Methods section.

4) Fig 3F: align graphs in bottom row.

We thank the Reviewer for noticing this error. We have aligned the graphs accordingly.

3rd Revision - Editorial Decision 28th Sep 2021

Dear Dr. Ribot,

Thank you for the submission of your further revised manuscript to our editorial offices. I have now heard back from the two referees that were asked to look into this again. Please find their reports below.

As you can see, referee #2 now fully supports the publication of the paper. However, referee #1 is still not satisfied by the revisions. Nevertheless, as also this referee states that the main claim of the study (that miR-181a contributes to human γδ T cell differentiation) is valid and adequately demonstrated, I decided to proceed with publication, provided limitations and alternative conclusions (i.e. regarding the statement that miR-181a acts via a miR-181a-3p-2-MAP3K2/Notch2 link) are discussed in more detail in the final revised manuscript and further data on expression levels of the different miRNA miR-18a species (as indicated in your feedback letter) are included.

I would also suggest revising the title reflecting the comments of referee #1. Please also provide a detailed p-b-p-response addressing the report of referee #1 and detailing the further revisions undertaken.

I look forward to seeing the final revised version of your manuscript when it is ready. Please let me know if you have questions or comments regarding the revision.

Achim Breiling
Editor
EMBO Reports

Referee #1:

In their revised manuscript, the authors experimentally addressed in a more quantitative way the expression levels of miR-181a-5p, a-1-3p and a-2-3p. In support of miRbase data, miR-181a-5p is by far the most prominent species of the three and miR-181a-2-3p has the lowest levels. In fact, assuming an RNA content of 2-10pg/cell, miRNA levels determined by the authors correspond to approximately 12,000-60,000, 40-200, and 0.28-1.4 copies for 5p, 1-3p and 2-3p, respectively, prior to downregulation.

Unfortunately, the authors do not comment in their revised manuscript on the recent paper by Rose et al. (Nat Immunol 2021), which was highlighted by the reviewer and which directly associates miRNA function with expression levels. Despite the prominence of 5p and the reviewer's remarks, the authors have not altered their introductory section pertaining to the representation of 3p strands (page 3).

The authors have reintroduced their data on Rbpj and p38 and even indicate alternative conclusions of their data, but they remain vague and do not prompt the authors to critically reassess their study.

The reviewer's concluding remarks of the last round of revisions "In summary, the paper's main claim that miR-181a contributes to human gd T cell differentiation is most certainly valid. However, whether that occurs via a miR-181a-3p-2-MAP3K2/Notch2 link remains to be established. It would be exciting to see more rigorous testing of this hypothesis." were not commented upon at all in the revised version. Thus, it remains unclear what the authors' stance is on this issue, except for the fact that the manuscript's title remained unaltered.

The reviewer stands by his conclusions made in that statement:
The miR-181a-2-3p link is in fact implausible given the authors' own data in combination with the recent NI paper by Rose et al. Luciferase assays in HEK293 cells after overexpression are not suited to establish a physiological link. Neither do siRNA-based phenocopy searches. Rather, the authors seem to describe two effects: A role of miR-181a (5p?), which is strongly supported by data. Expression levels of key mediators in the Notch2 and MAPK pathways are altered through mechanisms that remain to be clarified. Given the broad nature of pathway regulation, including Rbpj and p38, it is unlikely that Notch2 and Map3k2 levels constitute upstream regulators in this mechanism.

Referee #2:

My concerns have been adequately addressed with the additional data shown and the correction of the manuscript text. I have no further comments to add.

Point-by-point reply to the remaining issues from Referee #1.
Changes are highlighted in grey in the revised manuscript.

Referee #1:

In their revised manuscript, the authors experimentally addressed in a more quantitative way the expression levels of miR-181a-5p, a-1-3p and a-2-3p. In support of miRbase data, miR-181a-5p is by far the most prominent species of the three and miR-181a-2-3p has the lowest levels. In fact, assuming an RNA content of 2-10pg/cell, miRNA levels determined by the authors correspond to approximately 12,000-60,000, 40-200, and 0.28-1.4 copies for 5p, 1-3p and 2-3p, respectively, prior to downregulation.

We acknowledge the Reviewer for this important consideration that we now discuss and experimentally address in the revised manuscript, as detailed below.

Unfortunately, the authors do not comment in their revised manuscript on the recent paper by Rose et al. (Nat Immunol 2021), which was highlighted by the reviewer and which directly associates miRNA function with expression levels. Despite the prominence of 5p and the reviewer's remarks, the authors have not altered their introductory section pertaining to the representation of 3p strands (page 3).

We thank the Reviewer for mentioning this very recent paper. We apologized that we have missed it, and we now mention and discuss this study in our revised manuscript (lines 335-341):
"Of note, a recent elegant study in mouse shows that miRNA activity is dependent on its concentration, and thus suggests that miRNA signatures should incorporate abundance thresholds to establish their regulatory relevance (Rose et al, 2021). On the other hand, an alternative view proposes that the gene regulatory function is a dynamic and complex process, in which a miRNA subcellular localization impacts its functionality, whereas expression levels only partially reflect its physiological effect (Lemus-Diaz et al, 2017)."

As suggested, we also developed our paragraph to introduce both miR-181a-5p and 3p species (lines 80-92):

"Each precursor miRNA consists of two mature RNA sequences - the 5p and 3p strands - whose designation is attributed according to the directionality of the miRNA strand (Kozomara & Griffiths-Jones, 2014). Although it has long been proposed that the 5p strand is the one being loaded into the RNA-induced silencing complex (RISC), recent evidence has disproven the idea that the 3p strand is mainly degraded during miR biogenesis (Kozomara et al, 2019). In fact, both the 5p and 3p strands can be loaded onto the Argonaute (AGO) family of proteins in an ATP-dependent manner (Yoda et al, 2010), and can show differential expression levels according to the pathophysiological context under study, namely in cancer (Mitra et al, 2015; Mitra et al, 2020). Importantly, miRNAs exert their regulatory functions in a highly combinatorial way: one miRNA can regulate several mRNAs in parallel, and different miRNAs can target one mRNA simultaneously, thus repressing its expression more efficiently (Pons-Espinal et al, 2017)."

We further insisted on the difference of their level expression in the context of γδ T cell differentiation (lines 332-335):

"Here we used specific primers to assess both miR-181a-5p and -2-3p expression in different γδ T cell samples and observed a consistent higher expression of the -5p compared to the -2-3p strand."

This notwithstanding, and given the most recent observations by Rose *et al*, we fully understood the Reviewer's concern about the potential lack of biological impact of miR-181a-2-3p (having the lowest levels, while miR-181a-5p is by far the most prominent species) and addressed his/her comment as follows:

First, we would like to highlight the particularly high expression of miR-181a-2-3p in γδ T cell samples freshly isolated from the PBLs of patients with prostate cancer (Fig 1E) or from healthy pediatric thymus (Fig 2A), when compared to culture samples (as the one we used to provide copy numbers in our previous reply). Thus, such increased levels could contribute to the biological effect of

miR-181a-2-3p in specific pathophysiological contexts, as we discussed lines 328-332: *"Several studies have highlighted the need to investigate the 3p strands, especially in a pathological context (Jazdzewski et al, 2008; Mitra et al, 2015; Pink et al, 2015; Misono et al, 2018; Mitra et al, 2020). In fact, a recent pan-cancer analysis has revealed a miR cooperativity of both (5p and 3p) strands to be able to regulate tumorigenesis and patient survival (Mitra et al, 2020)."*

Importantly, to experimentally address the Reviewer's issue, we measured the relative expression of miR-181a-5p in all γδ T cell samples that were available upon this revision time, including γδ thymocytes and γδ PBLs from patients with prostate cancer versus healthy controls. We now present a complete analysis of both miR-181a-5p and -2-3p expression in our updated Figure 1E and 2 A-F. As described along pages 5-7 of the revised paper, we show that:

- "among other candidates under study – miR-181a-5p and miR-181a-2-3p are both upregulated in metastatic cancer patients, especially in the prostate cancer cohort (Fig 1E)"

- "the expression of both miR-181a strands were significantly lower in in vitro differentiated (IL-2 cultured) γδ thymocytes compared to immature (IL-7 cultured) controls (Fig 2A)."

- "this downregulation of miR-181a (-5p and 2-3p) expression was also found when comparing freshly isolated (immature) γδ thymocytes versus (mature) peripheral γδ T cells ex vivo (Fig 2B)."

- "IL-15, which is also known to promote γδ T cell functional differentiation (Ribot et al, 2014), substantially downregulated the expression of both miR-181a strands. By contrast, TGF-β upregulated miR-181a (-5p and 2-3p) expression, while other cytokines such as IL-4, IL-12, IFN-γ and TNF-α showed no impact (Fig 2C)."

- "there was a striking inverse correlation between the expression of both miR-181a strands and the percentages of γδ thymocytes positive for IFN-γ, TNF-α and NKG2D (Fig 2E), fully consistent with our hypothesis that miR-181a negatively regulates type 1 effector γδ T cell differentiation."

The authors have reintroduced their data on Rbpj and p38 and even indicate alternative conclusions of their data, but they remain vague and do not prompt the authors to critically reassess their study.

We now added to our discussion on this point (lines 255-260):

"We further noticed a reduction of additional genes associated with both pathways, namely p38 and Rbpj (Fig 4B). Given the described role for both these genes as key regulators of DNA methylation and histone acetylation processes (Clark et al, 2003; Schmeck et al, 2005; Giaimo et al, 2017; Rozenberg et al, 2018), such results point to an alternative upstream transcriptional regulation or epigenetic alterations that can potentially be modulated by miR-181a upregulation."

Overall, we hope that this revised fifth version of our manuscript, which includes new experimental data and discussion points, has addressed all the remaining issues and that our manuscript is now suitable for publication.

Dear Dr. Ribot,

Thank you for the submission of your revised manuscript to our editorial offices. I have now received the report from the referee that I asked to re-evaluate your study (you will find enclosed below). As you will see, the referee has remaining concerns.

Please address the 2 referee points in detail and (if possible) with further data. If from the present data available it is not possible to clearly define which miRNA species targets both proteins, please clearly indicate and discuss this in the manuscript. Please also provide a point-by-point response to the referee points, including an explanation why the second potential miRNA-3-p2 binding site is not anymore mentioned in the manuscript. If there is no scientific reason for this, please reintroduce this site and discuss this issue in detail. Finally, please add the details indicated by the referee to the methods section

Moreover, we require an objective and comprehensive analysis of the literature, without any bias. Please remember that our reference lists are essentially unlimited and that we require the citation of all directly relevant primary research papers.

Please finally note that this is the last revision permissible at the journal. Please let me know if you have questions or comments regarding the revision.

Sincerely,

Achim Breiling
Editor
EMBO Reports

Referee #1:

In their latest iteration of this manuscript, the authors surprisingly for the first time indicate that the targets studied so far also contain binding sites for miR-181a-5p. Notably, given the 5p vs. 3p levels, with a substantial excess of 5p throughout all studied cell populations, this revelation makes the whole study much more plausible.

Nevertheless, some issues remain:

In Figure 4 the authors indicate the miR-181-5p binding sites in MAP3K2 and Notch2. However, they do not show new experiments. Having these new binding sites raises questions concerning the luciferase assay. In the methods section, unfortunately no details are provided. It must be assumed that the authors only mutated the 3-p2 binding sites (as 5p binding sites were not mentioned before), which resulted in complete derepression. This is inconsistent with the idea that 5p can physically target MAP3K2 and Notch2. The authors should provide more experimental details and an explanation. Also between versions of this manuscript, a second 3-p2 binding site has vanished from the manuscript (Figure 4A). How did the authors decide which one constituted the "main" binding site?

The authors discussed the recent paper by Rose and colleagues as requested. In this context, they unfortunately misrepresented the study by Lemus-Dias et al. (2017). The authors suggest that the latter provides somewhat opposing evidence. However, the opposite is clearly the case as Lemus-Dias and colleagues state: "The miRNAs UTA transfer functions show that low expressed miRNA below median have low or no inhibitory capability, while miRNAs over the median and third quartile show a range of behavior that does not reflect their expression level..." Thus, this study, like the study by Rose et al., highlights the notion that only highly expressed miRNAs have substantial repressive capacity. The restriction made by Lemus-Dias et al. only applies to those highly expressed miRNAs, which may be differentially post-transcriptionally regulated.

In summary, through five iterations the authors have formed a much more plausible manuscript, although the above-mentioned questions remain. It is a pity, that they decided to revise their manuscript in bits and pieces rather than once, but thoroughly, and with more attention to the current state of the art of miRNA biology and methodological detail.

Point-by-point reply to the remaining issues from Referee #1.

Changes are highlighted in orange in the revised manuscript.

Referee #1:

In their latest iteration of this manuscript, the authors surprisingly for the first time indicate that the targets studied so far also contain binding sites for miR-181a-5p. Notably, given the 5p vs. 3p levels, with a substantial excess of 5p throughout all studied cell populations, this revelation makes the whole study much more plausible.

Nevertheless, some issues remain:

In Figure 4 the authors indicate the miR-181-5p binding sites in MAP3K2 and Notch2. However, they do not show new experiments. Having these new binding sites raises questions concerning the luciferase assay. In the methods section, unfortunately no details are provided. It must be assumed that the authors only mutated the 3-p2 binding sites (as 5p binding sites were not mentioned before), which resulted in complete derepression. This is inconsistent with the idea that 5p can physically target MAP3K2 and Notch2. The authors should provide more experimental details and an explanation.

We thank the Reviewer for raising these questions and we wish to address any remaining concerns. Because it is technically impossible to clone the whole 3′ UTR region target genes, we have cloned 3’ UTR regions that include the predicted miR binding sites shown in Figure 4A. We actually adopted a two-step strategy for the luciferase assays. We first assessed potential direct binding between miR-181a-2-3p and our four mRNA target candidates (i.e. Map3k2, Notch2, Irf4 and Stat1) using their wild type 3’ UTR selected fragment and observed a significant reduction in the luciferase levels of Map3k2 and Notch2. We then validated these results for these two targets in particular. Importantly, the wild type and mutated 3’ UTR regions that were re-designed for this second round of luciferase assays actually contain both miR-181a-5p and -2-3p binding sites.

We do apologize for not having previously explained these experimental details and have now included this information in our revised manuscript (lines 280-284). As requested by the Reviewer, we have mentioned the mutated binding sites in the Materials and Methods section (lines 630-631). We have also updated the primer sequences of all the target candidates that were tested in our luciferase assays, namely adding Irf4, Stat1 and Ptbp1 (lines 619-629).

Finally, to further support direct binding between miR-181a(-5p and -2-3p) and Map3k2 or Notch2, we have correlated both -5p and -2-3p species expression levels in IL-7 *versus* IL-2 cultured γδ thymocytes (from Fig. 2A) with the expression levels of these two mRNA targets in these same samples. Interestingly, we report an inverse correlation between both miR-181a species and Map3k2, as well as Notch2 expression levels (please see our new Fig.4D). Of note, only the correlations for the 5p strand reached a statistical significance. This data – together with the previous observation of a predominance of this precise strand in our samples – supports the Reviewer's point that Map3k2 and Notch2 are more plausibly repressed by miR-181a-5p in a physiological context, as discussed lines 284-298.

Also between versions of this manuscript, a second 3-p2 binding site has vanished from the manuscript (Figure 4A). How did the authors decide which one constituted the "main" binding site?

We do apologize for this. We had decided to remove the second predicted binding site for miR-181a-2-3p in the 3' UTR of Notch2, mostly for a question of space limitation (having included a representation the 5p binding site) and because the primers that we used to amplify this target actually do not include this precise predicted binding site. Between the two predicted binding sites, we have chosen to amplify the 3' UTR region containing the higher percentile context score (as predicted by TargetScan v8.0), since this usually indicates a more favourable miR/mRNA interaction. However, we do understand and agree with the Reviewer that referring to this binding site as being the "main" binding site could be misleading, due to the complexity of miR-mediated regulatory processes. Thus, we have now reintroduced this binding site prediction in Fig. 4A, and we discussed this technical limitation in the revised version of our manuscript (lines 263-266).

The authors discussed the recent paper by Rose and colleagues as requested. In this context, they unfortunately misrepresented the study by Lemus-Dias et al. (2017). The authors suggest that the latter provides somewhat opposing evidence. However, the opposite is clearly the case as Lemus-Dias and colleagues state: "The miRNAs UTA transfer functions show that low expressed miRNA below median have low or no inhibitory capability, while miRNAs over the median and third quartile show a range of behavior that does not reflect their expression level..." Thus, this study, like the study by Rose et al., highlights the notion that only highly expressed miRNAs have substantial repressive capacity. The restriction made by Lemus-Dias et al. only applies to those highly expressed miRNAs, which may be differentially post-transcriptionally regulated.

We thank the Reviewer for raising this issue. We apologize for having unintendedly misinterpreted the study by Lemus-Dias and colleagues, and we do agree that their data indeed supports the recent publication by Rose and colleagues. We have now altered our citation to correctly represent these critical findings (lines 358-367).

Julie Ribot
Instituto de Medicina Molecular
Faculdade de Medicina da Universidade de Lisboa
Avenida Professor Egas Moniz
Lisboa, Lisboa 1649-028
Portugal

Dear Dr. Ribot,

I am very pleased to accept your manuscript for publication in the next available issue of EMBO reports. Thank you for your contribution to our journal.

At the end of this email I include important information about how to proceed. Please ensure that you take the time to read the information and complete and return the necessary forms to allow us to publish your manuscript as quickly as possible.

As part of the EMBO publication's Transparent Editorial Process, EMBO reports publishes online a Review Process File to accompany accepted manuscripts. As you are aware, this File will be published in conjunction with your paper and will include the referee reports, your point-by-point response and all pertinent correspondence relating to the manuscript.

If you do NOT want this File to be published, please inform the editorial office within 2 days, if you have not done so already, otherwise the File will be published by default [contact: emboreports@embo.org]. If you do opt out, the Review Process File link will point to the following statement: "No Review Process File is available with this article, as the authors have chosen not to make the review process public in this case."

Should you be planning a Press Release on your article, please get in contact with emboreports@wiley.com as early as possible, in order to coordinate publication and release dates.

Thank you again for your contribution to EMBO reports and congratulations on a successful publication. Please consider us again in the future for your most exciting work.

Yours sincerely,

Achim Breiling
Editor
EMBO Reports

****

THINGS TO DO NOW:

You will receive proofs by e-mail approximately 2-3 weeks after all relevant files have been sent to our Production Office; you should return your corrections within 2 days of receiving the proofs.

Please inform us if there is likely to be any difficulty in reaching you at the above address at that time. Failure to meet our deadlines may result in a delay of publication, or publication without your corrections.

All further communications concerning your paper should quote reference number EMBOR-2020-52234V6 and be addressed to emboreports@wiley.com.

Should you be planning a Press Release on your article, please get in contact with emboreports@wiley.com as early as possible, in order to coordinate publication and release dates.

EMBO PRESS

YOU MUST COMPLETE ALL CELLS WITH A PINK BACKGROUND ➔

PLEASE NOTE THAT THIS CHECKLIST WILL BE PUBLISHED ALONGSIDE YOUR PAPER

Corresponding Author Name: Julie C. Ribot
Journal Submitted to: EMBO Reports
Manuscript Number: EMBOR-2020-52234V2

USEFUL LINKS FOR COMPLETING THIS FORM

http://www.antibodypedia.com
http://1degreebio.org
http://www.equator-network.org/reporting-guidelines/improving-bioscience-research-repo
http://grants.nih.gov/grants/olaw/olaw.htm
http://www.mrc.ac.uk/Ourresearch/Ethicsresearchguidance/Useofanimals/index.htm
http://ClinicalTrials.gov
http://www.consort-statement.org
http://www.consort-statement.org/checklists/view/32-consort/66-title
http://www.equator-network.org/reporting-guidelines/reporting-recommendations-for-tun
http://datadryad.org
http://figshare.com
http://www.ncbi.nlm.nih.gov/gap
http://www.ebi.ac.uk/ega
http://biomodels.net/
http://biomodels.net/miriam/
http://jjj.biochem.sun.ac.za
http://oba.od.nih.gov/biosecurity/biosecurity_documents.html
http://www.selectagents.gov/

Reporting Checklist For Life Sciences Articles (Rev. June 2017)

This checklist is used to ensure good reporting standards and to improve the reproducibility of published results. These guidelines are consistent with the Principles and Guidelines for Reporting Preclinical Research issued by the NIH in 2014. Please follow the journal's authorship guidelines in preparing your manuscript.

A- Figures
1. Data
The data shown in figures should satisfy the following conditions:

- ➔ the data were obtained and processed according to the field's best practice and are presented to reflect the results of the experiments in an accurate and unbiased manner.
- ➔ figure panels include only data points, measurements or observations that can be compared to each other in a scientifically meaningful way.
- ➔ graphs include clearly labeled error bars for independent experiments and sample sizes. Unless justified, error bars should not be shown for technical replicates.
- ➔ if n< 5, the individual data points from each experiment should be plotted and any statistical test employed should be justified
- ➔ Source Data should be included to report the data underlying graphs. Please follow the guidelines set out in the author ship guidelines on Data Presentation.

2. Captions
Each figure caption should contain the following information, for each panel where they are relevant:

- ➔ a specification of the experimental system investigated (eg cell line, species name).
- ➔ the assay(s) and method(s) used to carry out the reported observations and measurements
- ➔ an explicit mention of the biological and chemical entity(ies) that are being measured.
- ➔ an explicit mention of the biological and chemical entity(ies) that are altered/varied/perturbed in a controlled manner.

- ➔ the exact sample size (n) for each experimental group/condition, given as a number, not a range;
- ➔ a description of the sample collection allowing the reader to understand whether the samples represent technical or biological replicates (including how many animals, litters, cultures, etc.).
- ➔ a statement of how many times the experiment shown was independently replicated in the laboratory.
- ➔ definitions of statistical methods and measures:
  - common tests, such as t-test (please specify whether paired vs. unpaired), simple χ2 tests, Wilcoxon and Mann-Whitney tests, can be unambiguously identified by name only, but more complex techniques should be described in the methods section;
  - are tests one-sided or two-sided?
  - are there adjustments for multiple comparisons?
  - exact statistical test results, e.g., P values = x but not P values < x;
  - definition of 'center values' as median or average;
  - definition of error bars as s.d. or s.e.m.

Any descriptions too long for the figure legend should be included in the methods section and/or with the source data.

In the pink boxes below, please ensure that the answers to the following questions are reported in the manuscript itself. Every question should be answered. If the question is not relevant to your research, please write NA (non applicable). We encourage you to include a specific subsection in the methods section for statistics, reagents, animal models and human subjects.

B- Statistics and general methods

Please fill out these boxes ➔ (Do not worry if you cannot see all your text once you press return)

1.a. How was the sample size chosen to ensure adequate power to detect a pre-specified effect size?	Power Analysis was performed prior to experiments in order to estimate the number of samples required to detect statistical significance (with population effect size d=1.2 to 1.7, 80% power and p<0.05; using Gpower software).
1.b. For animal studies, include a statement about sample size estimate even if no statistical methods were used.	NA
2. Describe inclusion/exclusion criteria if samples or animals were excluded from the analysis. Were the criteria pre-established?	We pre-established that cultured samples with a percentage of dead cells higher than 80% would be excluded from our analysis.
3. Were any steps taken to minimize the effects of subjective bias when allocating animals/samples to treatment (e.g. randomization procedure)? If yes, please describe.	We could not apply any randomisation procedure
For animal studies, include a statement about randomization even if no randomization was used.	NA
4.a. Were any steps taken to minimize the effects of subjective bias during group allocation or/and when assessing results (e.g. blinding of the investigator)? If yes please describe.	Results were analysed without particular blinding strategy
4.b. For animal studies, include a statement about blinding even if no blinding was done	NA
5. For every figure, are statistical tests justified as appropriate?	yes (please see each figure legend)
Do the data meet the assumptions of the tests (e.g., normal distribution)? Describe any methods used to assess it.	We used the D'Agostino-Pearson normality test when applicable. When the samples passed the normal distribution test, a parametric Student's t-test was used to assess for statistical significance between groups. When the samples did not follow the normality criteria, a non parametric Mann-Whitney U test was used instead.

Is there an estimate of variation within each group of data?	yes
Is the variance similar between the groups that are being statistically compared?	We assessed the variance between groups by using the F-test. In most cases, the samples dysplayed similar variances between groups. When the F-test returned a significantly different P-value ($P < 0.05$), we used a t-test with Welch correction.

C- Reagents

6. To show that antibodies were profiled for use in the system under study (assay and species), provide a citation, catalog number and/or clone number, supplementary information or reference to an antibody validation profile. e.g., Antibodypedia (see link list at top right), 1DegreeBio (see link list at top right).	please see our antibody list (Dataset EV2)
7. Identify the source of cell lines and report if they were recently authenticated (e.g., by STR profiling) and tested for mycoplasma contamination.	stated in our material and methods section

* for all hyperlinks, please see the table at the top right of the document

D- Animal Models

8. Report species, strain, gender, age of animals and genetic modification status where applicable. Please detail housing and husbandry conditions and the source of animals.	NA
9. For experiments involving live vertebrates, include a statement of compliance with ethical regulations and identify the committee(s) approving the experiments.	NA
10. We recommend consulting the ARRIVE guidelines (see link list at top right) (PLoS Biol. 8(6), e1000412, 2010) to ensure that other relevant aspects of animal studies are adequately reported. See author guidelines, under 'Reporting Guidelines'. See also: NIH (see link list at top right) and MRC (see link list at top right) recommendations. Please confirm compliance.	NA

E- Human Subjects

11. Identify the committee(s) approving the study protocol.	All samples were collected with informed consent and approved by the Ethical Board of the Academic Medical Center of Lisbon CAML (CHLN/FMUL/IMM).
12. Include a statement confirming that informed consent was obtained from all subjects and that the experiments conformed to the principles set out in the WMA Declaration of Helsinki and the Department of Health and Human Services Belmont Report.	Done
13. For publication of patient photos, include a statement confirming that consent to publish was obtained.	NA
14. Report any restrictions on the availability (and/or on the use) of human data or samples.	NA
15. Report the clinical trial registration number (at ClinicalTrials.gov or equivalent), where applicable.	NA
16. For phase II and III randomized controlled trials, please refer to the CONSORT flow diagram (see link list at top right) and submit the CONSORT checklist (see link list at top right) with your submission. See author guidelines, under 'Reporting Guidelines'. Please confirm you have submitted this list.	NA
17. For tumor marker prognostic studies, we recommend that you follow the REMARK reporting guidelines (see link list at top right). See author guidelines, under 'Reporting Guidelines'. Please confirm you have followed these guidelines.	NA

F- Data Accessibility

18: Provide a "Data Availability" section at the end of the Materials & Methods, listing the accession codes for data generated in this study and deposited in a public database (e.g. RNA-Seq data: Gene Expression Omnibus GSE39462, Proteomics data: PRIDE PXD000208 etc.) Please refer to our author guidelines for 'Data Deposition'.<br><br>Data deposition in a public repository is mandatory for:<br>a. Protein, DNA and RNA sequences<br>b. Macromolecular structures<br>c. Crystallographic data for small molecules<br>d. Functional genomics data<br>e. Proteomics and molecular interactions	NA
19. Deposition is strongly recommended for any datasets that are central and integral to the study; please consider the journal's data policy. If no structured public repository exists for a given data type, we encourage the provision of datasets in the manuscript as a Supplementary Document (see author guidelines under 'Expanded View' or in unstructured repositories such as Dryad (see link list at top right) or Figshare (see link list at top right).	Dataset EV1 included
20. Access to human clinical and genomic datasets should be provided with as few restrictions as possible while respecting ethical obligations to the patients and relevant medical and legal issues. If practically possible and compatible with the individual consent agreement used in the study, such data should be deposited in one of the major public access-controlled repositories such as dbGAP (see link list at top right) or EGA (see link list at top right).	NA
21. Computational models that are central and integral to a study should be shared without restrictions and provided in a machine-readable form. The relevant accession numbers or links should be provided. When possible, standardized format (SBML, CellML) should be used instead of scripts (e.g. MATLAB). Authors are strongly encouraged to follow the MIRIAM guidelines (see link list at top right) and deposit their model in a public database such as Biomodels (see link list at top right) or JWS Online (see link list at top right). If computer source code is provided with the paper, it should be deposited in a public repository or included in supplementary information.	NA

G- Dual use research of concern

22. Could your study fall under dual use research restrictions? Please check biosecurity documents (see link list at top right) and list of select agents and toxins (APHIS/CDC) (see link list at top right). According to our biosecurity guidelines, provide a statement only if it could.	NA
